# Glycolysis-associated lncRNAs identify a subgroup of cancer patients with poor prognoses and a high-infiltration immune microenvironment

**DOI:** 10.1186/s12916-021-01925-6

**Published:** 2021-02-25

**Authors:** Kuo-Hao Ho, Tzu-Wen Huang, Chwen-Ming Shih, Yi-Ting Lee, Ann-Jeng Liu, Peng-Hsu Chen, Ku-Chung Chen

**Affiliations:** 1 Graduate Institute of Medical Sciences, College of Medicine, Taipei Medical University, Taipei, Taiwan; 2 Department of Biochemistry and Molecular Cell Biology, School of Medicine, College of Medicine, Taipei Medical University, Taipei, Taiwan; 3 Department of Microbiology and Immunology, School of Medicine, College of Medicine, Taipei Medical University, Taipei, Taiwan; 4 Department of Neurosurgery, Taipei City Hospital Ren-Ai Branch, Taipei, Taiwan

**Keywords:** Long noncoding RNAs (lncRNAs), Glycolysis, MIR4435-2HG, Epithelial-to-mesenchymal transition (EMT), Immune infiltrations

## Abstract

**Background:**

Long noncoding (lnc)RNAs and glycolysis are both recognized as key regulators of cancers. Some lncRNAs are also reportedly involved in regulating glycolysis metabolism. However, glycolysis-associated lncRNA signatures and their clinical relevance in cancers remain unclear. We investigated the roles of glycolysis-associated lncRNAs in cancers.

**Methods:**

Glycolysis scores and glycolysis-associated lncRNA signatures were established using a single-sample gene set enrichment analysis (GSEA) of The Cancer Genome Atlas pan-cancer data. Consensus clustering assays and genomic classifiers were used to stratify patient subtypes and for validation. Fisher’s exact test was performed to investigate genomic mutations and molecular subtypes. A differentially expressed gene analysis, with GSEA, transcription factor (TF) activity scoring, cellular distributions, and immune cell infiltration, was conducted to explore the functions of glycolysis-associated lncRNAs.

**Results:**

Glycolysis-associated lncRNA signatures across 33 cancer types were generated and used to stratify patients into distinct clusters. Patients in cluster 3 had high glycolysis scores and poor survival, especially in bladder carcinoma, low-grade gliomas, mesotheliomas, pancreatic adenocarcinomas, and uveal melanomas. The clinical significance of lncRNA-defined groups was validated using external datasets and genomic classifiers. Gene mutations, molecular subtypes associated with poor prognoses, TFs, oncogenic signaling such as the epithelial-to-mesenchymal transition (EMT), and high immune cell infiltration demonstrated significant associations with cluster 3 patients. Furthermore, five lncRNAs, namely MIR4435-2HG, AC078846.1, AL157392.3, AP001273.1, and RAD51-AS1, exhibited significant correlations with glycolysis across the five cancers. Except MIR4435-2HG, the lncRNAs were distributed in nuclei. MIR4435-2HG was connected to glycolysis, EMT, and immune infiltrations in cancers.

**Conclusions:**

We identified a subgroup of cancer patients stratified by glycolysis-associated lncRNAs with poor prognoses, high immune infiltration, and EMT activation, thus providing new directions for cancer therapy.

**Supplementary Information:**

The online version contains supplementary material available at 10.1186/s12916-021-01925-6.

## Background

Cancer is regarded as a type of metabolic disease. Tumor cells can drive certain metabolic pathways to sustain their biological processes for growth and to adapt to complex tumor microenvironments (TMEs) [1]. A well-established metabolic pathway that plays a prominent role in cancer progression is glycolysis, which is critical for supplying energy and producing metabolic end products, thus maintaining tumor cell survival [2]. In addition to its functions in sustaining tumor growth, activation of glycolysis affects other phenotypic changes. For example, lactic acid produced by the glycolysis pathways induces the epithelial-to-mesenchymal transition (EMT) in lung cancer cells [3]. Furthermore, a pan-cancer study reported that activated glycolysis is correlated with increasing tumor immunity [4]. Hence, understanding the underlying relationship between glycolysis and cancer progression is a critical goal in cancer research.

Long noncoding (lnc)RNAs, which are longer than 200 nucleotides, can modulate gene expressions through various mechanisms. Several oncogenic signaling pathways, such as the cell cycle [5], immune regulation [6], and EMT mediation [7], are linked to lncRNA regulation. Different types of lncRNAs have been revealed to promote glycolysis activation. In breast cancer, lncRNA-SNHG7, which is promoted by c-MYC regulation, can increase glycolysis through inhibiting miR-34a-5p expression [8]. Another lncRNA, long intergenic noncoding RNA for IGF2BP2 stability, suppresses the degradation of IGF2BP2 by inhibiting the ubiquitination–autophagy pathway, leading to glycolysis upregulation in colorectal cancer [9]. However, most studies have focused only on particular lncRNA candidates and their functions in promoting glycolysis. Therefore, we systematically investigated the association of lncRNAs with glycolysis in different cancer types and explored their related signaling pathways and clinical relevance.

In this study, we used pan-cancer data from The Cancer Genome Atlas (TCGA) to identify glycolysis-associated lncRNAs across 33 tumor types. We performed a consensus clustering analysis to classify these glycolysis-associated lncRNAs into distinct clusters. We then identified glycolysis-associated lncRNAs that exhibited key clinical effects in five cancer types. Finally, we explored the potential pathways and functions of glycolysis-correlated lncRNAs in association with oncogenic signaling such as EMT and immune regulation.

## Methods

### TCGA pan-cancer data analysis and identification of cancer-expressing lncRNAs

Genomic profiles including RNA sequencing (RNA-Seq) data, gene-level copy number, DNA methylation, and patient clinical characteristics of 33 TCGA cancer types were downloaded from UCSC Xena (https://xena.ucsc.edu/). Raw counts of RNA-Seq data were normalized to counts per million (CPMs). Gene-level copy number values were calculated using GISTIC2.0. Beta values derived from Illumina human methylation 450K arrays were used to analyze TCGA DNA methylation changes. LncRNA annotation was retrieved from GENCODE, which contains 17,910 lncRNAs. In total, 15,121 lncRNAs were detected in TCGA RNA-Seq data. We defined an lncRNA as that expressed in a certain cancer type if the gene count was > 10 in more than 90% of patients.

### Identification of glycolysis-associated lncRNAs and categorization of cancers into subtypes

To evaluate the degree of glycolysis activation in cancer patients, expressions of glycolysis-involved genes, derived from the Hallmark glycolysis pathway [10], were examined to infer glycolytic activity through a single-sample gene set enrichment analysis (ssGSEA) method in the GSVA package. In each cancer type, we retained glycolysis-involved genes that were expressed in more than 90% of cancer patients with gene counts of > 10, and gene candidates that did not fit these criteria were excluded (Additional file 1: Table S1). The expressed lncRNAs were correlated with glycolysis scores in each cancer type by performing Pearson correlation analyses. LncRNAs with a false discovery rate (FDR) of < 0.05 and an absolute *R* of > 0.3 were considered to be significantly associated with glycolysis signaling. A consensus clustering method, according to de Jong et al.’s study [11], was performed to classify cancer patients into distinct clinically relevant subgroups based on glycolysis-correlated lncRNAs. Partitioning around medoids was used as the clustering algorithm. One thousand permutations with a 0.95 random fraction of lncRNAs in each iteration were repeated to perform the clustering analysis. Based on the delta area plot, the optimal cluster was selected according to whether no appreciable increase was present (Additional file 2: Figure S1). Then, survival differences among distinct clusters were evaluated using the log-rank test, and glycolysis scores within different groups were compared by employing the Kruskal–Wallis test with post hoc Dunn’s test.

### Development of genomic classifiers in bladder carcinoma and low-grade gliomas

To characterize genetic signatures permitting the distinguishing of glycolysis-associated lncRNA-based clusters in cancer patients, gene candidates that exhibited a distinct expression pattern among three clusters were identified. Specifically, we selected gene candidates that were consistently upregulated (FC > 1.5 and FDR < 10^− 4^) or downregulated (FC < 0.7 and FDR < 10^− 4^) in cluster 3 versus cluster 2, and cluster 2 versus cluster 1. In total, 174 upregulated and 49 downregulated genes were identified in low-grade gliomas (LGGs; Additional file 3: Table S2). In bladder carcinoma (BLCA), 121 genes were upregulated and 112 downregulated (Additional file 3: Table S3). A lasso penalized multinomial logistic regression with 10-fold cross-validation was performed to shrink the number of these gene candidates. After identifying the lambda value that provided a minimum mean cross-validated error, the value was applied to penalize our gene signature and thus build the genomic classifier. The 233 gene candidates in BLCA and 223 gene candidates in LGG were shrunk to 26 and 46 gene candidates, respectively (Additional file 4: Table S4 and S5). Then, this genomic classifier was used with the independent microarray data of glioma (GSE16011 and GSE107850) and BLCA (GSE48075 and GSE13507). However, due to the presence of different platforms for the TCGA RNA-Seq data and GEO microarray data, we performed quantile normalization on both the datasets by using preprocessCore package before building the genomic classifier.

### Comparisons of molecular differences including genomic mutations and molecular subtypes within different glycolytic signature-classified groups of cancers

Genomic mutation data of BLCA, LGGs, mesotheliomas (MESOs), pancreatic ductal adenocarcinomas (PAADs), and uveal melanomas (UVMs) were retrieved from UCSC Xena (https://xena.ucsc.edu/). These mutation data, generated by the Multi-Center Mutation Calling in Multiple Cancers (MC3) project, were derived from exon sequencing of TCGA cancer patient samples, and genes were categorized into binary calls as either nonsilent mutation or wild-type. Fisher’s exact test was performed to investigate genomic mutations that were significantly enriched (with a *p* value of < 0.01) in glycolysis score-classified cluster 3 or cluster 1 cancer patients. The identified mutation within each cancer type was shown as a heatmap. To compare established molecular subtypes with the glycolysis signature-classified groups, we selected two cancer types (LGG and BLCA) to perform the analyses; the types have been classified as different groups in other studies [12, 13]. Fisher’s exact test was conducted to examine whether glycolysis score-stratified cluster 3 or cluster 1 patients were enriched in certain molecular types.

### Differentially expressed gene analysis, GSEA, and TF activity scoring

To explore the transcriptome that exhibits distinct expression patterns within glycolysis score-stratified clusters, we used CPM-normalized counts from RNA-Seq data to perform a differentially expressed gene (DEG) analysis by using the edgeR package. Gene candidates were ranked based on log2 multiples of change to conduct a GSEA with 10^4^ permutations to calculate the normalized enrichment score (NES), and a pathway with an FDR of < 0.01 was considered significant enrichment. The Hallmark pathway database [10] was utilized to perform the analysis. To explore signaling pathways that were activated in cluster 3 cancer patients, activated signaling pathways in the poor prognosis patients (cluster 3) compared with good prognosis patients (cluster 1) were shown as a heatmap. To identify signaling pathways exhibiting different degrees of activation among distinct cluster cancer patients, we presented NESs derived from cluster 3 versus cluster 1 and cluster 2 versus cluster 1 in radar plots. To identify activated transcription factors (TFs) in cluster 3 cancer patients compared with cluster 1 patients, we scored TF activity by following a methodology developed by Garcia-Alonso et al. [14]. In brief, they defined a set of high-confidence human TFs and their target genes based on public resources including TF-binding site predictions, text-mining-derived and manually curated TF-target interactions, and chromatin immunoprecipitation coupled with high-throughput data (ChIP-X). RNA expressions of these TF targets were utilized to infer TF activity per patient by using analytical rank-based enrichment analyses (aREAs). TF activation levels among clusters were compared using analysis of variance (ANOVA), and an effect size of > 0.5 was considered a difference. Upregulated TFs in cluster 3 patients were used to perform a functional annotation test by using DAVID (https://david.ncifcrf.gov/tools.jsp).

### Investigation of localization of glycolysis-associated lncRNAs

The lncALTAS database [15] was used to investigate the possible localization of glycolysis-associated lncRNAs in cancer cells. This database contains RNA-Seq data from the cytoplasmic and nuclear compartments of 15 cell lines. In total, 6768 GENCODE-annotated lncRNAs were detected. Furthermore, a relative concentration index (RCI) was developed to characterize the distribution of lncRNAs in nuclear and cytoplasmic regions. In brief, this index was calculated using the log ratio of the concentration of a given RNA molecule per unit mass of RNA between its cytoplasmic and nuclear compartments. A positive RCI value indicates that an lncRNA has a higher concentration in the cytoplasmic region. By contrast, a negative value means that an lncRNA is more abundant in the nuclear region.

### Calculation of immune cell infiltration scores and an unsupervised hierarchical clustering analysis

To infer immune cell infiltration based on transcriptome profiles, we followed the method reported by Şenbabaoğlu et al. [16]. Briefly, different gene markers of immune cells were utilized as gene sets and used to perform an ssGSEA to obtain an immune cell infiltration score for each patient. Then, these immune cell infiltration scores were utilized to perform unsupervised hierarchical clustering. The distance of each patient was calculated using the Euclidean method, and clustering was performed using Ward’s method. Results are illustrated as a heatmap by using the complex heatmap package. For immune checkpoint comparisons, we queried the immune-suppressive checkpoint gene list from HisgAtlas [17], and we conducted ANOVA to compare their expressions in different clusters stratified by glycolysis scores. Checkpoints with an effect size of > 0.5 and a *p* value of < 0.01 were considered to significantly differ among the three clusters.

### First-order partial correlation and multivariate linear regression analysis

A first-order partial correlation was performed to explore interlinks among lncRNAs, glycolysis scores, and glycolysis-associated genes. The glycolysis score was assumed to be *x*, and glycolysis-associated gene expression was *y*. The first-order partial correlation between *x* and *y* conditioned on lncRNAs was

$$ {r}_{\mathrm{xylncRNA}}=\frac{r_{xy}-{r}_{\mathrm{xlncRNA}}{r}_{\mathrm{ylncRNA}}}{\sqrt{\left(1-{r^2}_{\mathrm{xlncRNA}}\right)\left(1-{r^2}_{\mathrm{ylncRNA}}\right)}} $$

We compared the cumulative distribution of Pearson correlation coefficients between glycolysis scores and gene expressions with or without removing the effect of lncRNA expression by using the Kolmogorov–Smirnov test. To identify gene candidates that were correlated with lncRNA expression and were independent of copy number variations and DNA methylation, we performed a multivariate linear regression to adjust for these covariates. We considered a gene candidate to be significantly associated with an lncRNA when its absolute correlation coefficient was > 0.3 and its FDR was < 10^− 6^.

## Results

### Identification of cancer types that can be classified into distinct prognostic groups based on glycolysis-associated lncRNAs

**Fig. 1:**
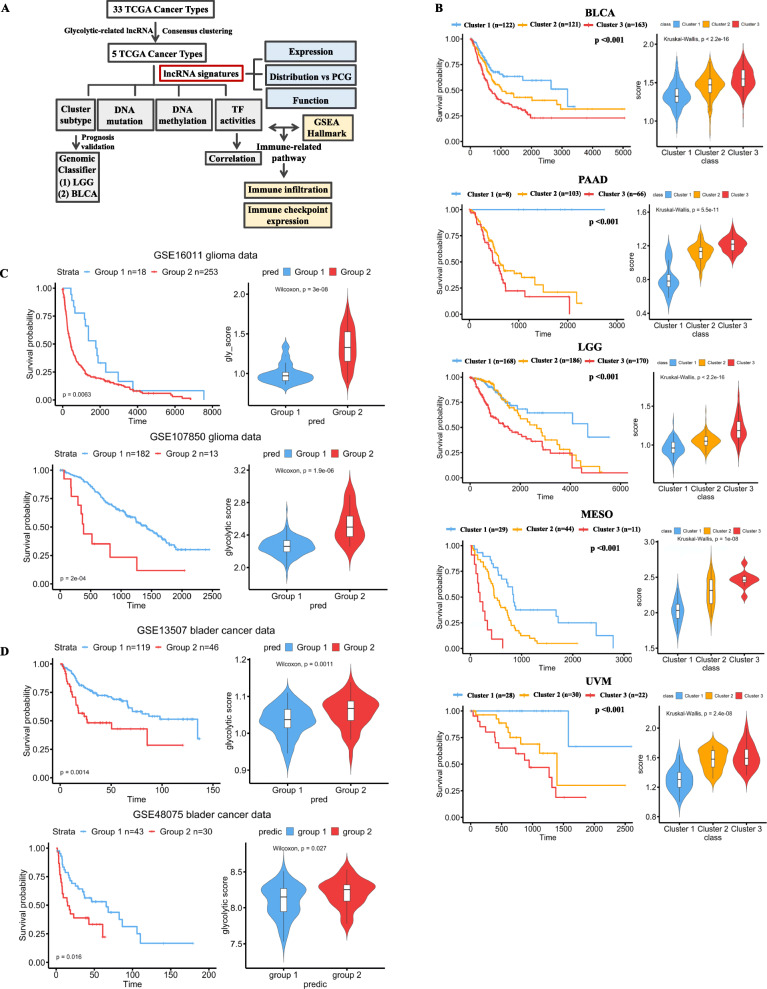
Identification of glycolysis-associated long noncoding (lnc)RNAs and their roles in cancer prognoses. **a** A flowchart demonstrating our investigation of glycolysis-associated lncRNAs and their putative functions in cancers. **b** Kaplan–Meier plots indicating patient survival rates of distinct clusters stratified by glycolysis-associated lncRNAs in five cancer types. Right panel presents glycolysis scores in different clusters. Cancer patients were clustered into groups based on glycolysis-associated lncRNAs by performing a consensus clustering analysis. Optimal clusters were selected by judging delta area plots where no appreciable increase was evident. Validation of the clinical importance of these glycolysis-associated lncRNAs was derived from genomic classifiers from independent GEO data including GSE16011 and GSE107850 for gliomas (**c**) and GSE13507 and GSE48075 for bladder cancer (**d**). Kaplan–Meier plots demonstrate patient survival rates of different groups. The right panel displays glycolysis scores in different clusters. TCGA, The Cancer Genome Atlas; GSEA, gene set enrichment analysis; PCG, protein coding gene; TF, transcription factor; BLCA, bladder carcinoma; LGG, low-grade glioma; MESO, mesothelioma; PAAD, pancreatic ductal adenocarcinoma; UVM, uveal melanoma

To systematically investigate the roles of glycolysis-associated lncRNAs in different cancer types, we analyzed TCGA pan-cancer RNA-Seq data that contained 33 tumor types from 10,121 tumor samples (Fig. 1a, Additional file 5: Table S6). We used genes derived from Hallmark glycolysis and performed an ssGSEA to infer glycolytic activity (Additional file 6: Figure S2). By performing the Pearson correlation analysis, we identified 1420 lncRNA candidates that were associated with glycolytic activity in at least one cancer type. Most (58.6%) of these lncRNAs were specifically correlated with glycolysis in one cancer type. Because no preexisting clusters were defined based on glycolysis-associated lncRNAs, we employed an unsupervised machine learning algorithm, consensus clustering, to define these subgroups (Additional file 7: Figure S3). Distinct cluster groups were classified for each cancer type. Most of the lncRNAs in cluster 3 were found to be downregulated. The overall survival among these cluster groups in each cancer type was also compared using the log-rank test (*p* < 0.01). Cluster 3 patients in five cancer types—BLCA, LGGs, MESOs, PAADs, and UVMs—demonstrated a significant association with a poor prognosis (Fig. 1b, left panel, Additional file 8: Table S7). Thus, we majorly focused the roles of glycolysis-associated lncRNAs on these five cancer types. The results indicated that the poor prognosis cluster group exhibited the highest glycolysis score across these five cancer types (Fig. 1b, right panel), implying that lncRNAs interact with glycolysis and cancer malignancy in these five cancers.

To validate the clinical significance of these lncRNA-defined groups in cancers, we applied a lasso penalized logistic regression, which was used for the preexisting label classification to train our classifiers. Although we attempted to validate the importance of these glycolysis-associated lncRNA-stratified clusters with other independent data, we discovered that public datasets were established using microarray platforms that lacked the expression information of numerous lncRNA candidates. Therefore, we developed a genomic classifier based on the protein coding gene signature that exhibited a distinct pattern among different clusters for validation with the microarray data. Because sample sizes for PAAD (*n* = 177), MESO (*n* = 84), and UVM (*n* = 80) were small, we mainly focused on the effects of lncRNAs on BLCA (*n* = 406) and LGG (*n* = 524). Furthermore, the microarray data of PAAD, MESO, and UVM cancer patients lacked survival information, limiting our validation of the clinical importance of glycolytic lncRNA-stratified clusters. Thus, we focused mainly on LGG and BLCA to validate the clinical significance of the clusters. We identified 26 gene candidates in BLCA and 45 gene candidates in LGGs by using the developed genomic classifiers, which allowed discrimination within these subgroups based on gene expression levels (Additional file 9: Figure S4, Additional file 4: Table S4 and S5). We then classified LGG and BLCA patients based on distinct clinical features. The results obtained from analyzing two independent databases for LGGs (Fig. 1c) and BLCA (Fig. 1d) indicated that patients with poor prognosis had high glycolysis scores. Moreover, we found that not all the gene candidates that were selected using genomic classifiers belonged to glycolysis pathways, which suggested that glycolysis-associated lncRNAs can be linked to other, nonglycolytic signal pathways to promote cancer malignancy.

### Molecular characterization of subgroups classified by glycolysis-associated lncRNAs in cancers

**Fig. 2:**
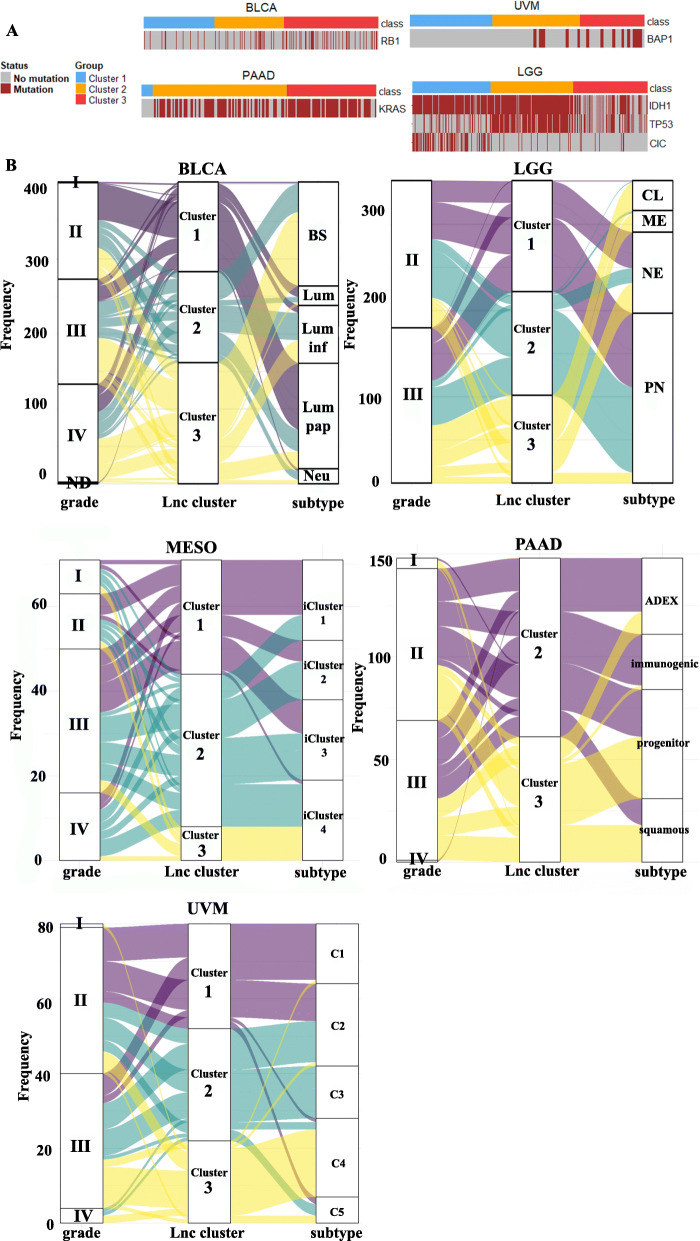
Associations of genomic mutations and molecular subtypes in glycolysis-associated long noncoding (lnc)RNA-classified clusters. **a** A heatmap showing gene candidates with nonsilent mutations enriched in certain clusters of bladder carcinoma (BLCA), pancreatic ductal adenocarcinomas (PAADs), uveal melanomas (UVMs), and low-grade gliomas (LGGs). **b** Alluvial plots demonstrating comparisons of lncRNA-stratified subgroups (lnc cluster) with tumor grade (grade) and molecular subtypes (subtype) in BLCA, LGG, mesotheliomas (MESO), PAAD, and UVM. ND, not determined; BS, basal/squamous; LUM, luminal-like; Lum-inf, luminal infiltrated; Lum pap, luminal papillary; Neu, neuroendocrine-like; PN, proneural; NE, neural; CL, classical; ME, mesenchymal

Next, we investigated molecular differences in distinct cancers between patient subgroups classified by glycolysis-associated lncRNA signatures. By comparing genomic mutations within these patients, we determined that several gene mutations were associated with different lncRNA-associated groups (Fig. 2a). RB1, KRAS, and BAP1 mutations were enriched in cluster 3 patients with BLCA, PAADs, and UVMs, respectively. As for LGGs, cluster 3 patients generally belonged to the IDH1 wild-type group and had fewer TP53 and CIC mutations.

Previous studies have categorized TCGA BLCA, LGG, PAAD, MESO, and UVM patients into different molecular types based on multiomic profiles [18–22]. Here we compared these defined subgroups and tumor grades with our classified groups by using glycolysis-correlated lncRNA signatures (Fig. 2b). Cluster 3 patients with LGG, BLCA, PAAD, or UVM exhibited higher tumor grades. As for molecular subtypes, cluster 3 patients with BLCA mainly belonged to the basal/squamous subtype, which had the poorest prognosis and exhibited a highly immune-infiltrated characteristic. Meanwhile, cluster 1 BLCA patients were mainly categorized into the luminal papillary type, which had more favorable prognoses. Similarly, in LGG patients, the mesenchymal subtype tended to have poor survival, highly infiltrative immune cells, and EMT activation, and the subtype was mainly enriched in the cluster 3 group. By contrast, patients in cluster 1 were enriched in the proneural and neural subtypes. In PAAD patients, cluster 3 patients were mainly enriched in squama cell subtypes that possessed activated hypoxia, inflammatory response, TGF-β signaling, and MYC pathway activation. For cluster 1 patients, all their histological types belonged to neuroendocrine, which was excluded in the study that defined these molecular subtypes [20]. As for MESO and UVM, no names were used to define their molecular types based on biological features. However, the cluster 4 patients of both UVM and MESO that had the poorest prognosis in previous findings [21, 22] were highly overlapped with cluster 3 patients in the present findings.

### EMT and inflammation-regulated pathways exhibit distinct patterns in glycolysis-associated lncRNA-classified groups

**Fig. 3:**
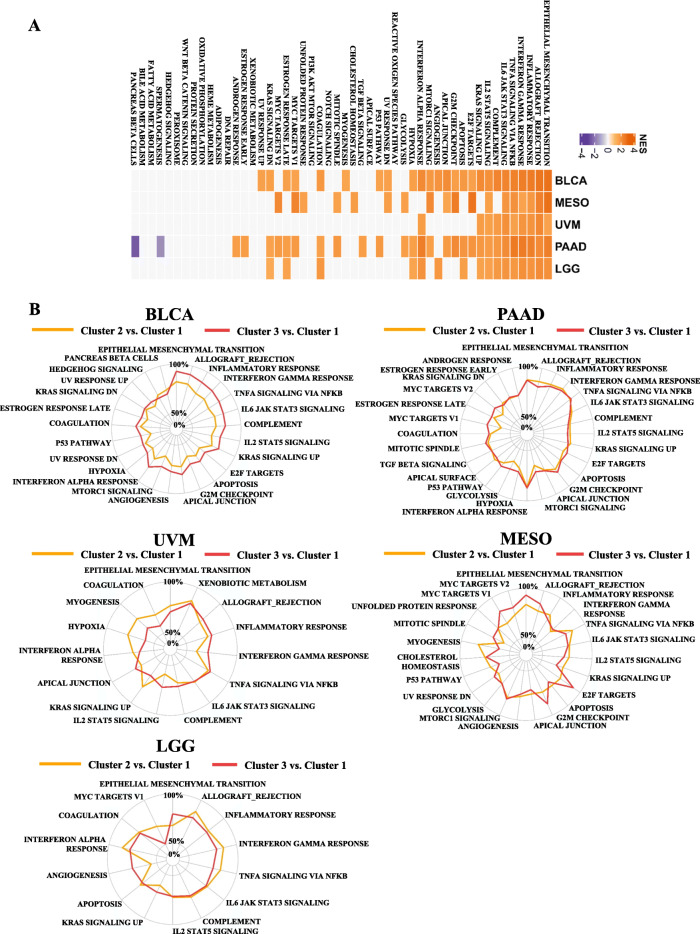
Varieties of signaling pathways in distinct clusters stratified by glycolysis-associated long noncoding (lnc)RNAs. **a** A heatmap demonstrating normalized enrichment scores (NESs) of Hallmark pathways calculated by comparing cluster 3 with cluster 1 (with a false discovery rate (FDR) of < 0.01). **b** Radar plots indicating NESs of Hallmark pathways calculated through a gene set enrichment analysis (GSEA) of cluster 3 versus cluster 1 and of cluster 2 versus cluster 1

To delineate underlying signal pathways associated with glycolysis-related lncRNAs, we conducted a DEG analysis within different clusters (Additional file 10: Figure S5). Signal pathways that were upregulated in the poorest prognosis group with the highest glycolysis scores included EMT, inflammatory responses, and interferon gamma signaling (Fig. 3a, b). Therefore, we inferred that lncRNA-based cluster 3 patients exhibited an upregulated status in the EMT and immune regulatory pathways; these lncRNAs might play critical roles in linking glycolytic signaling, EMT, and immune-suppressive microenvironments.

### Patients with poor prognoses as classified by glycolytic-associated lncRNAs exhibit a highly infiltrative immune microenvironment

**Fig. 4:**
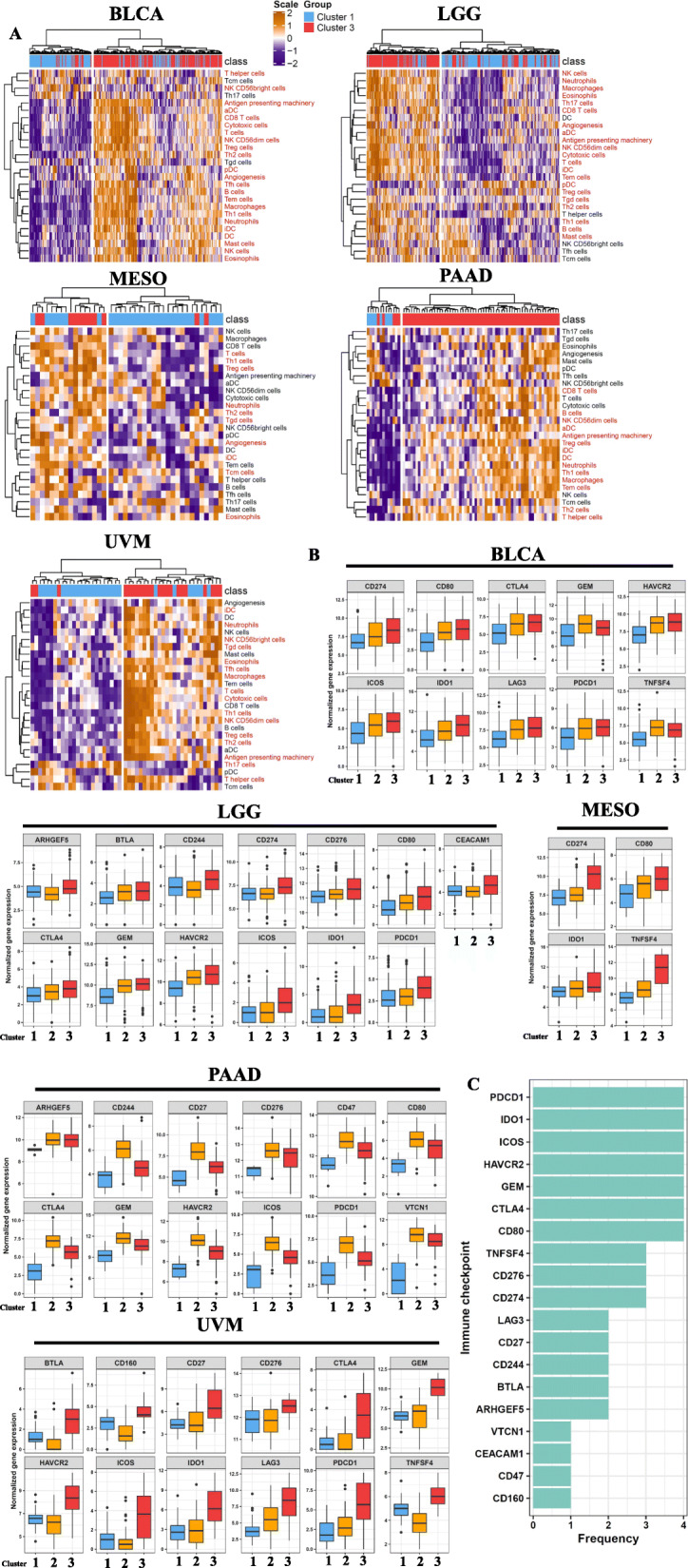
Immune-related signaling is enriched in glycolysis-associated long noncoding (lnc)RNA-stratified cluster 3 cancer patients. **a** Unsupervised clustering of distinct immune cell infiltrations in glycolysis-stratified cluster 3 and cluster 1 cancer patients. **b** A boxplot demonstrating immune checkpoints that were upregulated in cluster 3 (with an effect size of > 0.5 and *p* value of < 0.001) compared with cluster 1 or cluster 2. **c** Bar plot demonstrating frequencies of immune checkpoints upregulated in cluster 3 cancer patients across the five cancer types. The *y*-axis indicates the names of immune checkpoints, and the *x*-axis represents the number of cancer types

To further clarify differences in the immune microenvironments of cluster 1 and cluster 3 patients, we inferred immune cell infiltration scores in the five cancer types by using the expression levels of gene markers in immune cells. We then conducted unsupervised hierarchical clustering to categorize patients into high and low immune infiltration groups (Fig. 4a). We determined that most cluster 3 patients belonged to a high immune infiltration group. By contrast, cluster 1 patients mainly belonged to a low immune infiltration group. Despite the high infiltration of immune cells in cluster 3 patients, they still had the poorest prognosis. Hence, we speculated that cluster 3 cancer patients might have upregulated immune-suppressive checkpoints that facilitated cancer cells to evade attack by immune cells. Therefore, we compared the expression levels of known immune-suppressive checkpoints in different clusters (Fig. 4b). The results revealed that PDCD1, IDO1, and CTLA4 were significantly upregulated in cluster 3 patients compared with cluster 1 patients in multiple cancer types (Fig. 4c). Taken together, these results suggest that glycolysis-associated lncRNAs can be used to identify a subgroup of cancer patients with the poorest prognosis. They also suggest that lncRNAs activate oncogenic pathways including hypoxia and EMT and promote a highly infiltrative immune tumor environment. Moreover, these cancer subsets may be vulnerable to treatment with immunotherapies due to their enriched immune targets.

### Characterization of glycolysis-associated lncRNAs in cancers

**Fig. 5:**
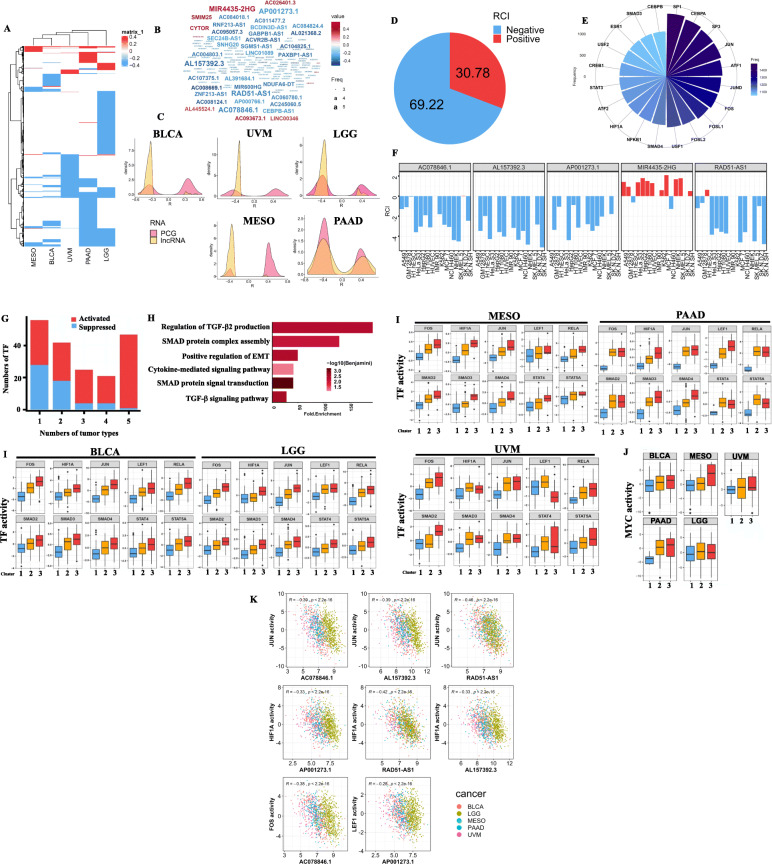
Characterization of glycolysis-related long noncoding (lnc)RNAs in five cancer types. **a** A heatmap demonstrating glycolysis-associated lncRNAs in five cancer types. Colors in the heatmap indicate the degree of correlation between glycolysis scores and lncRNA expressions. **b** Word cloud of the glycolysis-correlated lncRNAs. Number of cancer types is associated with the text size of the lncRNA names, whereas the color of the lncRNA indicates median correlation coefficients. **c** Density curve showing correlation coefficients of genes (pink) and lncRNAs (yellow) significantly associated with glycolysis scores. Distributions of genes and lncRNAs were compared using the Kolmogorov–Smirnov test. The *x*-axis represents correlation coefficients with glycolysis scores between genes and lncRNAs, and the *y*-axis represents numbers of lncRNAs and genes. **d** Pie chart demonstrating percentages of lncRNAs with positive or negative values in the relative concentration index (RCI). **e** A circular bar plot indicating frequencies of transcription factors (TFs) negatively associated with glycolysis-related nuclear lncRNAs. **f** Bar plots demonstrating cellular localizations of five lncRNAs significantly correlated with glycolysis scores across five cancer types. **g** A bar plot showing numbers of activated and suppressed TFs in cluster 3 compared with cluster 1 in five cancer types. The *x*-axis represents the number of cancer types, and the *y*-axis represents the number of TFs. **h** Functional annotation analyses of activated TFs involved in more than four cancer types in cluster 3 cancer patients. The *y*-axis represents significant enriched pathways (with an FDR of < 0.01), and the *x*-axis represents multiples of enrichment of indicated signal pathways. **i** A boxplot indicating TFs that were upregulated in cluster 3 compared with cluster 1. The *y*-axis represents transcriptional activity inferred by target gene expression levels by using analytic rank-based enrichment analyses, and the *x*-axis represents different clusters classified by glycolysis-associated lncRNAs. **j** MYC activity in five cancer types. **k** Dot plots of lncRNA-TF pairs that exhibit negative correlations across five cancer types. The association between TF and lncRNA within each cancer type exhibits a correlation coefficient of <− 0.3 and a *p* value of < 0.001. The pooled *p* value and correlation coefficient are indicated in each dot plot

Studies have indicated that lncRNAs exhibit tissue-specific expression patterns [23, 24]. Here we found that glycolysis-associated lncRNAs also followed this rule. Most of the lncRNAs were correlated with glycolytic activity in only one cancer type (Fig. 5a). However, common candidates were still associated with glycolytic activity across the five cancers, namely MIR4435-2HG, AC078846.1, AL157392.3, AP001273.1, and RAD51-AS1 (Fig. 5b). The results implied that these five lncRNAs participate in glycolytic signaling in several cancers. Furthermore, most of the glycolysis-associated lncRNAs exhibited a negative correlation with glycolytic activity compared with protein coding genes (Fig. 5c). Because the functionalities of lncRNAs differ based on their location in the cellular compartment, we utilized the LncATLAS database to investigate the localization of these glycolysis-associated lncRNAs. We determined that 69.22% of the RCIs were negative, which suggests that nearly 70% of lncRNAs were located in nuclei (Fig. 5d). One group of TF activities was highly negatively correlated with glycolysis-associated lncRNAs in nuclei (Fig. 5e). As a previous study indicated [25], nuclear lncRNAs function in chromatin organization, transcriptional and post-transcriptional gene expression regulation, and direct interactions with TFs to facilitate gene expressions.

We then investigated associations between glycolysis-associated lncRNAs and dysregulated TFs among the five cancer types. Despite these cancers having distinct lncRNA patterns correlated with glycolytic activity, they shared common pathways and upregulated TFs in cluster 3 cancer patients. Next, we focused on nuclear lncRNAs that had distinct expressions in cluster 3 patients of the five cancer types. Four lncRNA candidates were selected; MIR4435-2HG was excluded because it was distributed mainly in the cytoplasm (Fig. 5f). Next, we investigated differences in TF activity in glycolysis activity-associated clusters. After conducting an aREA, we inferred TF activity by examining the expressions of TF downstream targets. Then, we identified putative upstream regulators that might be responsible for the poor prognosis of cluster 3 cancer patients (Fig. 5g). The results indicated that 47 TF candidates were dysregulated in the five cancers. Functional annotation analyses of these TFs indicated that most of them were enriched in mothers against decapentaplegic homolog (SMAD) protein complex assembly, cytokine-mediated signal pathways, and the transforming growth factor (TGF)-β signal pathway (Fig. 5h). These results further implied the potential roles of glycolysis-associated lncRNAs in cancer immune regulation and EMT activation. Prominent TFs involved in these pathways were HIF1A, FOS, JUN, the SMAD family, and the STAT family (Fig. 5i). In addition, the oncogenic TF, MYC, has been identified as a key regulator of glycolysis. Therefore, we also investigated the potential link between glycolysis-associated lncRNA-stratified clusters and MYC regulation. In pathway analyses (Fig. 3a), MYC targets were significantly activated in PAAD and MESO. In TF analyses, we observed a moderate upregulation of MYC activity in the highly glycolytic groups for BLCA, LGG, and UVM and a significant upregulation in PAAD and MESO (Fig. 5j, Additional file 11: Figure S6). The results indicated that these TFs—HIF1A, JUN, LEF1, and FOS—were negatively correlated with four glycolysis-associated lncRNAs across the five cancer types (Fig. 5k), suggesting that the low expression of these lncRNA signatures was accompanied by glycolytic signaling and oncogenic TF activation. Based on the functional annotation analyses of activated TFs, we determined that activated TGF-β-mediating SMAD signaling pathways can be used to distinguish these three subtypes. Furthermore, TGF-β-mediating SMAD signaling pathways have been identified to be involved in regulating EMT [26] and immune infiltration [27]. Therefore, we identified these three subtypes as cluster 1 (low TGF-β/SMAD), cluster 2 (median TGF-β/SMAD), and cluster 3 (high TGF-β/SMAD).

### MIR4435-2HG interlinks glycolysis and its downstream associated genes in cancers

**Fig. 6:**
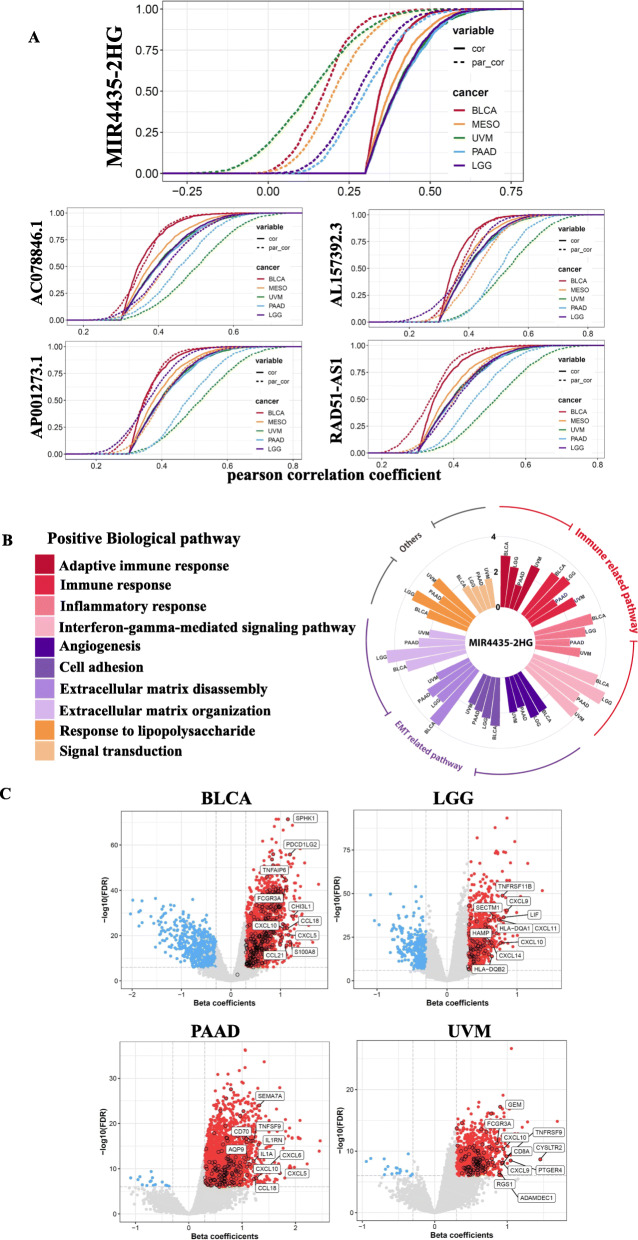
MIR4435-2HG links glycolysis and its related signal pathways including the epithelial-to-mesenchymal transition (EMT) and immune regulation. **a** Cumulative distribution curve (CDF) of glycolysis-gene correlations with or without adjustment for MIR4453-2HG by using a first-order partial correlation. Solid lines indicate CDFs of correlation coefficients between glycolysis scores and gene expressions without adjustment. Dashed lines indicate first-order partial correlation-adjusted relations between glycolysis scores and gene expressions. These two distributions were compared using the Kolmogorov–Smirnov test. The *x*-axis represents Pearson correlation coefficients between glycolysis scores and gene expressions, and the *y*-axis represents cumulative probabilities. **b** A circular bar plot displaying MIR4435-2HG-associated signal pathways. Genes significantly associated with MIR4435-2HG but independent of the methylation status and copy number alterations were identified using a multivariate linear regression. Pathways enriched in MIR4435-2HG-associated genes are shown in the circular bar plot. The height of the bars indicates the degree of enrichment. Only pathways with a false detection rate (FDR) of < 0.01 are included in the plot. **c** A volcano plot indicating MIR4453-2HG-associated genes that were independent of copy number alterations and the DNA methylation status. The *x*-axis represents multivariate linear regression-adjusted coefficients between MIR-4435-2HG and glycolysis scores. Gene candidates involved in immune-related signal pathways are labeled with a black round line, and their names are indicated in the plots

Finally, we investigated which lncRNAs might potentially be involved in the coexpression of glycolysis-related genes. We performed a first-order partial correlation to adjust correlation coefficients between glycolysis scores and positively associated genes. We observed a significant alteration in correlation coefficients in these glycolysis-associated genes after adjusting for the effect of mutually associated lncRNAs (Fig. 6a). In particular, when the effects of MIR4435-2HG are removed, correlations between glycolysis scores and associated genes significantly decreased in the five cancer types. This suggests that MIR4435-2HG plays a crucial role in linking glycolysis and related signal pathways. Next, we performed a multivariate linear regression to identify MIR4435-2HG-associated genes by adjusting for other covariates, including copy number alterations and DNA methylation (Fig. 6b). MIR4435-2HG-associated genes were linked to EMT and immune signaling across the four cancer types of BLCA, LGG, UVM, and PAAD (Fig. 6c). By contrast, no significantly associated genes were identified in MESO. In addition, we observed that MIR4435-2HG expression was positively associated with MYC activity (which was reported to play prominent roles in glycolysis regulation) across the five cancer types (Additional file 12: Figure S7). Among the top 10 MIR4435-2HG-associated gene candidates in immune signal pathways, cytokines were the most common. In particular, C-X-C motif chemokine 10, a well-established chemotactic cytokine, was strongly correlated with MIR4435-2HG expression in these four cancer types. Consequently, we concluded that lncRNAs, especially MIR4435-2HG, play key roles in linking glycolytic pathways with other oncogenic signal pathways, including EMT and immune regulation, in some cancer types. Besides, all the high-resolution images of results were showed as Additional file 13: Figure S8.

## Discussion

Glycolysis, a metabolic pathway, supplies energy, promoting cancer malignancy. Although the evidence of a crosstalk between glycolysis and lncRNA regulation in cancer progression is mounting [28, 29], a clinical link connecting lncRNAs and glycolysis is not fully understood. We conducted a pan-cancer scale analysis to identify glycolysis-associated lncRNAs in 33 cancer types. We determined that the prognoses of five cancer types, BLCA, PAAD, LGG, MESO, and UVM, were significantly correlated with glycolysis-associated lncRNA signatures. Furthermore, we comprehensively analyzed changes in gene mutations, molecular subtypes, TFs, oncogenic signal pathways, and immune cell infiltration in patients stratified by glycolysis-associated lncRNAs. Finally, we identified five lncRNAs, namely MIR4435-2HG, AC078846.1, AL157392.3, AP001273.1, and RAD51-AS1, which exhibited significant correlations with glycolysis across the five cancer types. In particular, MIR4435-2HG was suggested to play a critical role in connecting glycolysis, EMT, and immune cell infiltration in the cancers.

For the glycolysis evaluation of each cancer patient, we chose the ssGSEA-derived glycolysis score instead of the direct correlations of glycolysis-associated genes because of the large number of glycolysis-involved genes in this methodology (200 gene candidates). Directly correlating lncRNAs with these genes would generate hundreds of correlation results and *p* values, making evaluating the degree of association of each lncRNA with glycolysis activation difficult. Therefore, an ssGSEA algorithm was utilized to treat the 200 glycolysis-involved candidates as a gene set to perform a GSEA for individual patients. Through this method, we were able to compress the genes into a single score for each patient and capture the degree of glycolysis activation. We did not directly use the target genes of lncRNAs due to the complexity of the regulatory mechanisms of lncRNAs. The functions of lncRNAs in post-transcriptional regulation include the microRNA sponge, interaction with the chromatin modulator, and direct targeting of downstream RNA. In addition, only focusing on glycolysis-involved genes that belong to lncRNA direct targets would risk our overlooking other promising candidates. Furthermore, no bioinformatics tools that can accurately predict lncRNA target genes are yet available. Therefore, ssGSEA-derived glycolysis scoring may be a suitable tool for identifying glycolysis-associated lncRNAs.

Relationships between glycolysis and the immune resistance of cancers have recently been reported. Tumor cells utilize glucose and metabolically compete with T cells through impairing mammalian target of rapamycin (mTOR) activity and glycolytic activity in T cells, leading to the overriding of the capability of T cell-mediated cytotoxicity [30]. In addition, highly activated glucose metabolism in cancer cells promotes lactate accumulation in the TME [31]. This extracellularly accumulating lactate blocks lactate export from T cells, leading to the generation of dysfunctional aerobic glycolysis, a crucial mechanism for maintaining T cell effector function. In addition to immune regulation, metabolic reprogramming (including glycolysis) with highly invasive and drug resistance features was reported to possess the ability to transform cancer cell phenotypes toward EMT [32]. The EMT process has also identified to be linked to immune evasion by cancer cells [33, 34]. However, relationships among glycolysis, EMT, and immune regulation in cancers are still not fully understood. In our analyses, we determined that EMT and inflammatory responses, including immune-suppressive ligand expression, were enriched in cluster 3 cancer patients, as classified by glycolysis-associated lncRNAs. These findings suggest that the TME of cluster 3 cancer patients is surrounded with different types of immune cells, and the upregulated immune-suppressive ligands and EMT activation protect cancer cells from attack by immune cells. Therefore, the “hot” immune microenvironment in cluster 3 cancer patients implies that immune checkpoint blockade therapy might be suitable.

In our genomic mutation analysis, we identified specific gene mutations that were associated with glycolysis-associated lncRNA-stratified clusters in different cancers. Some of these genes have been reported to be involved in glucose metabolism. For example, in mice bearing RB1 null lung cancer [35], upregulation of glucose transporter (GLUT) 1 and two rate-limiting enzymes in glycolysis, hexokinase-2 (HK2) and pyruvate kinase isozymes 2, was observed, suggesting that RB1 regulates glucose metabolism. In the present study, the cluster with high glycolytic activity mainly exhibited RB1 mutation in BLCA. Although loss of RB1 may serve as a poor prognosis predictor in BLCA [36], the relationship between RB1 and glycolysis in BLCA remains unclear. Additionally, in PAAD and UVM, we identified KRAS and BAP1 mutations, respectively, as enriched in clusters with high glycolytic activity. In PAAD, KRAS mutation activates glycolytic signaling mainly through MEK activation and Myc-dependent transcription, resulting in the upregulation of GLUTs and rate-limiting enzymes of glycolysis, such as HK2, phosphofructokinase-1, and lactate dehydrogenase A (LDHA) [37]. A multi*-*omics analysis integrating transcriptome, metabolite, and genomic analysis revealed that UVM cells with BAP1 mutation maintain their energy demand through oxidative phosphorylation and glycolytic pathway [38]. Additionally, the distinct metabolic features of mutant BAP1 and wild-type UVM further lead to different responses to metabolic inhibitors. These findings indicate that KRAS and BAP1 mutations respectively drive metabolic alterations in PAAD and UVM. In gliomas, the cluster with high glycolytic activity mainly belongs to IDH1 wild-type cancer patients, as per our findings. A previous study reported that glioma cells with IDH1 mutation produce 2-hydroxyglutarate, which promotes HIF-1α degradation, accompanied by the downregulation of glycolysis-related genes including *SLC2A1*, *PDK1*, *LDHA*, and *SLC16A3* [39]. By contrast, IDH1 wild-type gliomas exhibited an activated glycolysis pathway. Taken together, our lncRNA-stratified clusters not only exhibit different glycolytic activities but also distinct genomic mutations that have been reported to be involved in glycolysis signaling. However, whether these genomic mutations might be the drivers altering glycolysis-associated lncRNA profiles requires further investigation.

In our TF analysis, we determined that activated prominent TFs in highly glycolytic clusters across BLCA, LGG, PAAD, and UVM were mainly involved in TGF-β signaling and SMAD protein complex assembly. SMAD proteins are downstream signal transducers of the TGF-β signaling pathway, which functions as an immune-suppressive regulator in cancers. For instance, Smad3-mediated TGF-β signaling was reported to suppress the cytotoxic activity of NK cells by blocking the production of CD16-mediated IFN-gamma [40]. Another SMAD protein, SMAD4, has dual roles in regulating NK immunity in a context-specific manner [41]. In the initial phase of tumor formation, SMAD4 has a mainly positive effect in promoting the development and antitumor activity of NK cells. However, at the late stage of cancer development, NK cells are surrounded with TGF-β produced by tumor cells. In this condition, SMAD4 cooperates with p-SMAD2 and p-SMAD3 to suppress NK cell-mediated cytotoxicity. Our analyses revealed that the group with high glycolytic activity demonstrated a microenvironment with highly infiltrated NK cells. Furthermore, coordinated upregulation of SMAD2, SMAD3, and SMAD4 was also observed in highly glycolytic patients. Taken together, these results imply that highly glycolytic tumor cells might impede NK-mediated immunity through SMAD signaling, and such tumor cells might be vulnerable to SMAD4 inhibition. However, the relationship between glycolysis-related lncRNAs and SMAD complex regulatory mechanisms in tumor cells needs to be further validated.

Among the glycolysis-associated lncRNAs we identified, some lncRNAs have been reported to be directly involved in glycolysis signaling. For example, plasmacytoma variant translocation 1, which exhibited positive associations with glycolysis scores in six of thirty-three cancer types in our findings, was suggested to function as a microRNA sponge in suppressing miR-497 expression, leading to the promotion of HK2 upregulation and osteosarcoma progression [42]. Nuclear factor (NF)-κB-interacting lncRNA (NKILA), which was positively correlated with glycolytic activity in the five cancer types, activates hypoxia-inducible factor 1α expression to promote the hypoxia-mediated Warburg effect on gliomas [43]. Some lncRNA candidates in our findings might also be implicated in regulating glycolysis. For instance, cytoskeleton regulator RNA was reported to interact with Sam68, an RNA-binding protein that possesses an oncogenic function in cancers [44]. Furthermore, Sam68 can upregulate the expression of pyruvate kinase isozymes M2 (PKM2), a key enzyme for glycolysis-dominated energy metabolism [45], through facilitating the transport of PKM2 messenger (m)RNA from the nucleus to cytoplasm [46]. MIR4435-2HG, an lncRNA positively correlated with glycolysis in 20 cancer types, enhances YAP expression for cancer progression [47]. Furthermore, YAP is recognized as a TF and is responsible for upregulating the expression of glucose metabolism enzymes such as HK2 and 6-phosphofructo-2-kinase/fructose-2,6 biphosphatase 3 [48]. However, their roles in the glycolysis process require further investigation.

Among the five lncRNAs that were consistently correlated with glycolysis scores across different cancers in our findings, MIR4435-2HG was identified as being highly associated with glycolysis and its correlated genes. By performing a multivariate linear regression adjustment, we also uncovered that immune and EMT-involved genes are positively correlated with MIR4435-HG expression. Several studies have demonstrated the oncogenic roles of MIR4435-2HG in cancer processes. MIR4435-2HG promotes gastric cancer cell migration and proliferation through Wnt/β-catenin signaling [49]. The upregulation of MIR4435-2HG promotes oral squamous carcinoma cell proliferation through inducing TGF-β1 upregulation [50]. Nevertheless, few studies have indicated links among MIR4435-2HG, glycolysis, and immune regulation in cancers. Our analyses suggest that MIR4435-2HG participates in interrelations among glycolysis, immune resistance, and EMT. The present study has limitations. The lack of public available RNA sequencing data limited us to validate the classification effectiveness of our genome classifier which is predominantly based on the glycolysis-associated lncRNAs or the gene candidates. More clinical data of individuals with other types of cancer or from large cohort studies are required for validation. Several lncRNAs in our findings have rarely been reported, especially those negatively correlated with glycolysis. These should be further investigated.

## Conclusions

We identified a subgroup of cancer patients stratified by glycolysis-correlated lncRNA signatures with the poorest prognosis, a highly infiltrative immune microenvironment, and EMT activation and thus provide novel aspects for cancer therapy.

## Supplementary Information

**Additional file 1: Table S1.** Glycolysis candidate genes in each cancer types.

**Additional file 2: Figures S1.** Consensus cumulative distribution function (CDF) and delta area for five cancer types.

**Additional file 3: Table S2.** DEGs in LGG. Table S3. DEGs in BLCA.

**Additional file 4: Table S4.** Gene candidates in TCGA BLCA data. Table S5. Ggene candidates in TCGA LGG data.

**Additional file 5. Table S6.** Number of tumor tissues from TCGA pan cancer data.

**Additional file 6: Figures S2.** Flowchart for filtering cancer types and selecting glycolysis-associated long non-coding (lnc) RNAs.

**Additional file 7: Figures S3.** Stratification of patients into different clusters by a consensus clustering analysis.

**Additional file 8: Table S7.** Glycolytic score associated lncRNA in UVM.

**Additional file 9: Figures S4.** Flow chart for establishing genomic classifiers.

**Additional file 10: Figures S5.** Flow chart for Transcription factor activity analyses.

**Additional file 11: Figures S6.** Negative correlation of lncRNA-MYC activity pairs.

**Additional file 12: Figures S7.** Positive correlation of lncRNA-MYC activity pairs.

**Additional file 13: Figures S8.** All the high resolution images in the result section.

## Data Availability

The datasets used and/or analyzed are available from the corresponding author on reasonable request.

## Abbreviations

aREAs: Analytical rank-based enrichment analyses
BLCA: Bladder carcinoma
DEG: Differentially expressed gene
EMT: Epithelial-to-mesenchymal transition
FDR: False discovery rate
GSEA: Gene set enrichment analysis
LGG: Low-grade gliomas
lncRNA: Long noncoding RNA
MESO: Mesotheliomas
NES: Normalized enrichment score
PAAD: Pancreatic ductal adenocarcinoma
RCI: Relative concentration index
RNA-Seq: RNA sequencing
ssGSEA: Single-sample gene set enrichment analysis
TCGA: The Cancer Genome Atlas
TF: Transcription factor
TME: Tumor microenvironment
UVM: Uveal melanomas
