# Supplementary material for: Glycolysis-associated lncRNAs identify a subgroup of cancer patients with poor prognoses and a high-infiltration immune microenvironment
Source: BMC Med. 2021 Feb 25;19:59. doi: 10.1186/s12916-021-01925-6 (PMC7905662; doi:10.1186/s12916-021-01925-6)

Supple. Fig. 1

BLCA

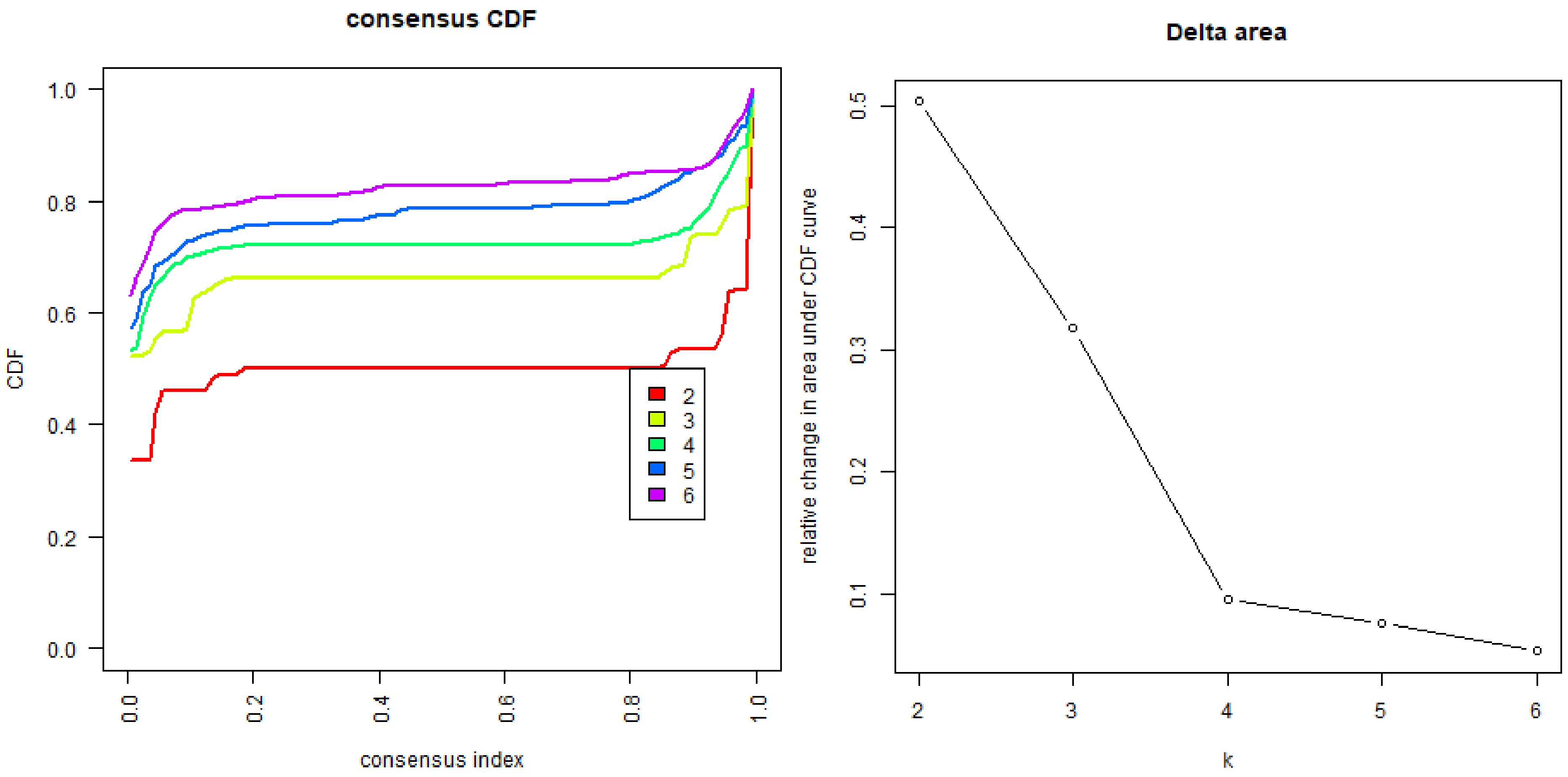

Supplemental Fig. 1. The consensus cumulative distribution function (CDF) and delta area for five cancer types.

Supple. Fig. 1

LGG

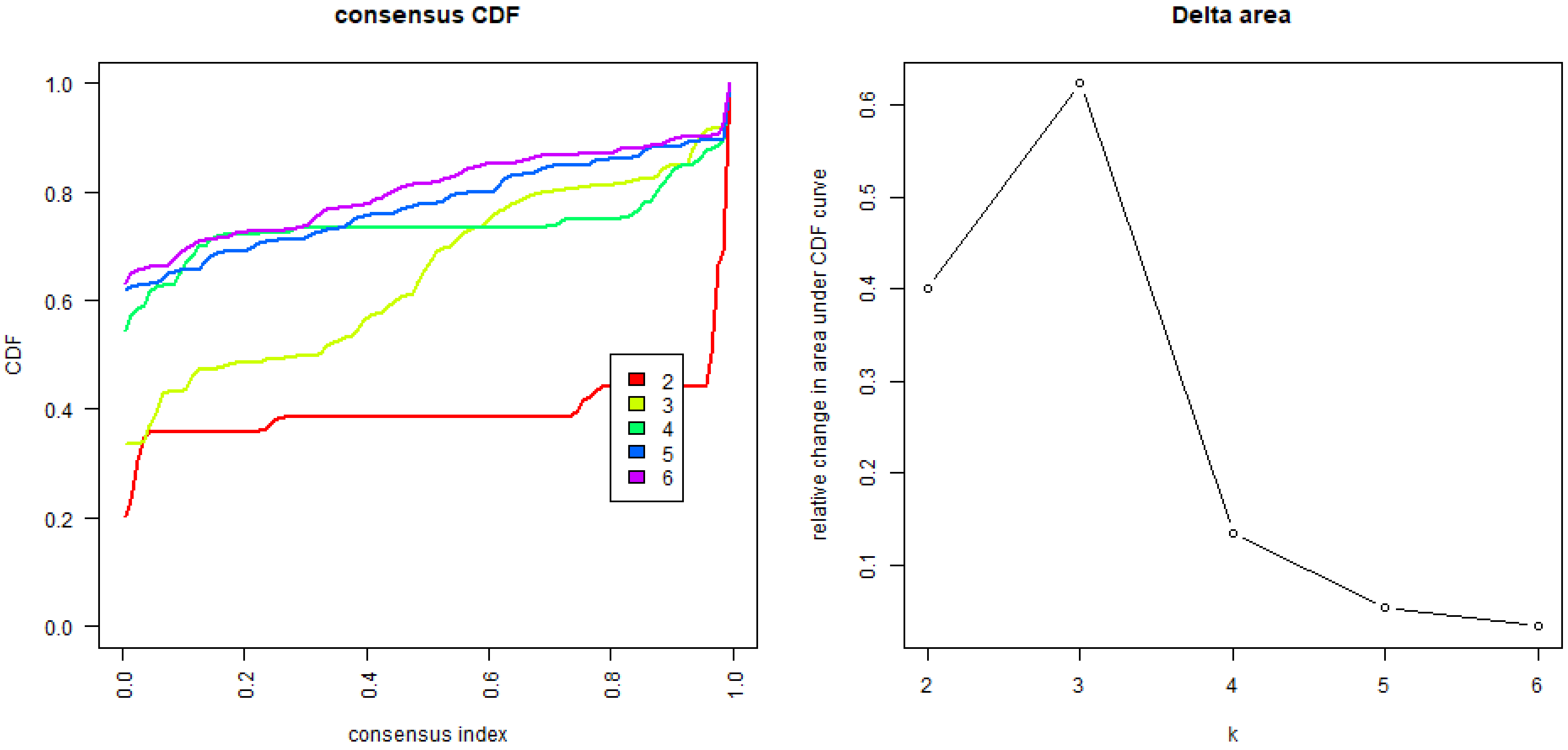

Supple. Fig. 1

MESO

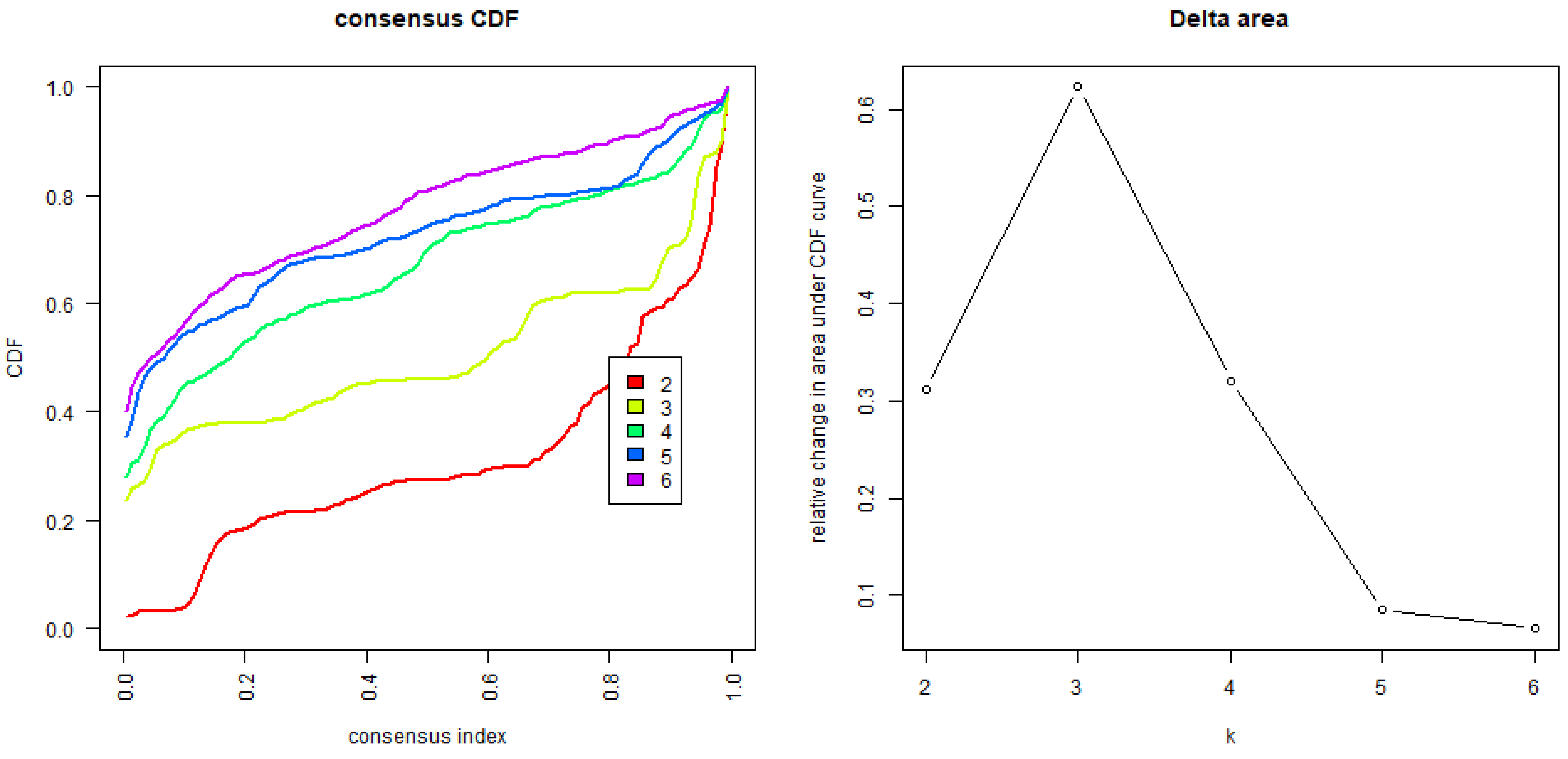

Supple. Fig. 1

PAAD

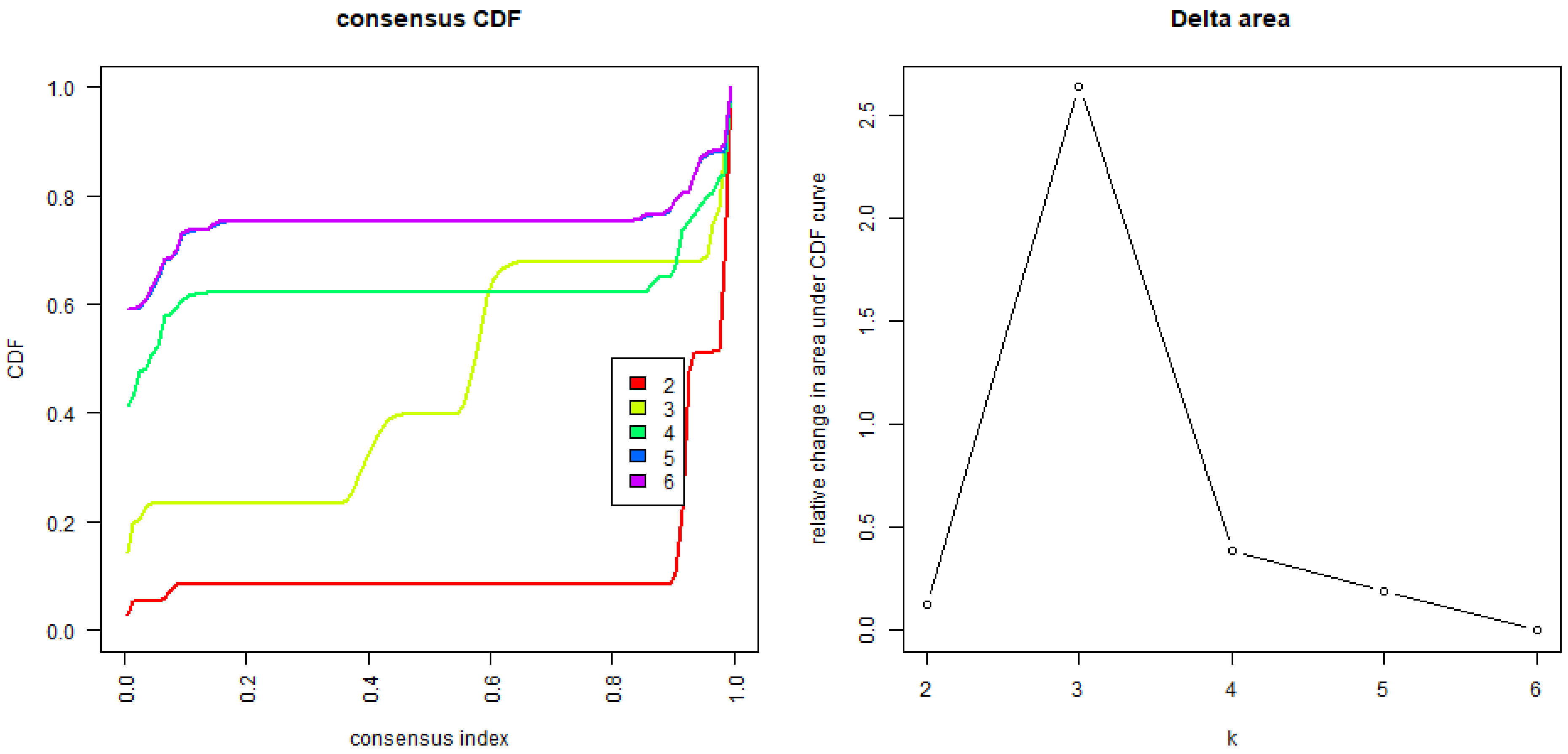

Supple. Fig. 1

UVM

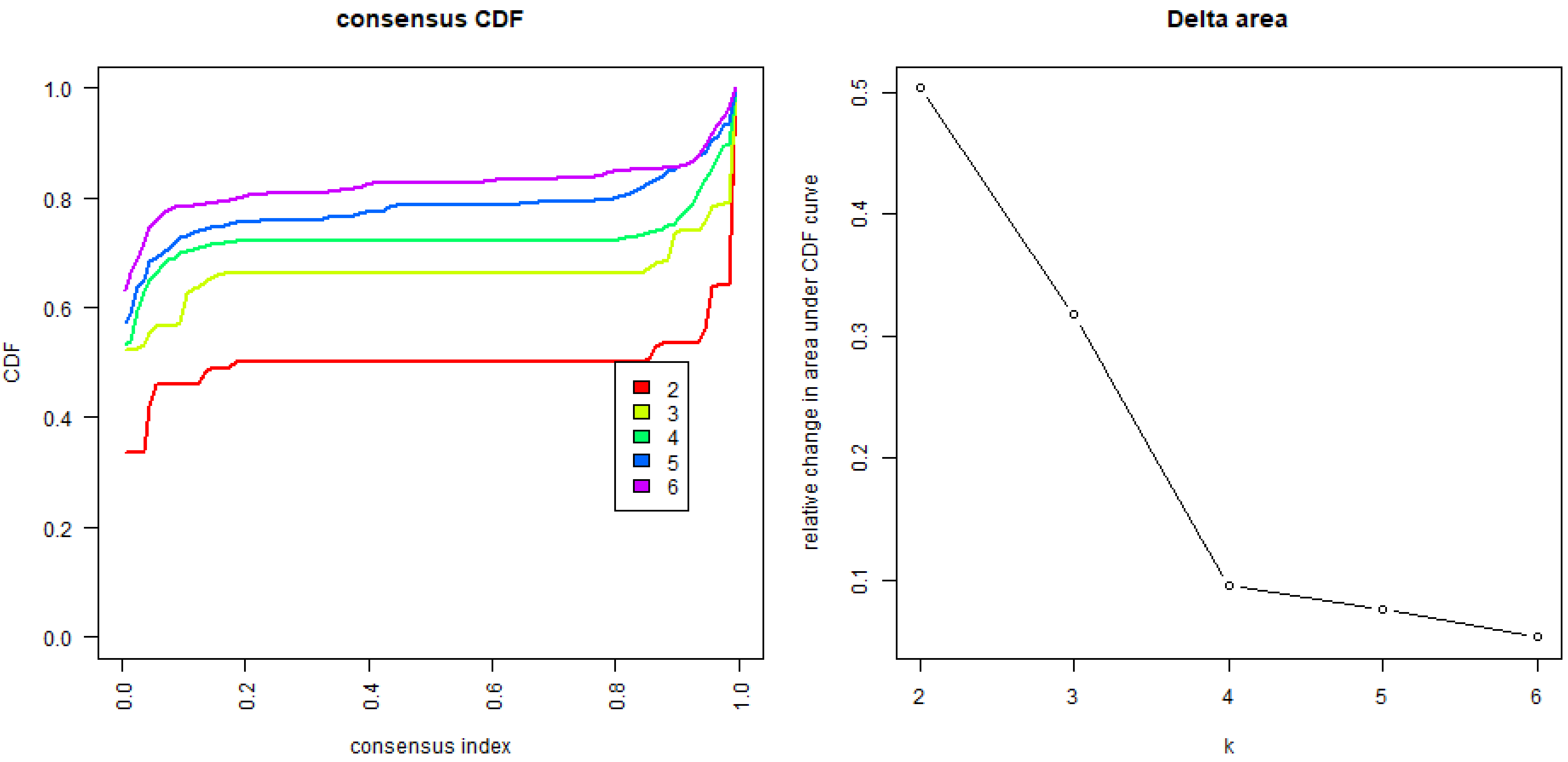

Supplement: Supplementary file 2 — Additional file 2: Figures S1. Consensus cumulative distribution function (CDF) and delta area for five cancer types. [file 12916_2021_1925_MOESM2_ESM.pdf]
