# Supplementary material for: Glycolysis-associated lncRNAs identify a subgroup of cancer patients with poor prognoses and a high-infiltration immune microenvironment
Source: BMC Med. 2021 Feb 25;19:59. doi: 10.1186/s12916-021-01925-6 (PMC7905662; doi:10.1186/s12916-021-01925-6)
Supplement: Supplementary file 6 — Additional file 6: Figures S2. Flowchart for filtering cancer types and selecting glycolysis-associated long non-coding (lnc) RNAs. [file 12916_2021_1925_MOESM6_ESM.pdf]

Supple. Fig. 2

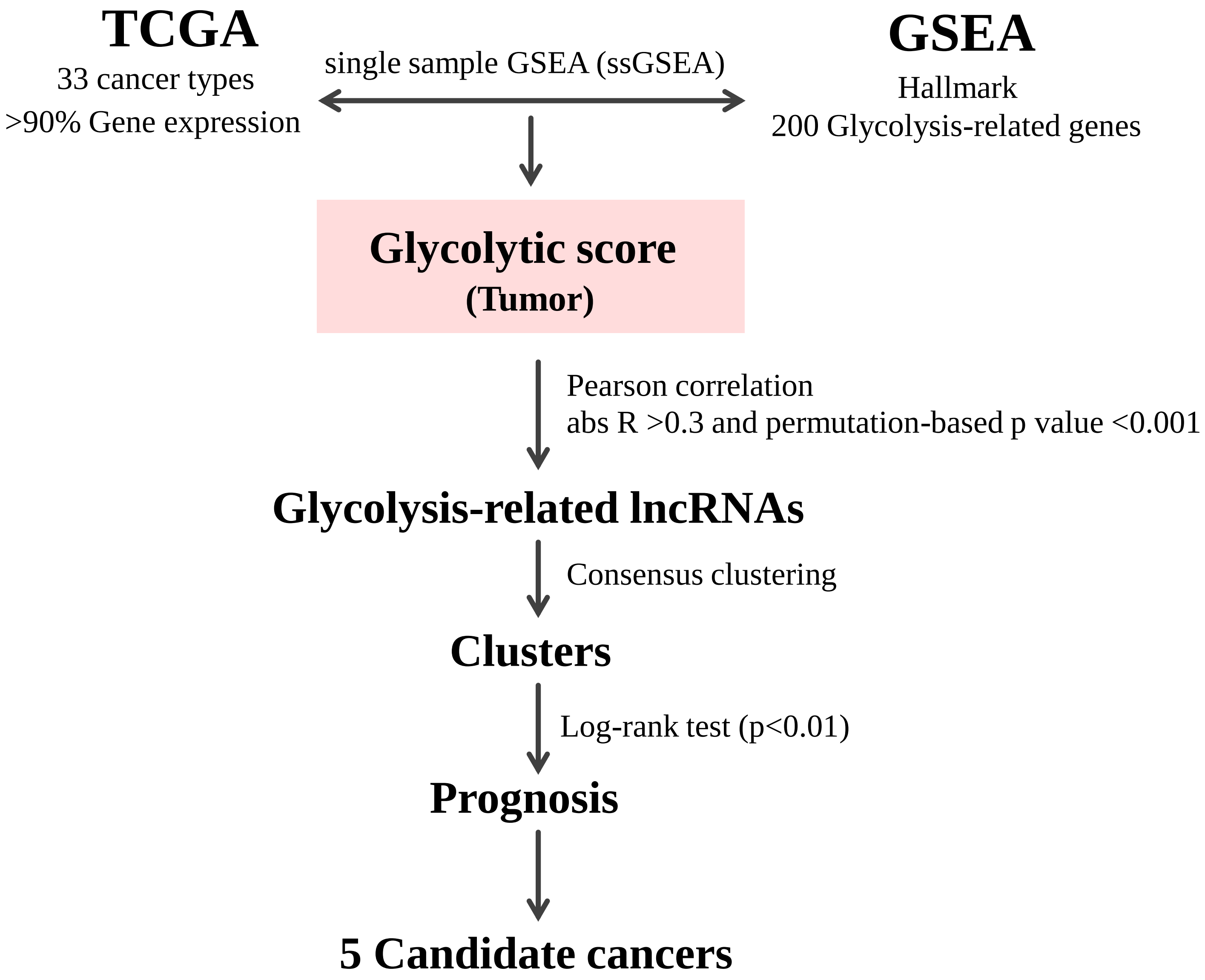

Supplemental Fig. 2. Flowchart for filtering cancer types and selecting glycolysis-associated long non-coding (lnc)RNAs in the present study.
