# Supplementary material for: Glycolysis-associated lncRNAs identify a subgroup of cancer patients with poor prognoses and a high-infiltration immune microenvironment
Source: BMC Med. 2021 Feb 25;19:59. doi: 10.1186/s12916-021-01925-6 (PMC7905662; doi:10.1186/s12916-021-01925-6)

Supple. Fig. 3

BLCA

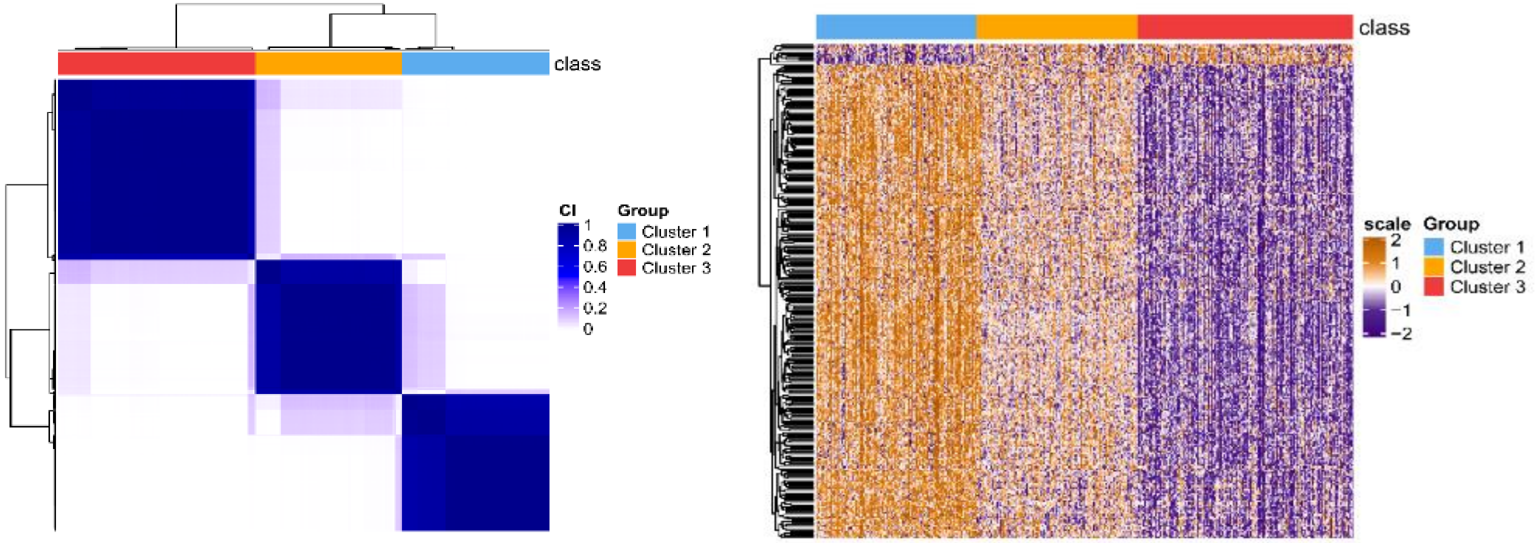

Supplemental Fig. 3. Stratification of patients into different clusters by a consensus clustering analysis. The right panel shows unsupervised clustering results of glycolysis-associated long non-coding (lnc)RNAs in bladder carcinoma (BLCA), low-grade gliomas (LGGs), mesotheliomas (MESOs), pancreatic ductal adenocarcinomas (PAADs), and uveal melanomas (UVMs).

Continued Supple. Fig. 2

LGG

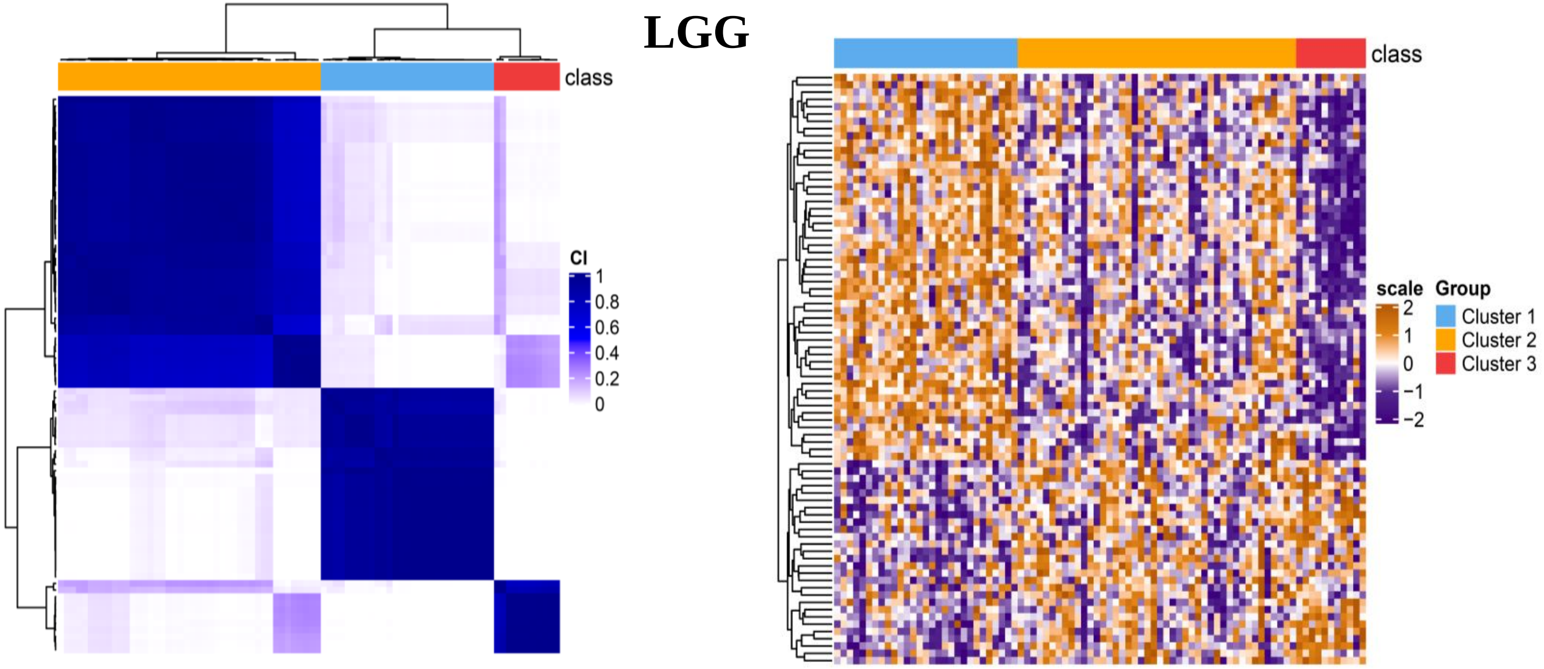

MESO

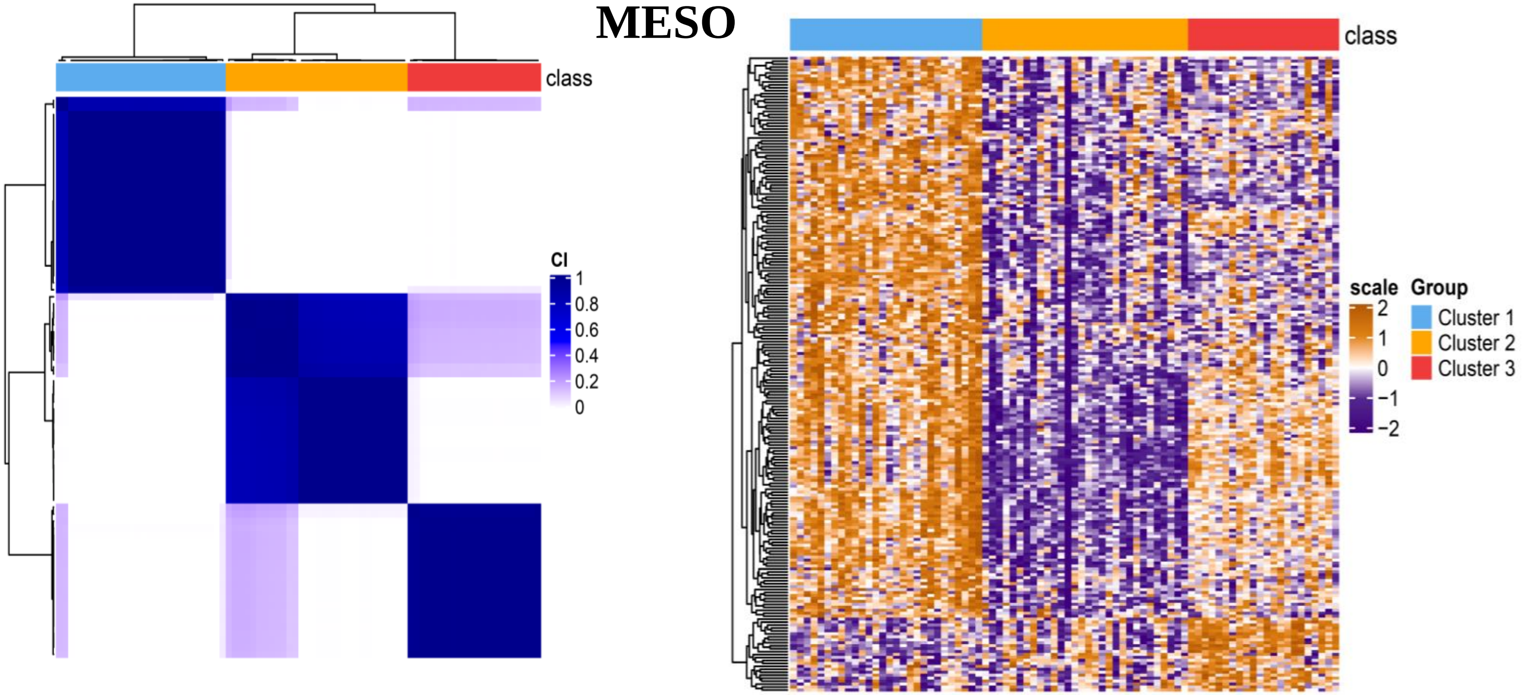

Continued Supple. Fig. 2

UVM

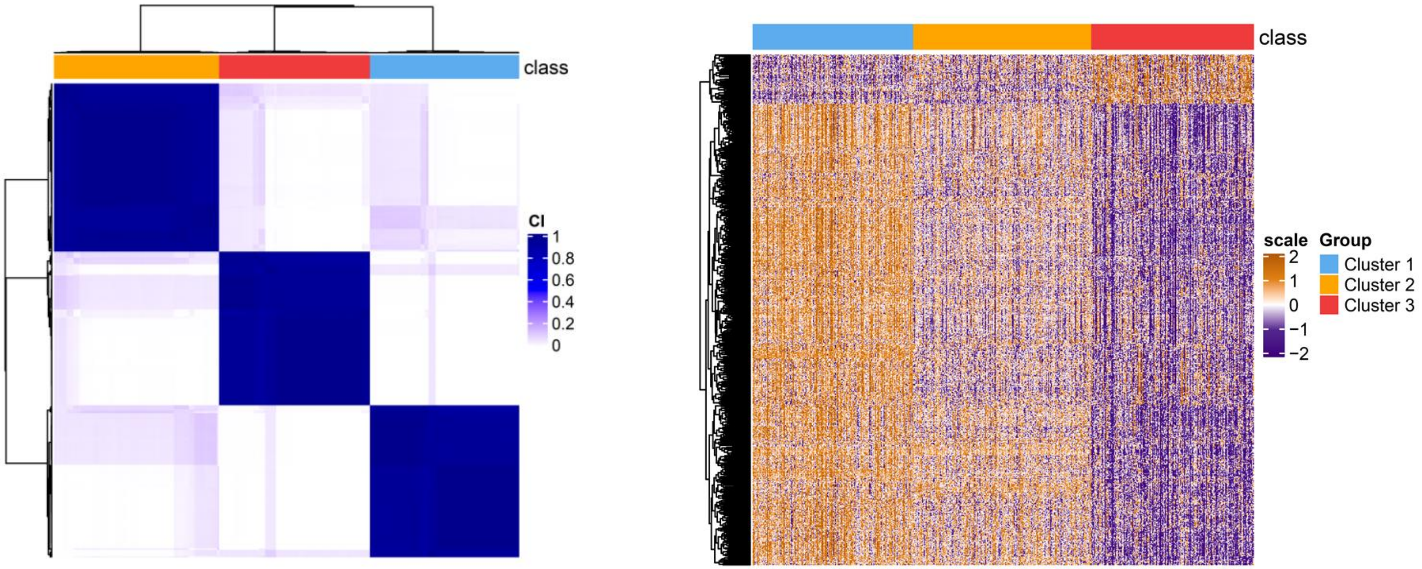

PAAD

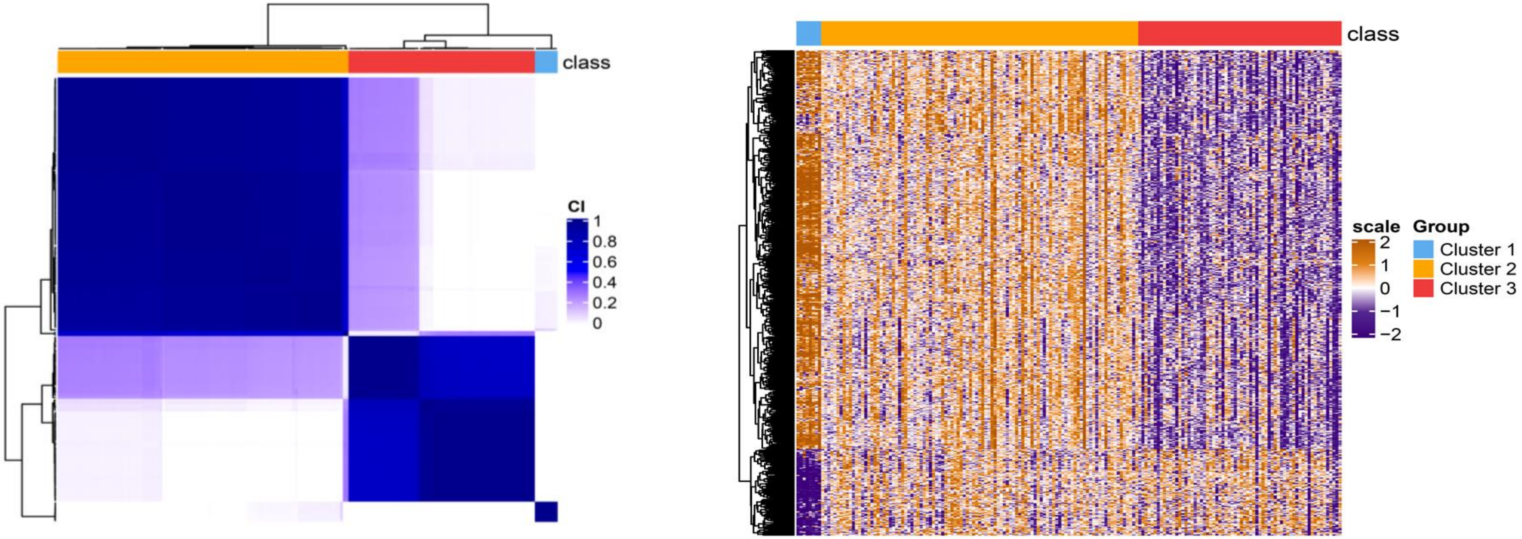

Supplement: Supplementary file 7 — Additional file 7: Figures S3. Stratification of patients into different clusters by a consensus clustering analysis. [file 12916_2021_1925_MOESM7_ESM.pdf]
