# Supplementary material for: Glycolysis-associated lncRNAs identify a subgroup of cancer patients with poor prognoses and a high-infiltration immune microenvironment
Source: BMC Med. 2021 Feb 25;19:59. doi: 10.1186/s12916-021-01925-6 (PMC7905662; doi:10.1186/s12916-021-01925-6)
Supplement: Supplementary file 9 — Additional file 9: Figures S4. Flow chart for establishing genomic classifiers. [file 12916_2021_1925_MOESM9_ESM.pdf]

Supple. Fig. 4

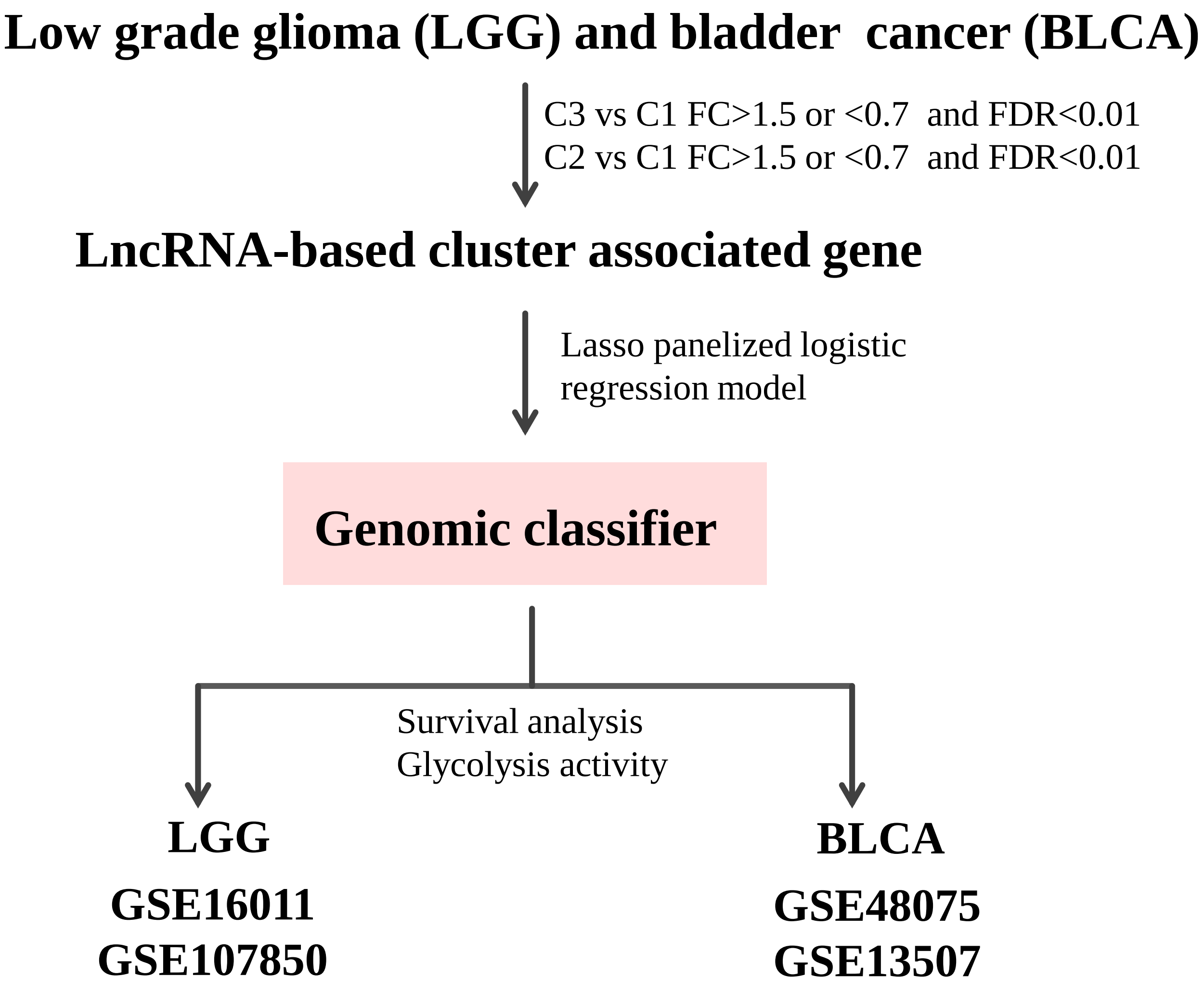

Supplemental Fig. 3. Flowchart for establishing genomic classifiers using a conducing lasso penalized lasso regression.
