# Supplementary figures and images for: Glycolysis-associated lncRNAs identify a subgroup of cancer patients with poor prognoses and a high-infiltration immune microenvironment
Source: BMC Med. 2021 Feb 25;19:59. doi: 10.1186/s12916-021-01925-6 (PMC7905662; doi:10.1186/s12916-021-01925-6)

**Supple. Fig. 5**

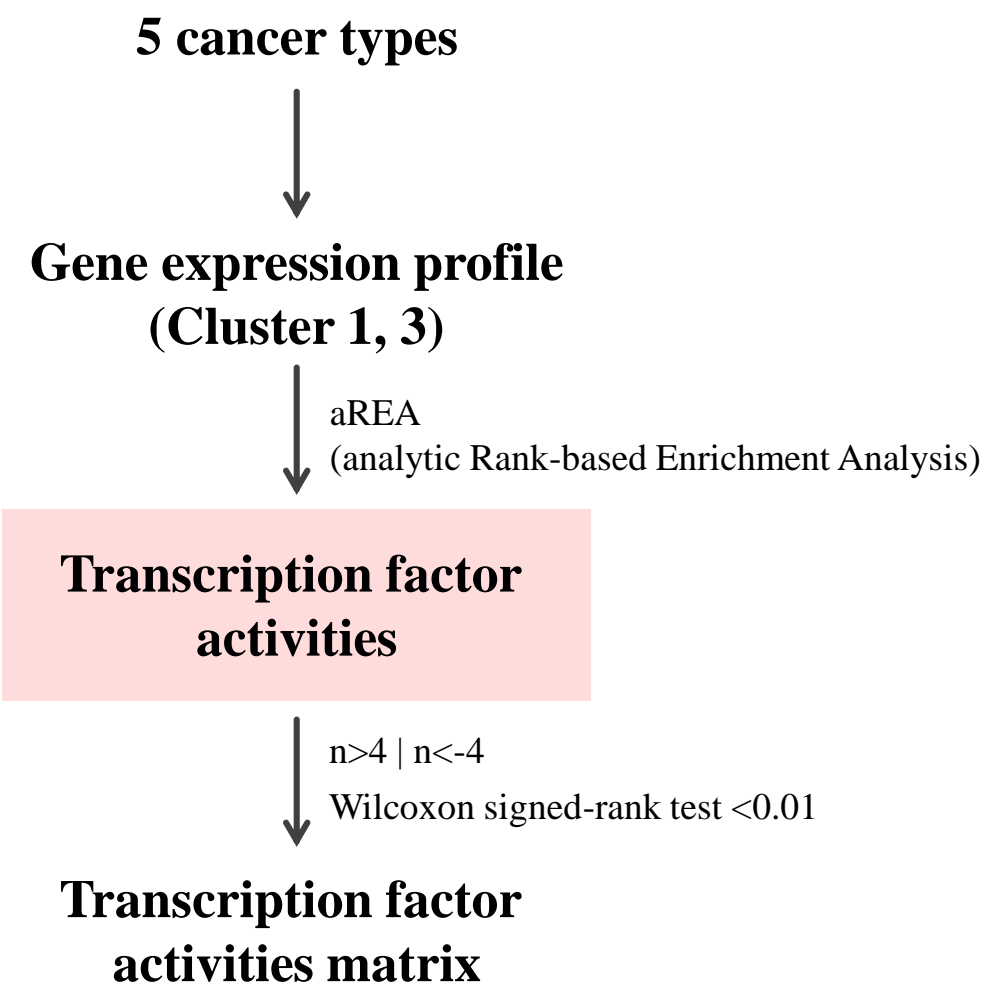

**Supplemental Fig. 5. Flowchart for transcription factor activity analyses.**

Supplement: Supplementary file 10 — Additional file 10: Figures S5. Flow chart for Transcription factor activity analyses. [file 12916_2021_1925_MOESM10_ESM.pdf]
