# Supplementary material for: Glycolysis-associated lncRNAs identify a subgroup of cancer patients with poor prognoses and a high-infiltration immune microenvironment
Source: BMC Med. 2021 Feb 25;19:59. doi: 10.1186/s12916-021-01925-6 (PMC7905662; doi:10.1186/s12916-021-01925-6)
Supplement: Supplementary file 12 — Additional file 12: Figures S7. Positive correlation of lncRNA-MYC activity pairs. [file 12916_2021_1925_MOESM12_ESM.pdf]

Supple. Fig. 7

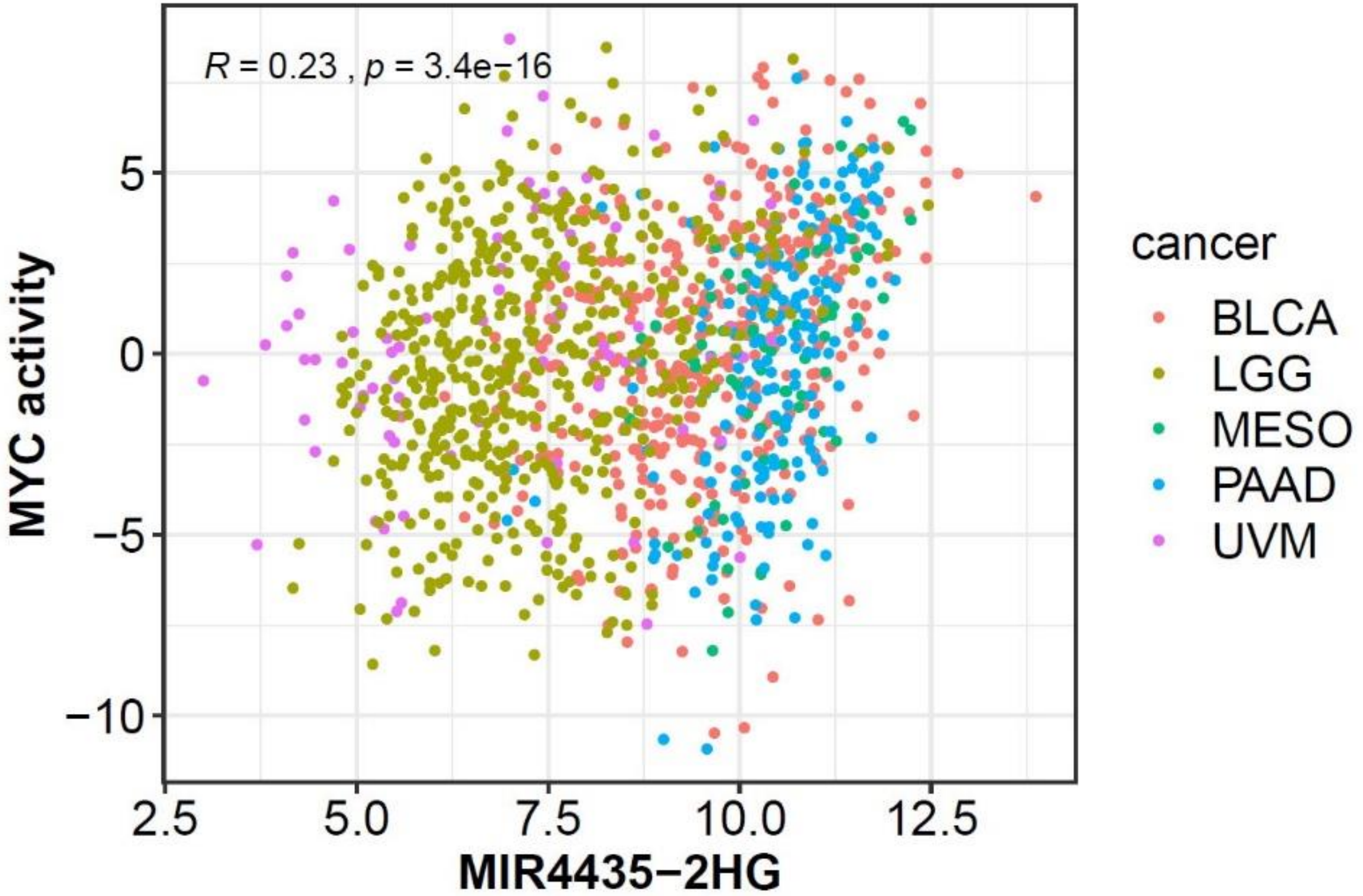

Supplemental Fig. 7. Dot plots of lncRNA-MYC activity pairs that exhibit positive correlations across four cancer types.
