# Supplementary material for: Glycolysis-associated lncRNAs identify a subgroup of cancer patients with poor prognoses and a high-infiltration immune microenvironment
Source: BMC Med. 2021 Feb 25;19:59. doi: 10.1186/s12916-021-01925-6 (PMC7905662; doi:10.1186/s12916-021-01925-6)
Supplement: Supplementary file 13 — Additional file 13: Figures S8. All the high resolution images in the result section. [file 12916_2021_1925_MOESM13_ESM.pdf]

Fig. 1B

BLCA

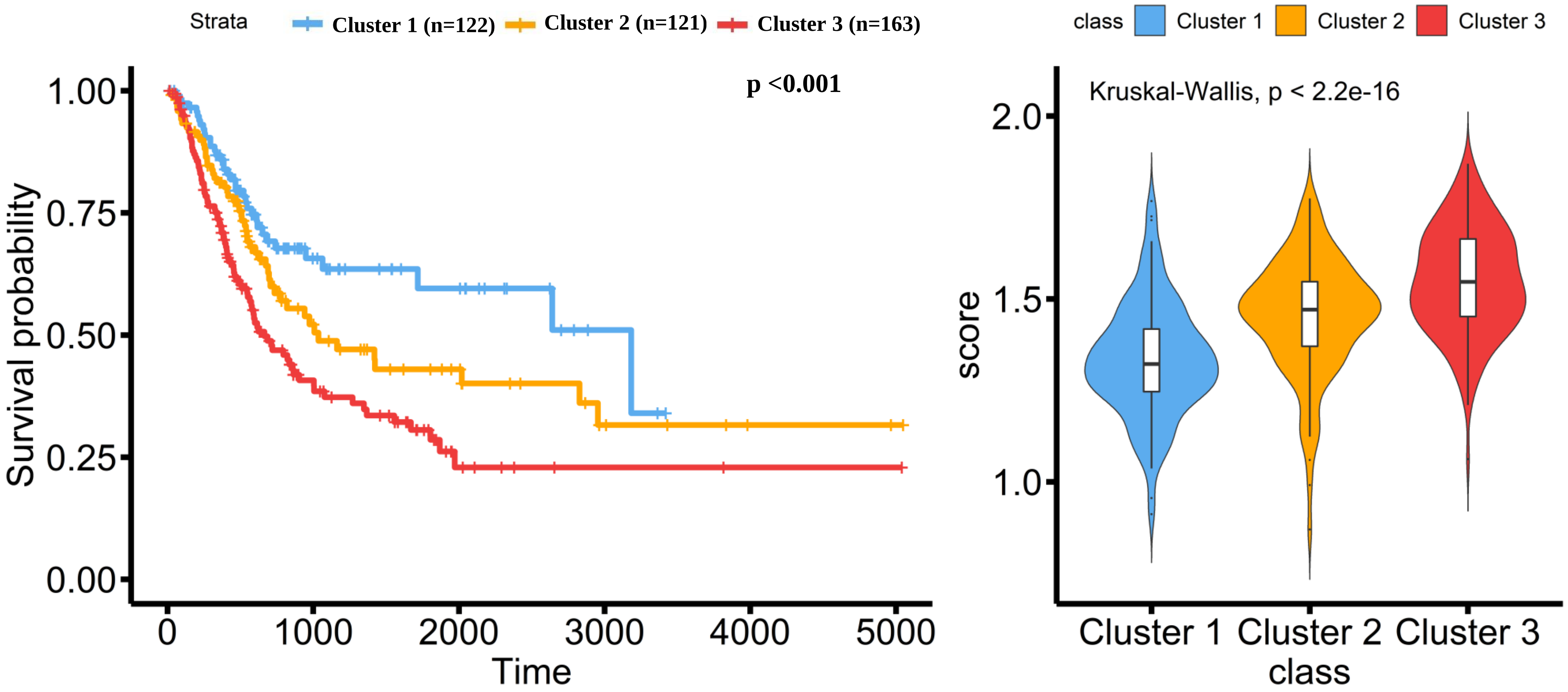

Strata
Cluster 1 (n=122)
Cluster 2 (n=121)
Cluster 3 (n=163)
p <0.001
Survival probability
1.00
0.75
0.50
0.25
0.00
0
1000
2000
3000
4000
5000
Time
class
Cluster 1
Cluster 2
Cluster 3
Kruskal-Wallis, p < 2.2e-16
score
2.0
1.5
1.0
Cluster 1
Cluster 2
Cluster 3
class

Fig. 1B

PAAD

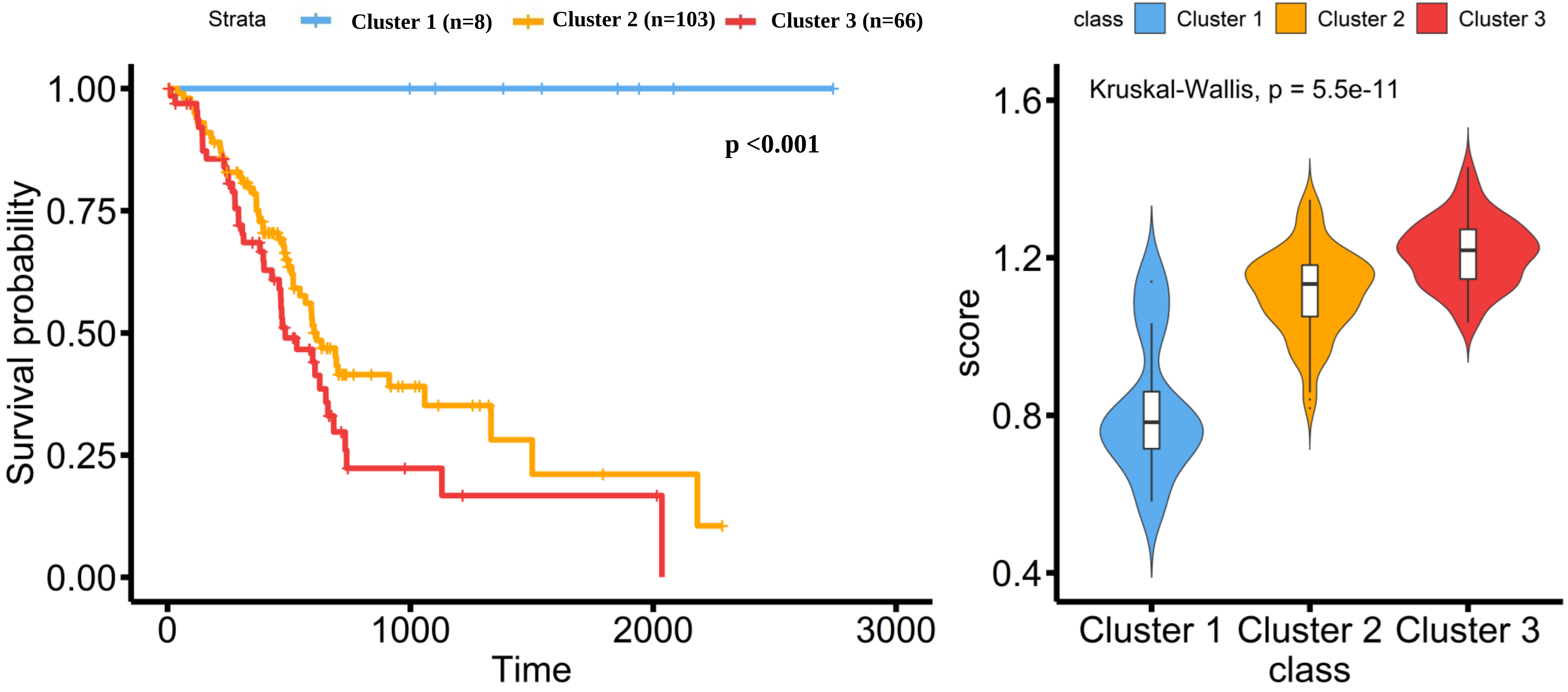

Strata
Cluster 1 (n=8)
Cluster 2 (n=103)
Cluster 3 (n=66)
Survival probability
1.00
0.75
0.50
0.25
0.00
p <0.001
0
1000
2000
3000
Time
class
Cluster 1
Cluster 2
Cluster 3
Kruskal-Wallis, p = 5.5e-11
score
1.6
1.2
0.8
0.4
Cluster 1
Cluster 2
Cluster 3
class

Fig. 1B

LGG

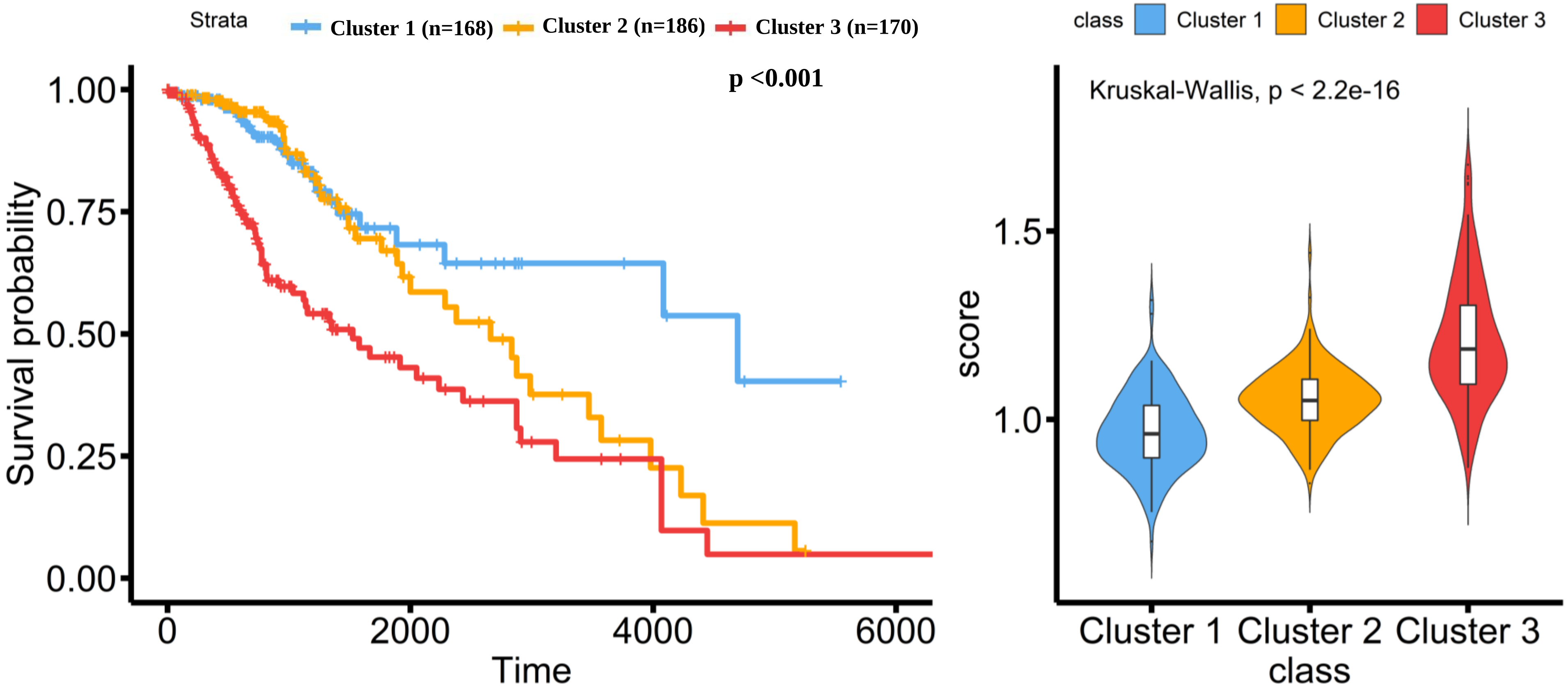

Strata
Cluster 1 (n=168)
Cluster 2 (n=186)
Cluster 3 (n=170)
p <0.001
Survival probability
1.00
0.75
0.50
0.25
0.00
0
2000
4000
6000
Time
class
Cluster 1
Cluster 2
Cluster 3
Kruskal-Wallis, p < 2.2e-16
score
1.5
1.0
Cluster 1
Cluster 2
Cluster 3
class

Fig. 1B

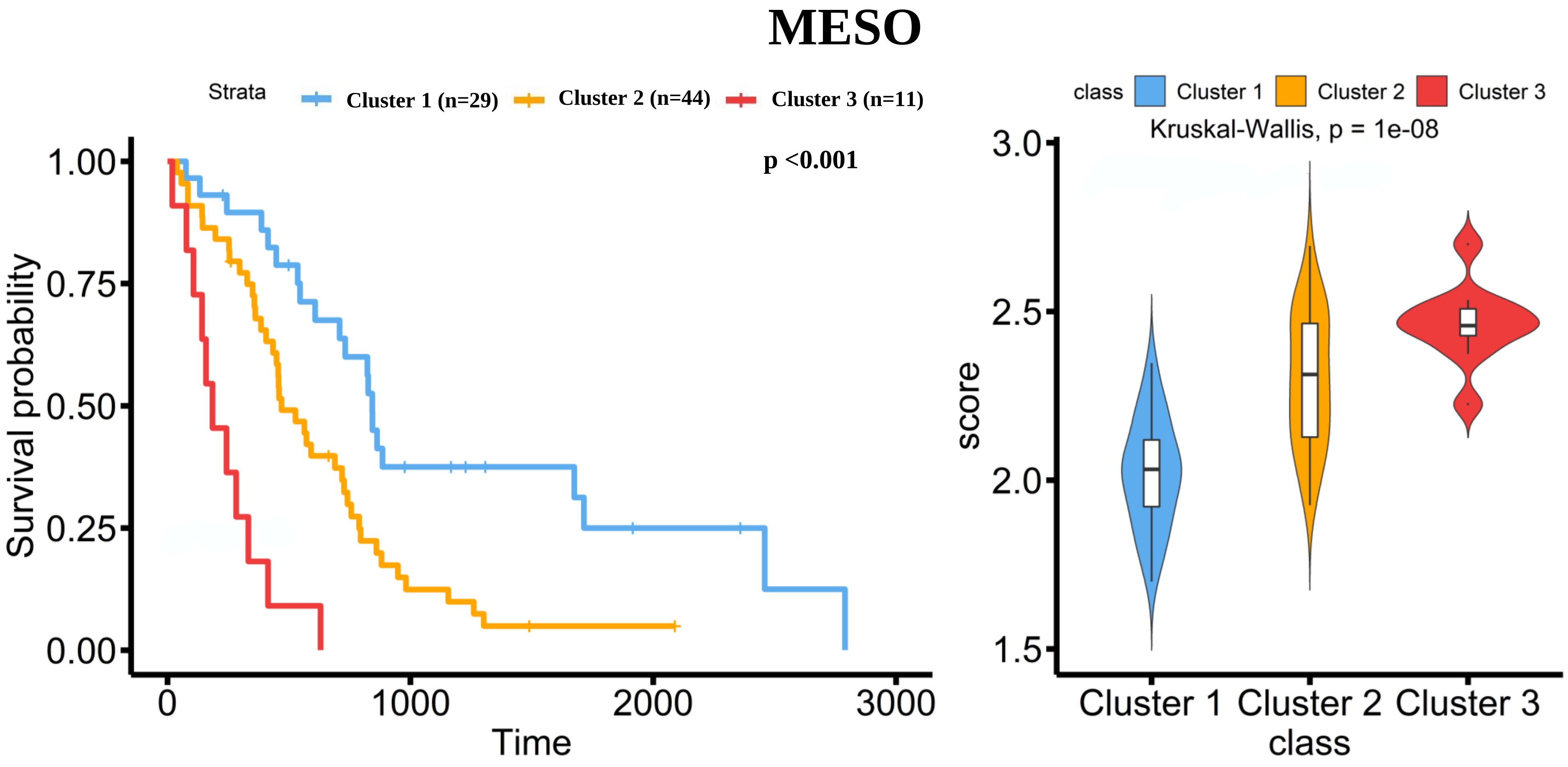
MESO
Strata
Cluster 1 (n=29)
Cluster 2 (n=44)
Cluster 3 (n=11)
p <0.001
Survival probability
1.00
0.75
0.50
0.25
0.00
0
1000
2000
3000
Time
class
Cluster 1
Cluster 2
Cluster 3
Kruskal-Wallis, p = 1e-08
score
3.0
2.5
2.0
1.5
Cluster 1
Cluster 2
Cluster 3
class

Fig. 1B

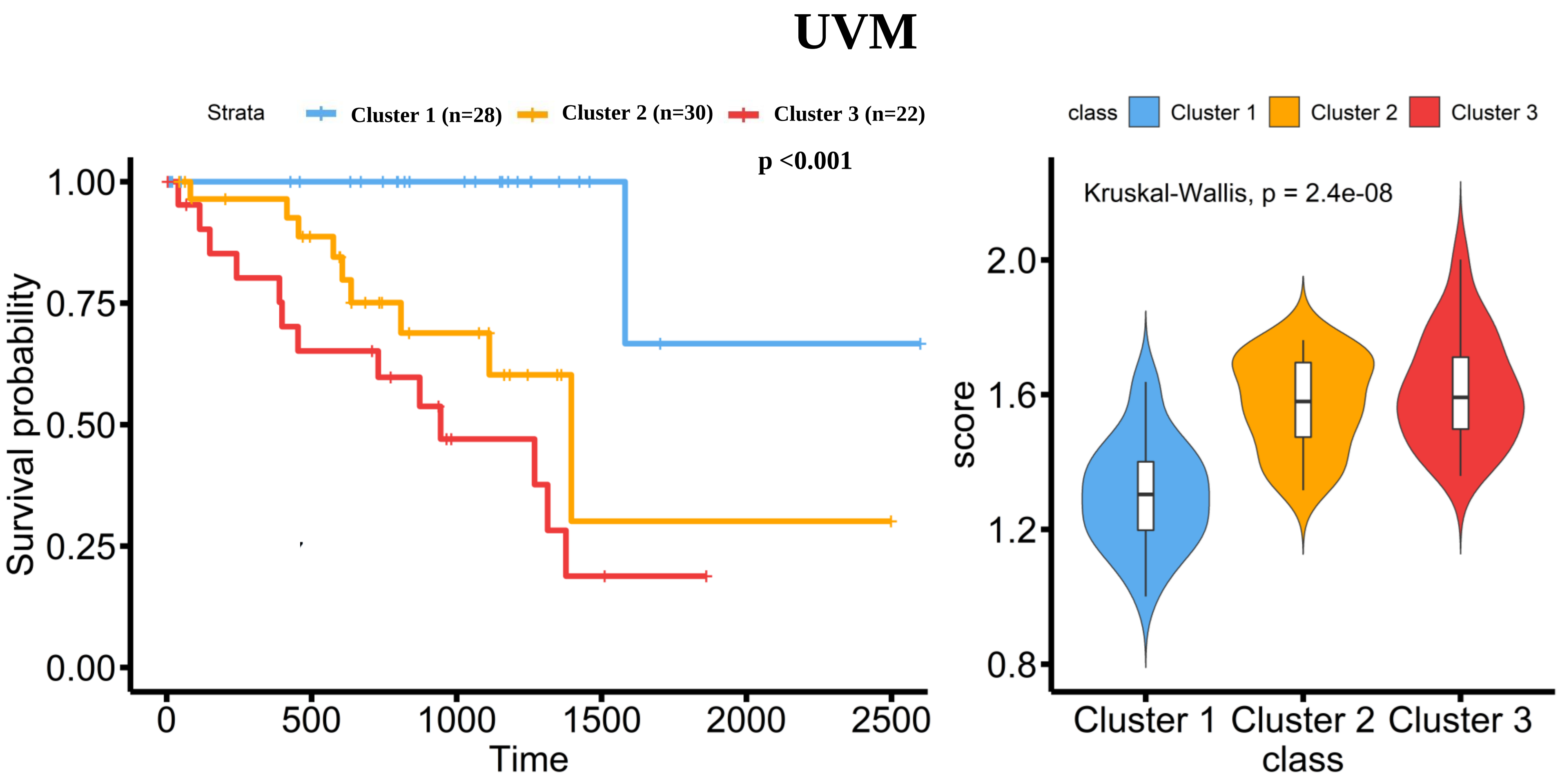
UVM
Strata
Cluster 1 (n=28)
Cluster 2 (n=30)
Cluster 3 (n=22)
p <0.001
Survival probability
1.00
0.75
0.50
0.25
0.00
0
500
1000
1500
2000
2500
Time
class
Cluster 1
Cluster 2
Cluster 3
Kruskal-Wallis, p = 2.4e-08
score
2.0
1.6
1.2
0.8
Cluster 1
Cluster 2
Cluster 3
class

Fig. 1C

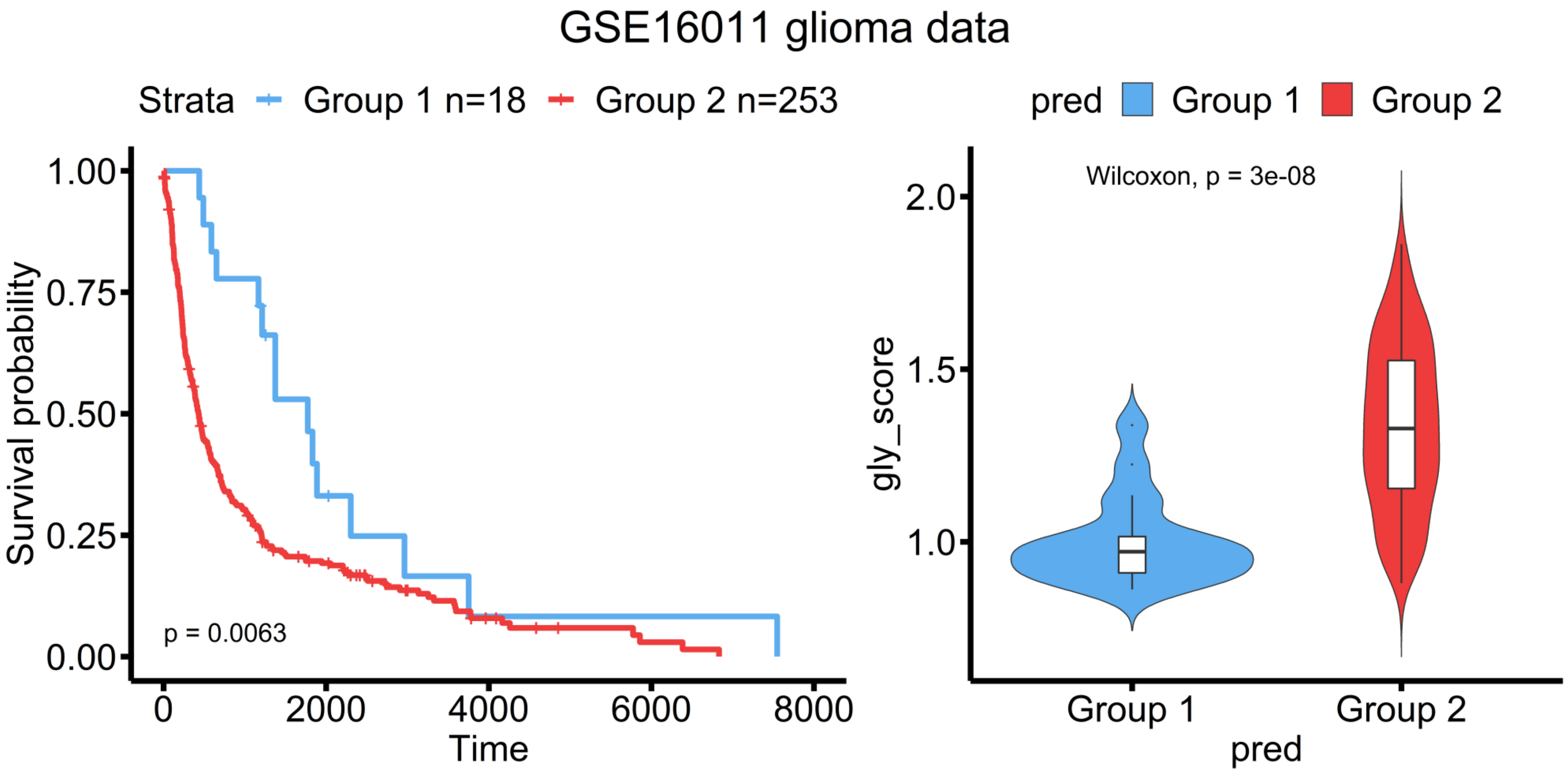
GSE16011 glioma data
Strata
Group 1 n=18
Group 2 n=253
Survival probability
1.00
0.75
0.50
0.25
0.00
p = 0.0063
0
2000
4000
6000
8000
Time
pred
Group 1
Group 2
Wilcoxon, p = 3e-08
gly_score
2.0
1.5
1.0
Group 1
Group 2
pred

Fig. 1C

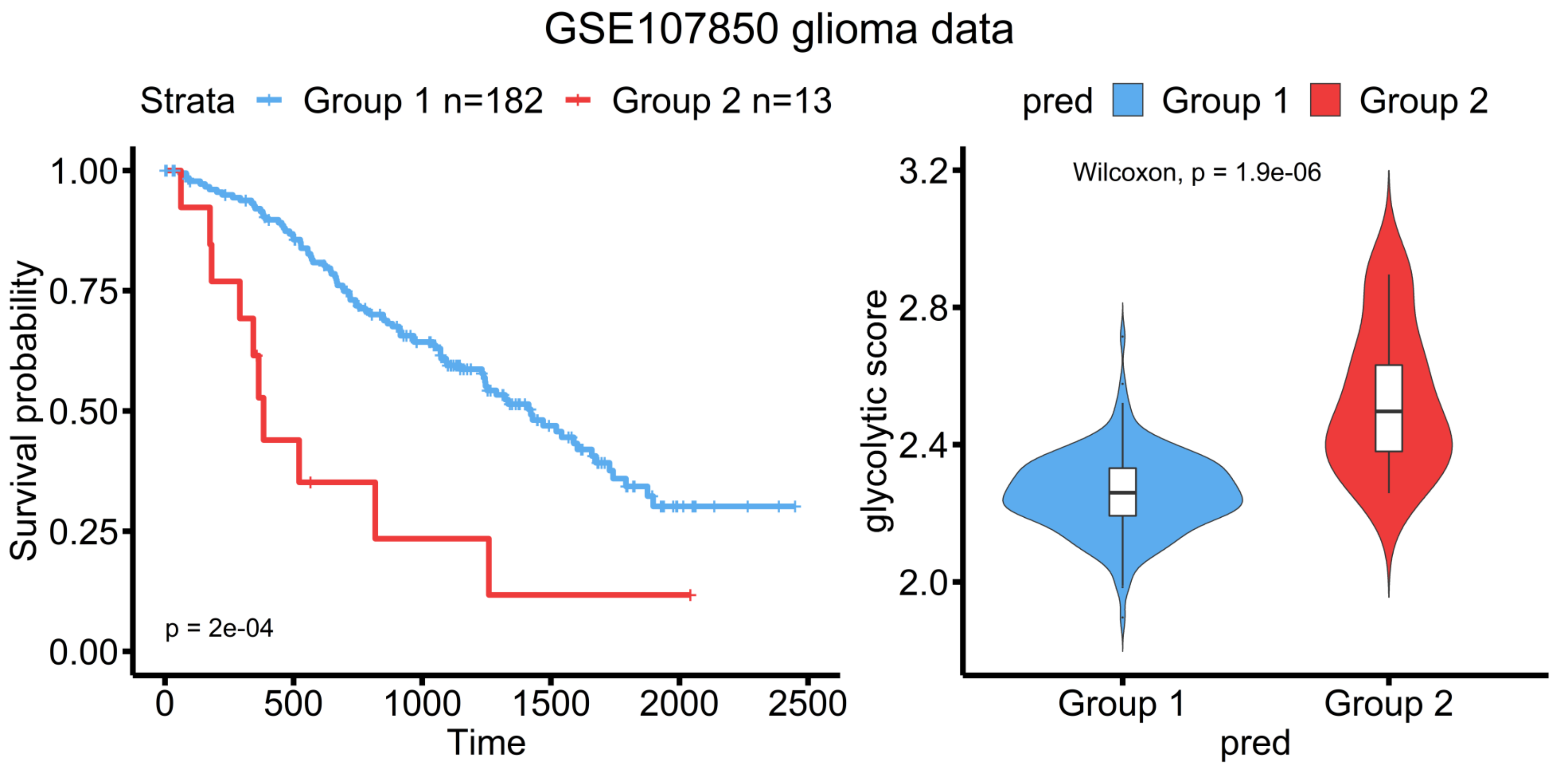
GSE107850 glioma data
Strata
Group 1 n=182
Group 2 n=13
1.00
0.75
0.50
0.25
0.00
Survival probability
p = 2e-04
0
500
1000
1500
2000
2500
Time
pred
Group 1
Group 2
Wilcoxon, p = 1.9e-06
3.2
2.8
2.4
2.0
glycolytic score
Group 1
Group 2
pred

Fig. 1D

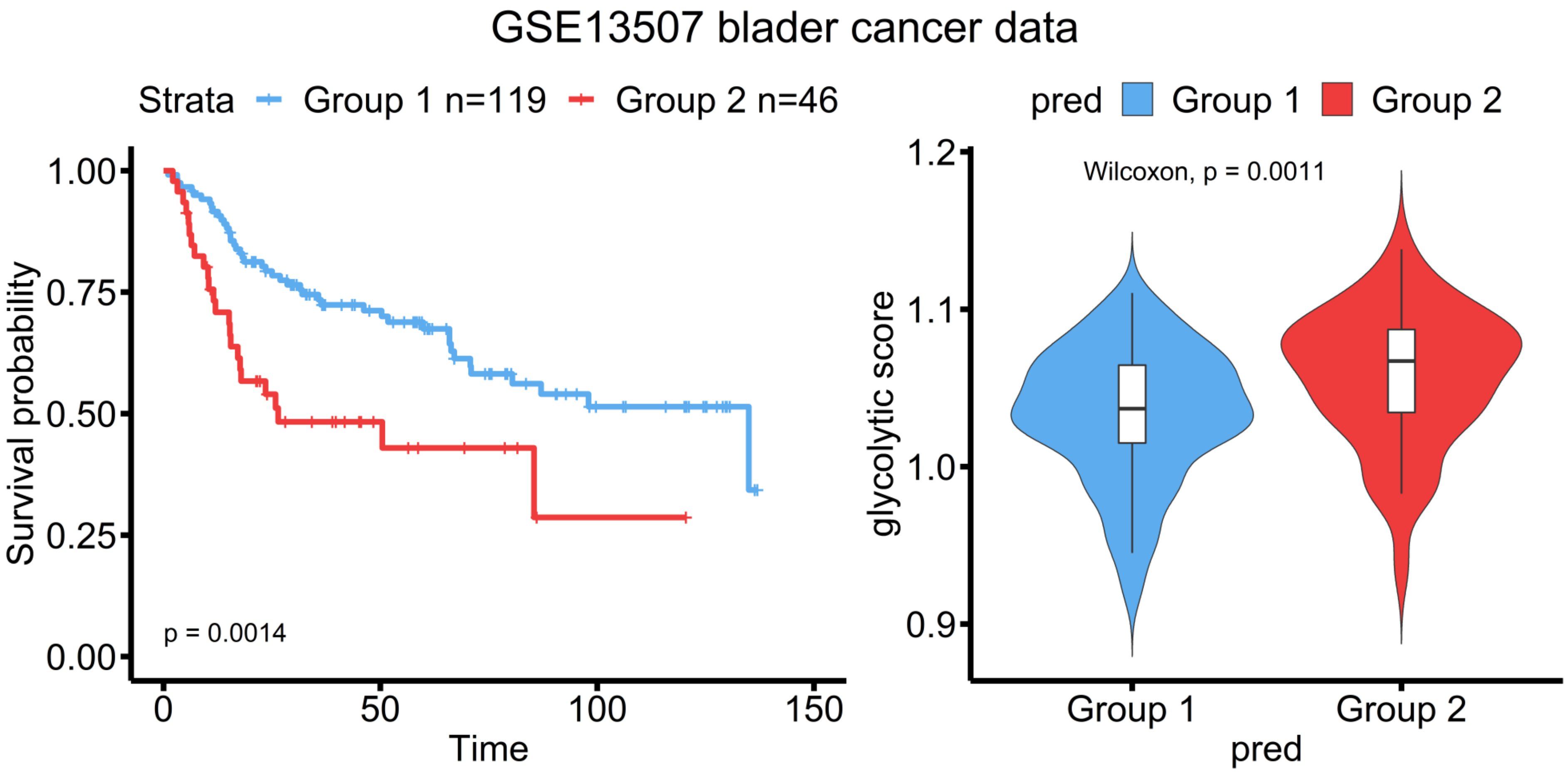

GSE13507 blader cancer data
Strata
Group 1 n=119
Group 2 n=46
1.00
0.75
0.50
0.25
0.00
Survival probability
p = 0.0014
0
50
100
150
Time
pred
Group 1
Group 2
Wilcoxon, p = 0.0011
1.2
1.1
1.0
0.9
glycolytic score
Group 1
Group 2
pred

Fig. 1D

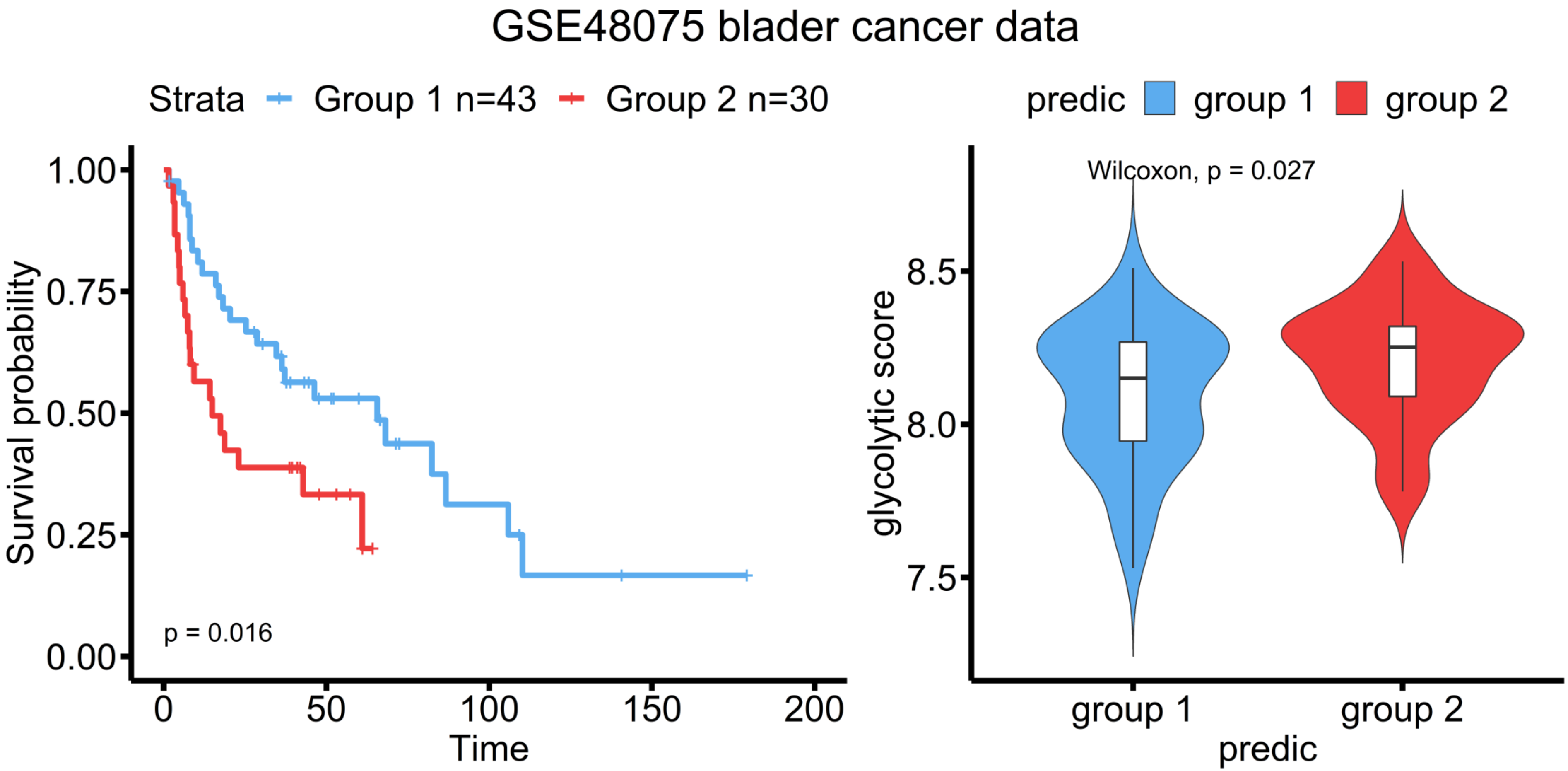
GSE48075 blader cancer data
Strata
Group 1 n=43
Group 2 n=30
Survival probability
1.00
0.75
0.50
0.25
0.00
p = 0.016
0
50
100
150
200
Time
predic
group 1
group 2
Wilcoxon, p = 0.027
glycolytic score
8.5
8.0
7.5
group 1
group 2
predic

Fig. 2A

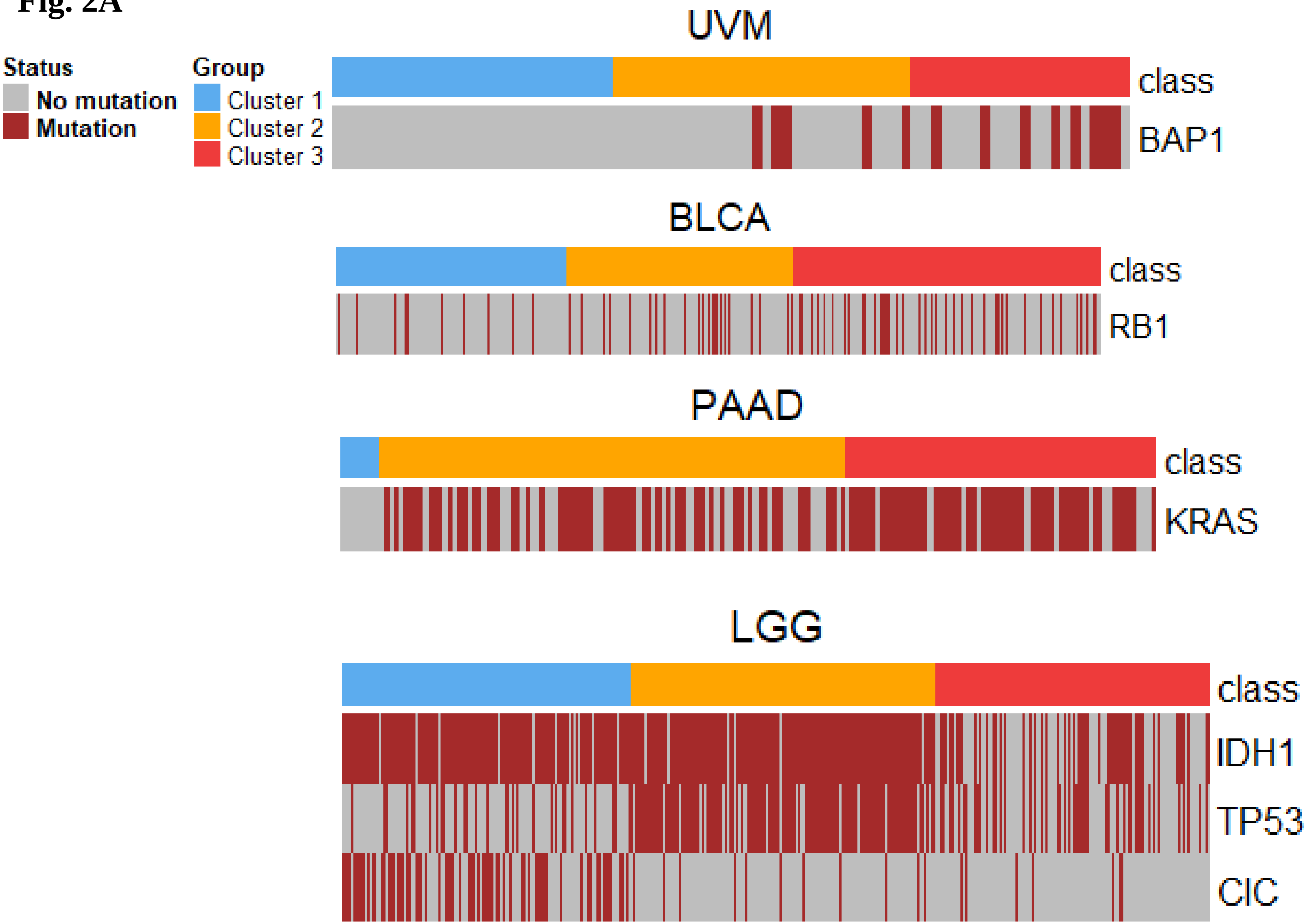

Status
No mutation
Mutation
Group
Cluster 1
Cluster 2
Cluster 3
UVM
class
BAP1
BLCA
class
RB1
PAAD
class
KRAS
LGG
class
IDH1
TP53
CIC

Fig. 2B

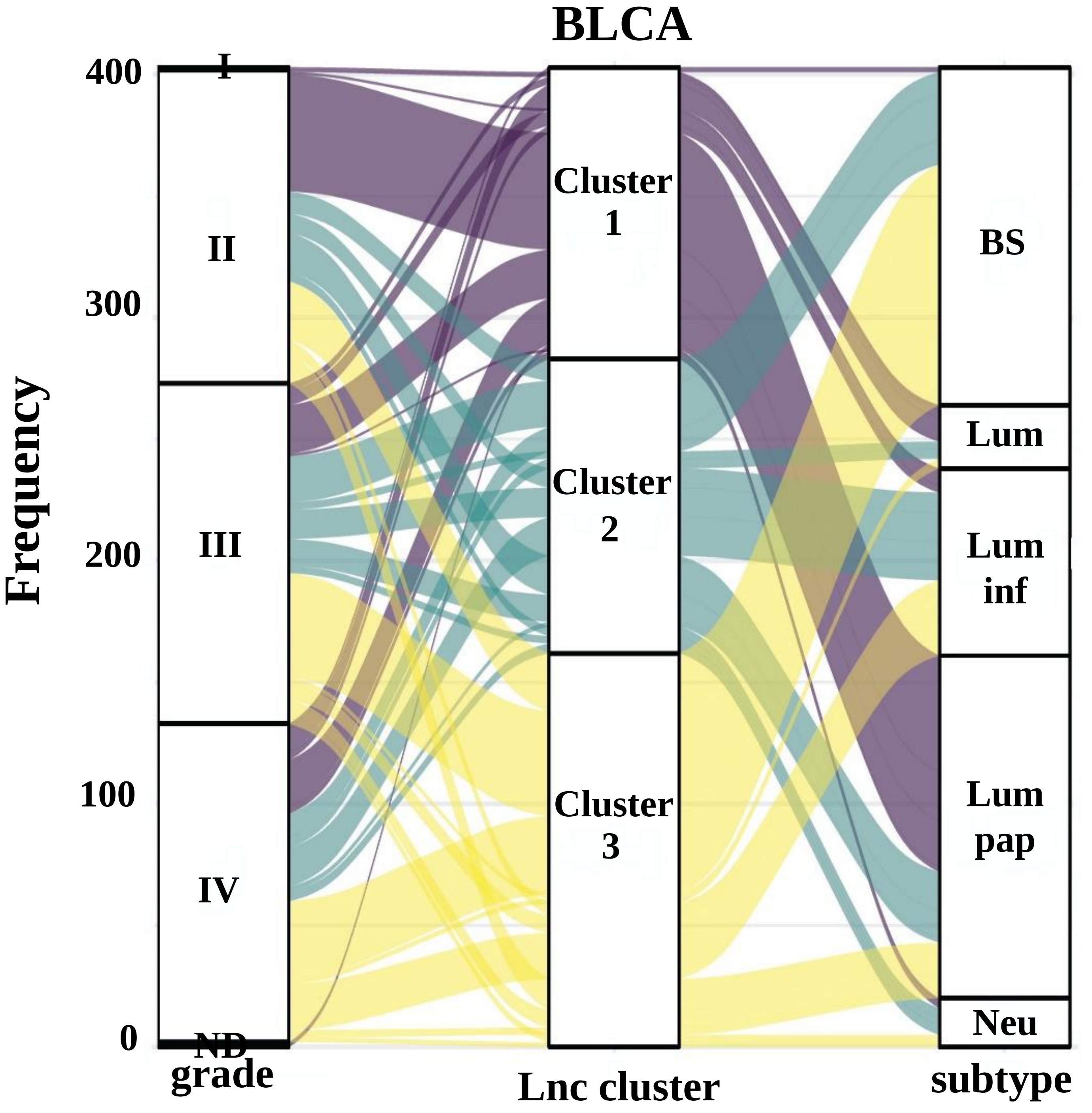
BLCA
400
300
200
100
0
Frequency
I
II
III
IV
ND
grade
Cluster 1
Cluster 2
Cluster 3
Lnc cluster
BS
Lum
Lum inf
Lum pap
Neu
subtype

Fig. 2B

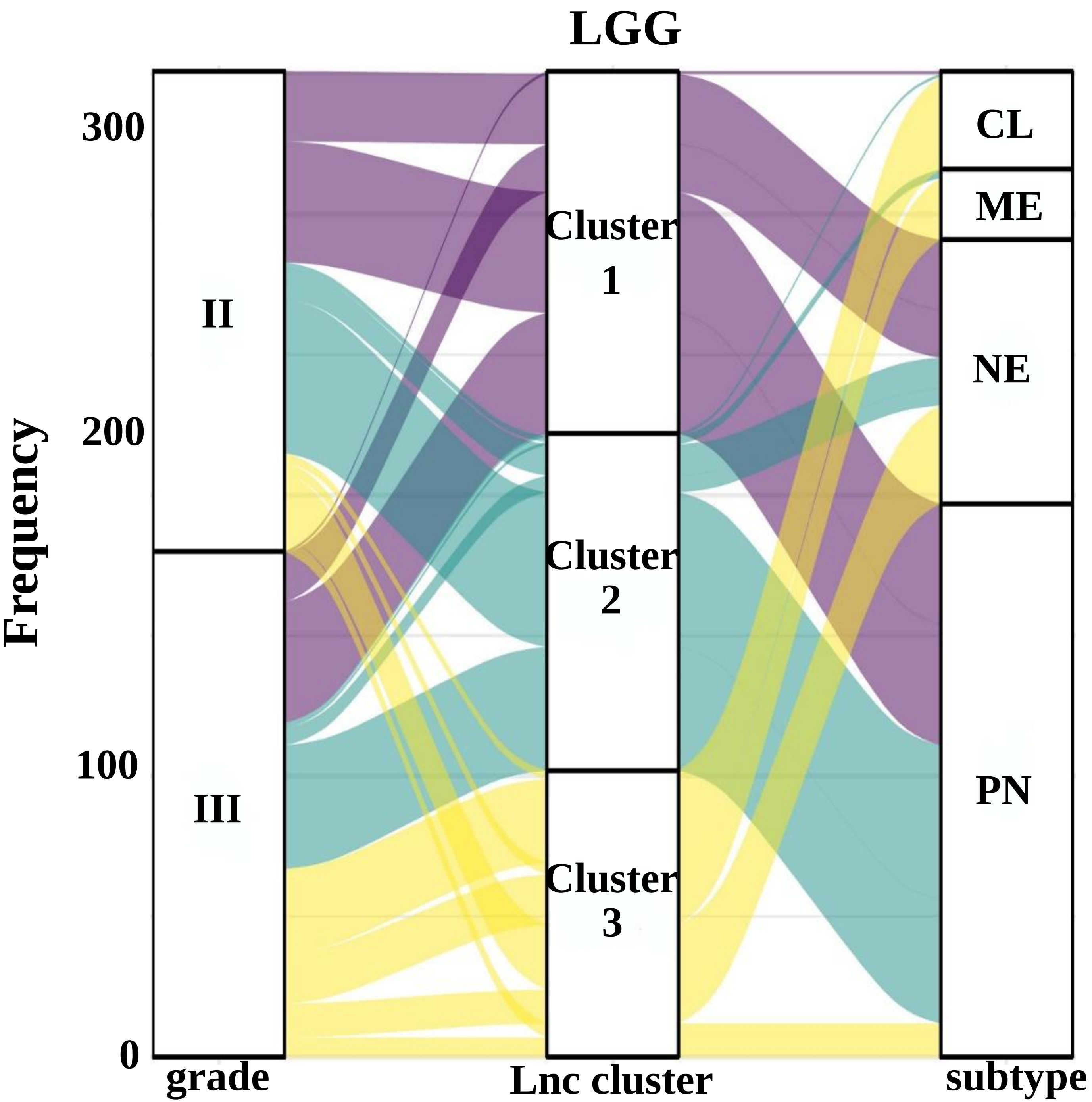

LGG
300
200
100
0
Frequency
II
III
Cluster 1
Cluster 2
Cluster 3
CL
ME
NE
PN
grade
Lnc cluster
subtype

Fig. 2B

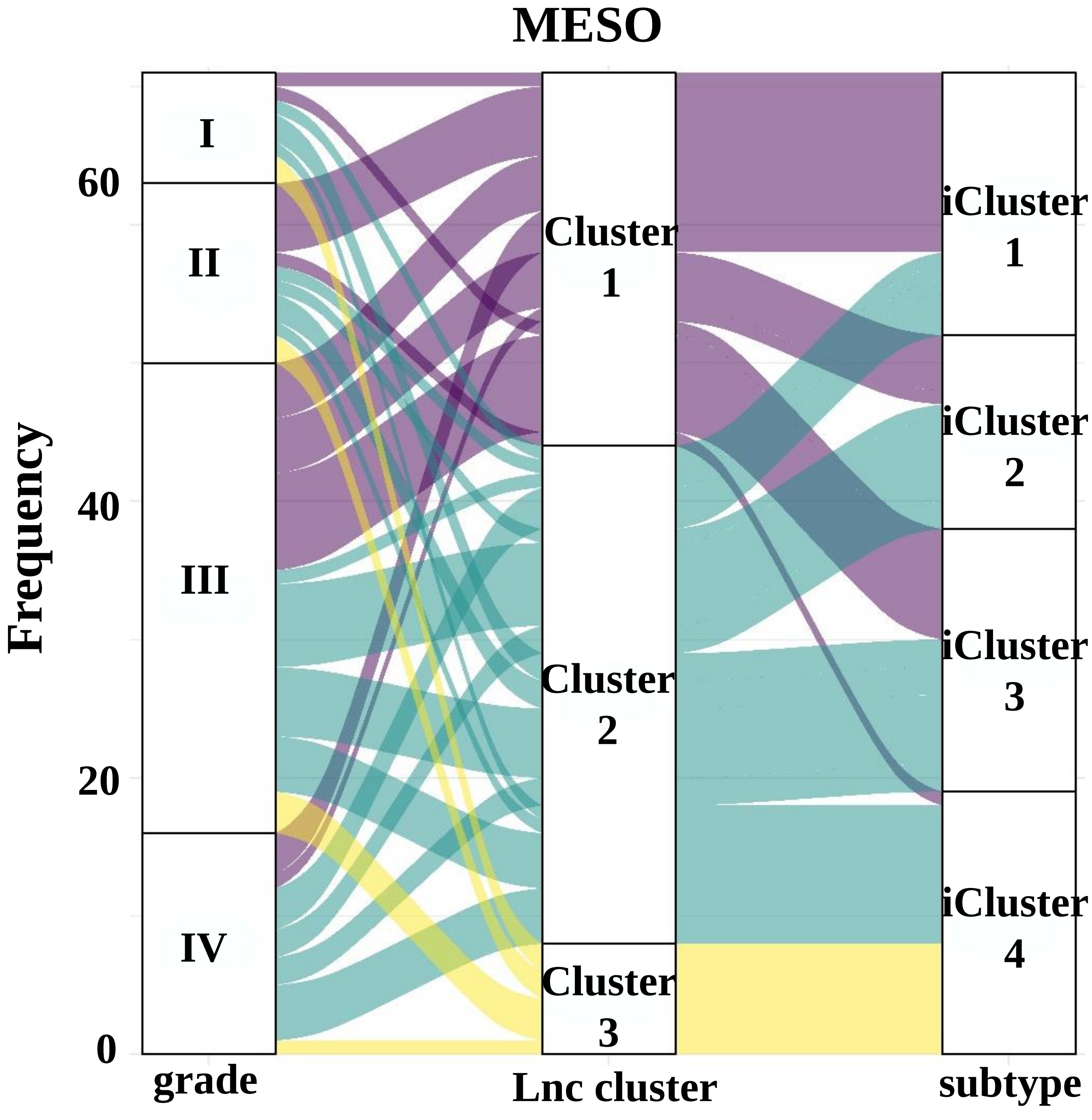
MESO
Frequency
60
40
20
0
I
II
III
IV
Cluster 1
Cluster 2
Cluster 3
iCluster 1
iCluster 2
iCluster 3
iCluster 4
grade
Lnc cluster
subtype

Fig. 2B

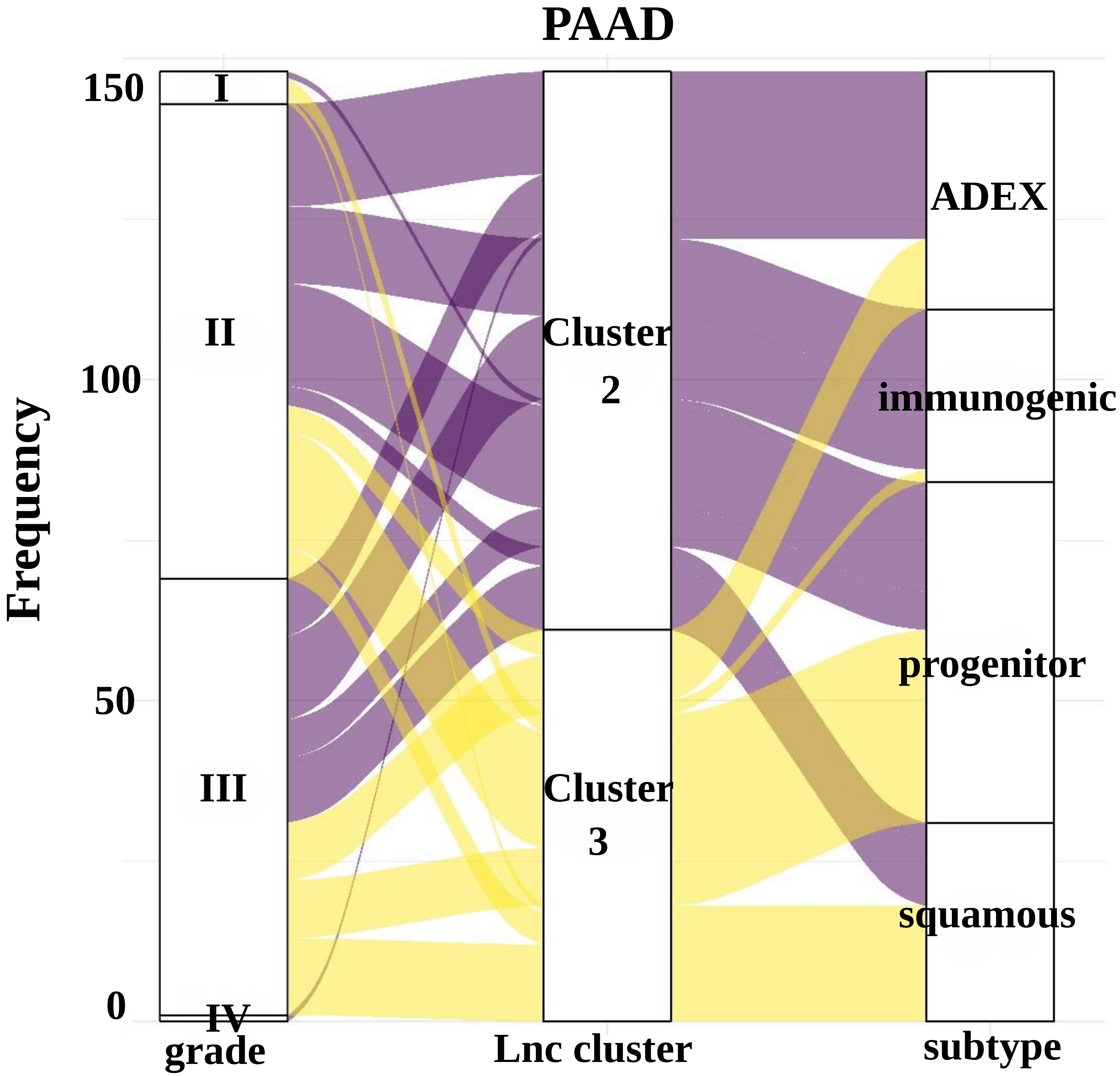
PAAD
Frequency
150
100
50
0
I
II
III
IV
Cluster 2
Cluster 3
ADEX
immunogenic
progenitor
squamous
grade
Lnc cluster
subtype

Fig. 2B

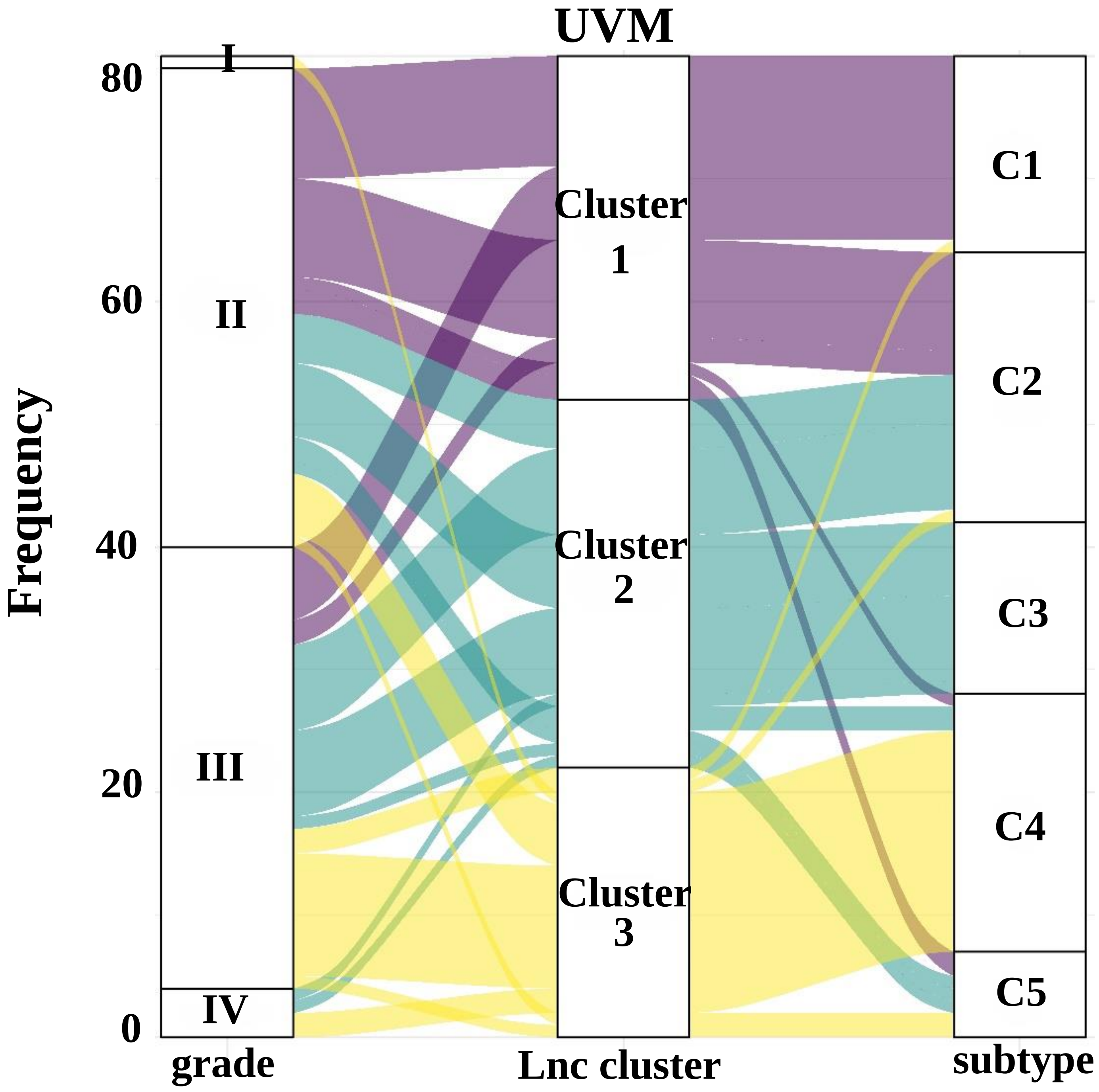
UVM
Frequency
80
60
40
20
0
I
II
III
IV
Cluster 1
Cluster 2
Cluster 3
C1
C2
C3
C4
C5
grade
Lnc cluster
subtype

Fig. 3A

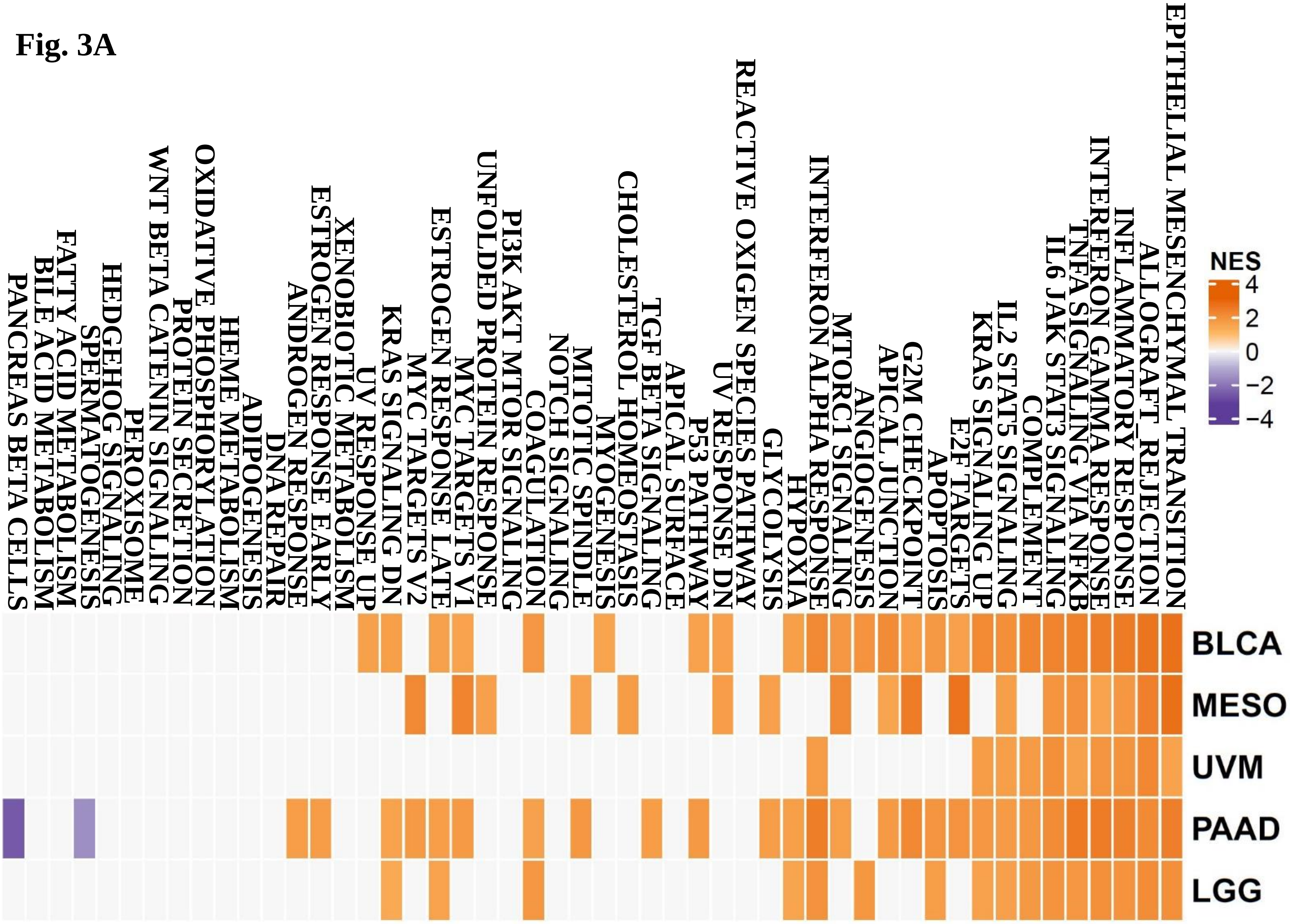
PANCREAS BETA CELLS
BILE ACID METABOLISM
FATTY ACID METABOLISM
SPERMATOGENESIS
HEDGEHOG SIGNALING
PEROXISOME
WNT BETA CATENIN SIGNALING
PROTEIN SECRETION
OXIDATIVE PHOSPHORYLATION
HEME METABOLISM
ADIPOGENESIS
DNA REPAIR
ANDROGEN RESPONSE
ESTROGEN RESPONSE EARLY
XENOBIOTIC METABOLISM
UV RESPONSE UP
KRAS SIGNALING DN
MYC TARGETS V2
ESTROGEN RESPONSE LATE
MYC TARGETS V1
UNFOLDED PROTEIN RESPONSE
PI3K AKT MTOR SIGNALING
COAGULATION
NOTCH SIGNALING
MITOTIC SPINDLE
MYOGENESIS
CHOLESTEROL HOMEOSTASIS
TGF BETA SIGNALING
APICAL SURFACE
P53 PATHWAY
UV RESPONSE DN
REACTIVE OXIGEN SPECIES PATHWAY
GLYCOLYSIS
HYPOXIA
INTERFERON ALPHA RESPONSE
MTORC1 SIGNALING
ANGIOGENESIS
APICAL JUNCTION
G2M CHECKPOINT
APOPTOSIS
E2F TARGETS
KRAS SIGNALING UP
IL2 STAT5 SIGNALING
COMPLEMENT
IL6 JAK STAT3 SIGNALING
TNFA SIGNALING VIA NFKB
INTERFERON GAMMA RESPONSE
INFLAMMATORY RESPONSE
ALLOGRAFT_REJECTION
EPITHELIAL MESENCHYMAL TRANSITION
NES
4
2
0
−2
−4
BLCA
MESO
UVM
PAAD
LGG

Fig. 3B

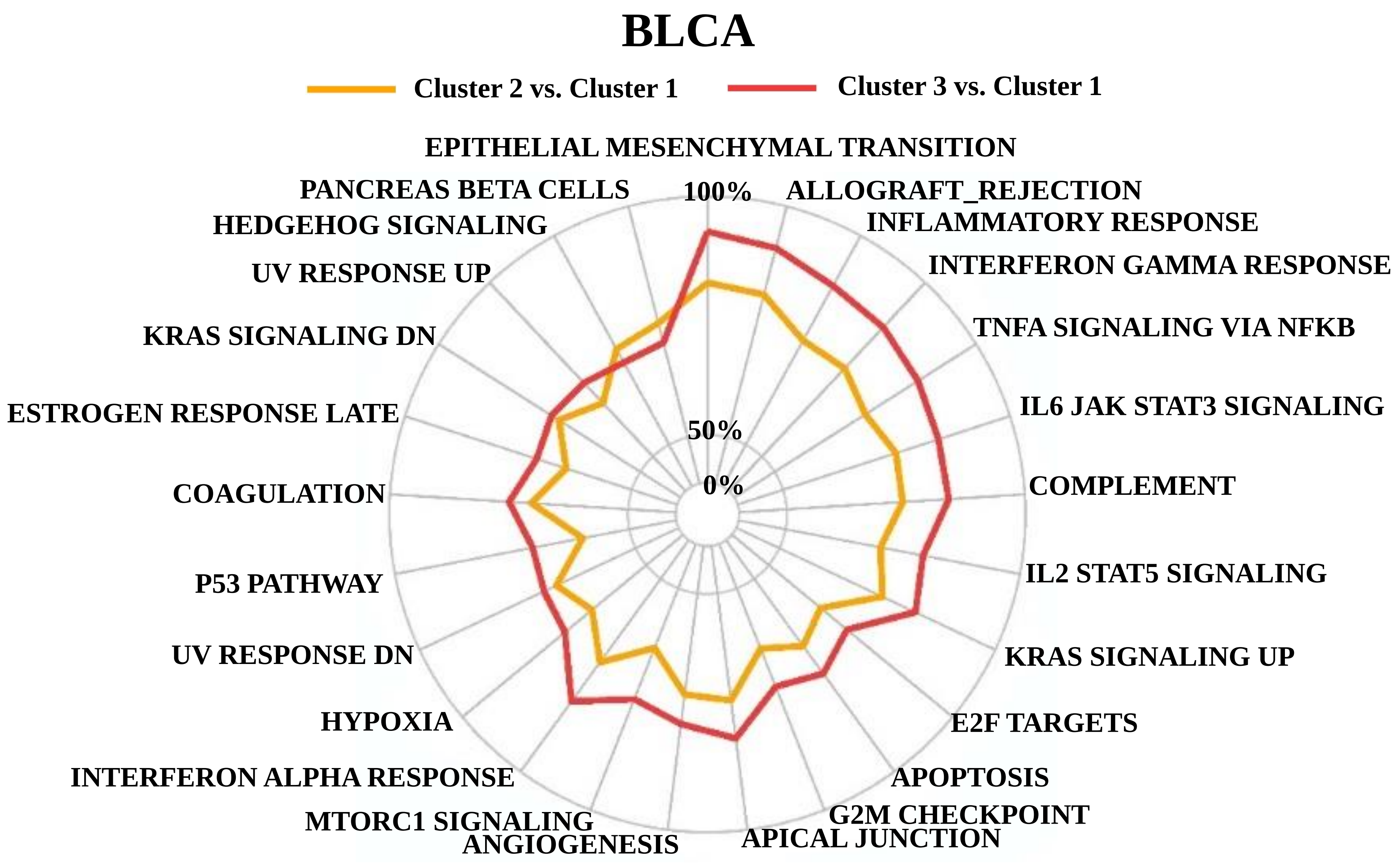

BLCA
Cluster 2 vs. Cluster 1
Cluster 3 vs. Cluster 1
EPITHELIAL MESENCHYMAL TRANSITION
100%
ALLOGRAFT_REJECTION
INFLAMMATORY RESPONSE
INTERFERON GAMMA RESPONSE
TNFA SIGNALING VIA NFKB
IL6 JAK STAT3 SIGNALING
COMPLEMENT
IL2 STAT5 SIGNALING
KRAS SIGNALING UP
E2F TARGETS
APOPTOSIS
G2M CHECKPOINT
APICAL JUNCTION
ANGIOGENESIS
MTORC1 SIGNALING
INTERFERON ALPHA RESPONSE
HYPOXIA
UV RESPONSE DN
P53 PATHWAY
COAGULATION
ESTROGEN RESPONSE LATE
KRAS SIGNALING DN
UV RESPONSE UP
HEDGEHOG SIGNALING
PANCREAS BETA CELLS
50%
0%

Fig. 3B

PAAD

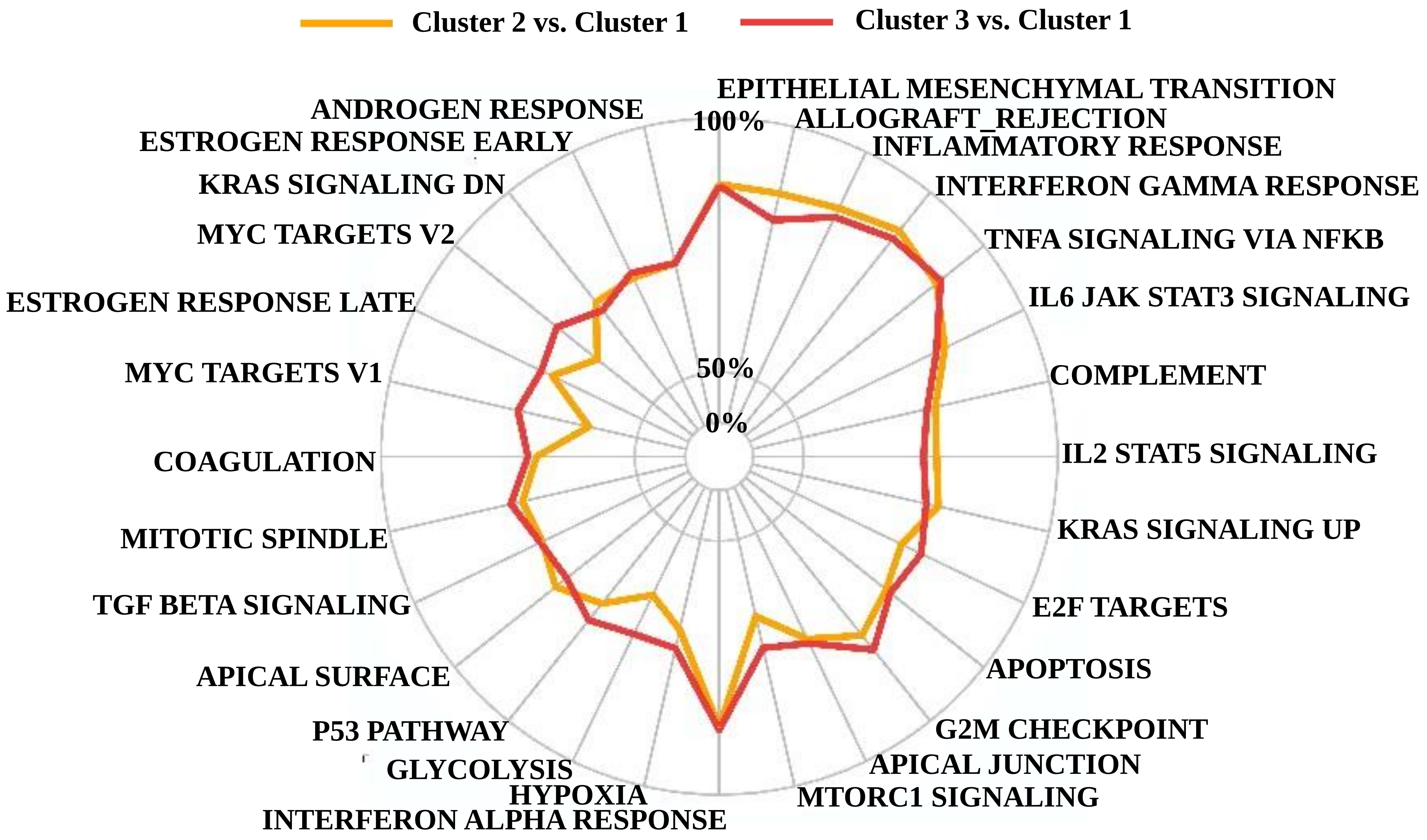
Cluster 2 vs. Cluster 1
Cluster 3 vs. Cluster 1
EPITHELIAL MESENCHYMAL TRANSITION
ALLOGRAFT_REJECTION
INFLAMMATORY RESPONSE
INTERFERON GAMMA RESPONSE
TNFA SIGNALING VIA NFKB
IL6 JAK STAT3 SIGNALING
COMPLEMENT
IL2 STAT5 SIGNALING
KRAS SIGNALING UP
E2F TARGETS
APOPTOSIS
G2M CHECKPOINT
APICAL JUNCTION
MTORC1 SIGNALING
INTERFERON ALPHA RESPONSE
HYPOXIA
GLYCOLYSIS
P53 PATHWAY
APICAL SURFACE
TGF BETA SIGNALING
MITOTIC SPINDLE
COAGULATION
MYC TARGETS V1
ESTROGEN RESPONSE LATE
MYC TARGETS V2
KRAS SIGNALING DN
ESTROGEN RESPONSE EARLY
ANDROGEN RESPONSE
100%
50%
0%

Fig. 3B

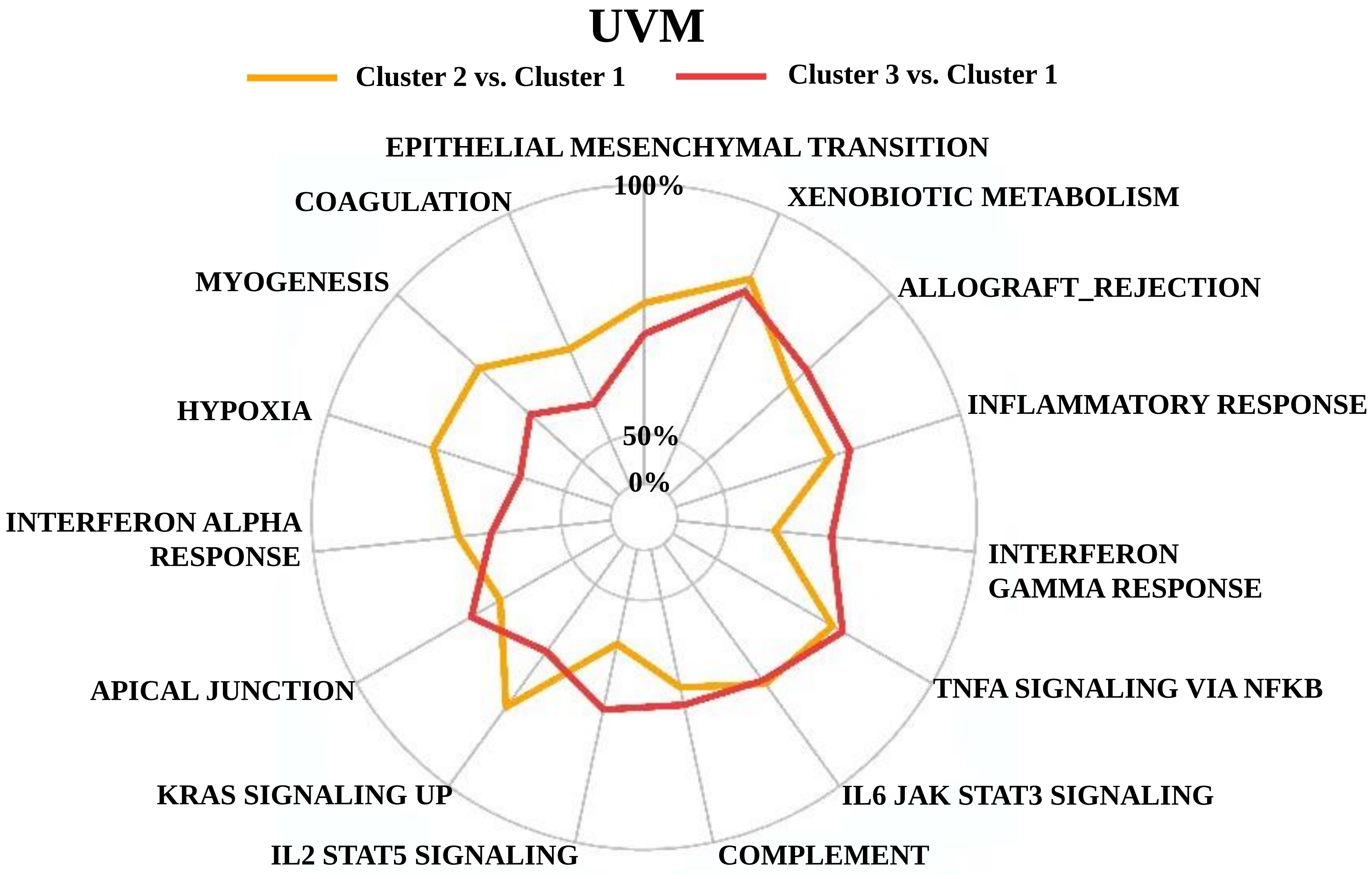
UVM
Cluster 2 vs. Cluster 1
Cluster 3 vs. Cluster 1
EPITHELIAL MESENCHYMAL TRANSITION
100%
XENOBIOTIC METABOLISM
ALLOGRAFT_REJECTION
INFLAMMATORY RESPONSE
INTERFERON GAMMA RESPONSE
TNFA SIGNALING VIA NFKB
IL6 JAK STAT3 SIGNALING
COMPLEMENT
IL2 STAT5 SIGNALING
KRAS SIGNALING UP
APICAL JUNCTION
INTERFERON ALPHA RESPONSE
HYPOXIA
MYOGENESIS
COAGULATION
50%
0%

Fig. 3B

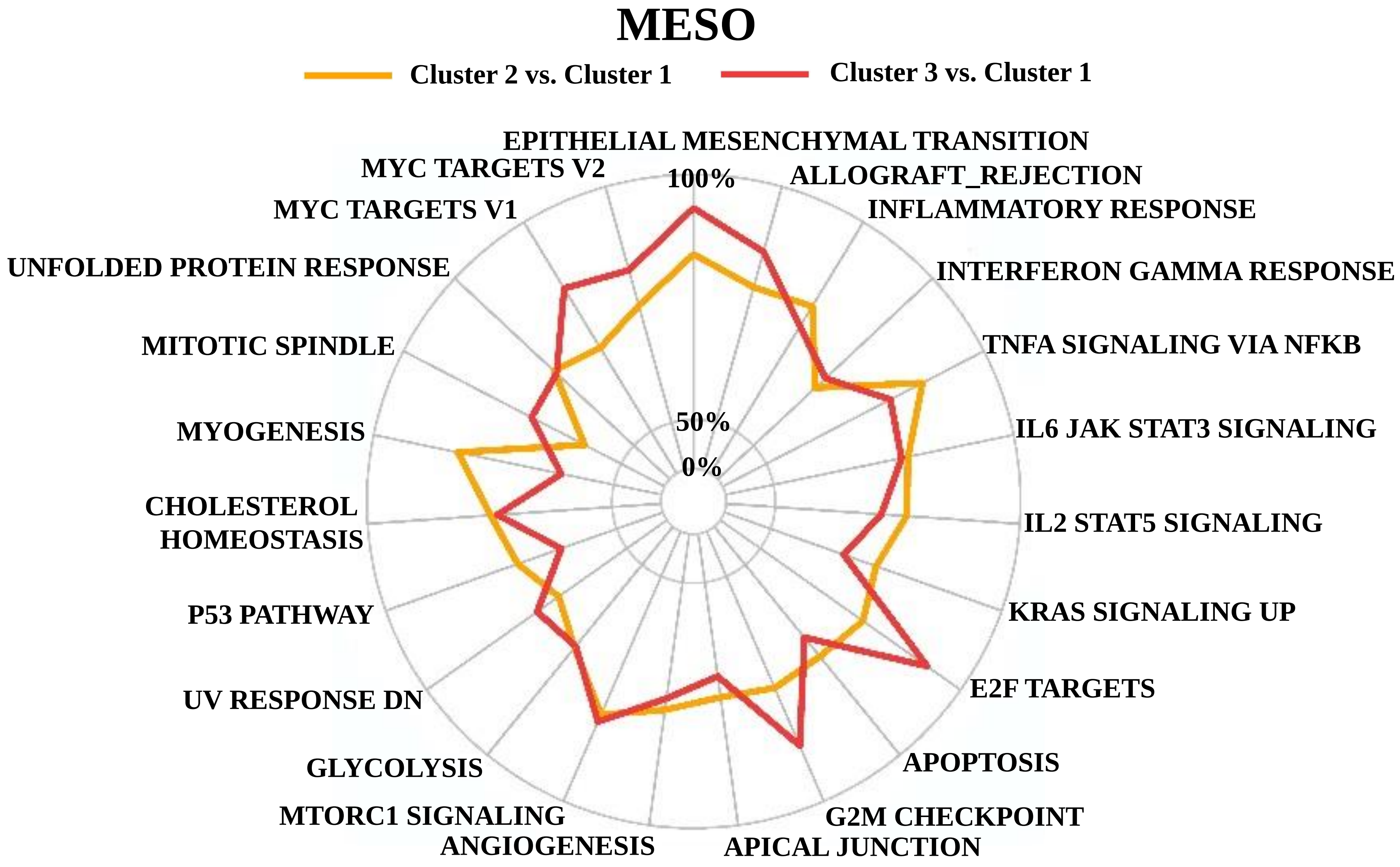
MESO
Cluster 2 vs. Cluster 1
Cluster 3 vs. Cluster 1
EPITHELIAL MESENCHYMAL TRANSITION
ALLOGRAFT_REJECTION
INFLAMMATORY RESPONSE
INTERFERON GAMMA RESPONSE
TNFA SIGNALING VIA NFKB
IL6 JAK STAT3 SIGNALING
IL2 STAT5 SIGNALING
KRAS SIGNALING UP
E2F TARGETS
APOPTOSIS
G2M CHECKPOINT
APICAL JUNCTION
ANGIOGENESIS
MTORC1 SIGNALING
GLYCOLYSIS
UV RESPONSE DN
P53 PATHWAY
CHOLESTEROL HOMEOSTASIS
MYOGENESIS
MITOTIC SPINDLE
UNFOLDED PROTEIN RESPONSE
MYC TARGETS V1
MYC TARGETS V2
100%
50%
0%

Fig. 3B

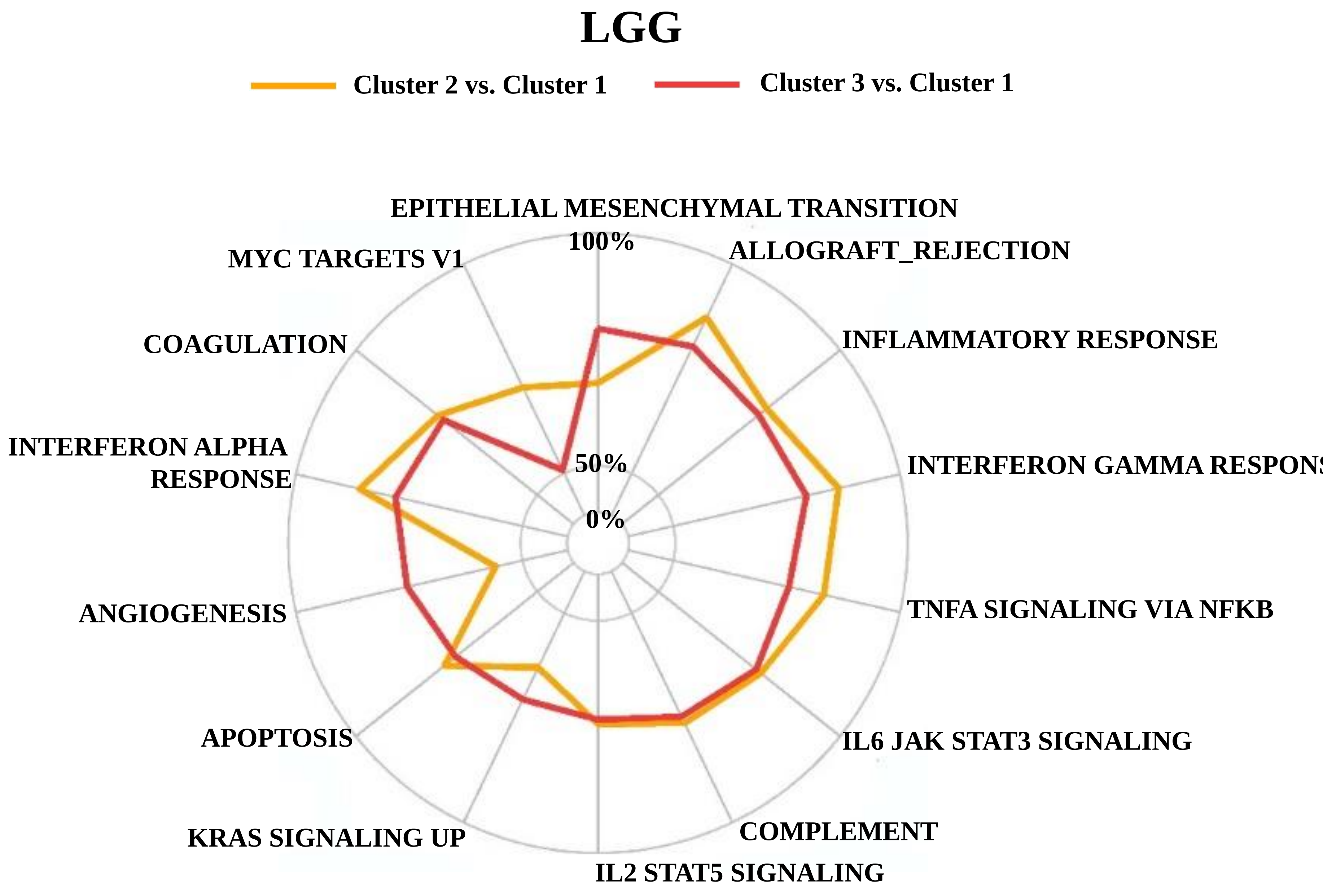

LGG
Cluster 2 vs. Cluster 1
Cluster 3 vs. Cluster 1
EPITHELIAL MESENCHYMAL TRANSITION
100%
ALLOGRAFT_REJECTION
MYC TARGETS V1
COAGULATION
INFLAMMATORY RESPONSE
INTERFERON ALPHA RESPONSE
50%
INTERFERON GAMMA RESPONS
0%
ANGIOGENESIS
TNFA SIGNALING VIA NFKB
APOPTOSIS
IL6 JAK STAT3 SIGNALING
KRAS SIGNALING UP
COMPLEMENT
IL2 STAT5 SIGNALING

Fig. 4A

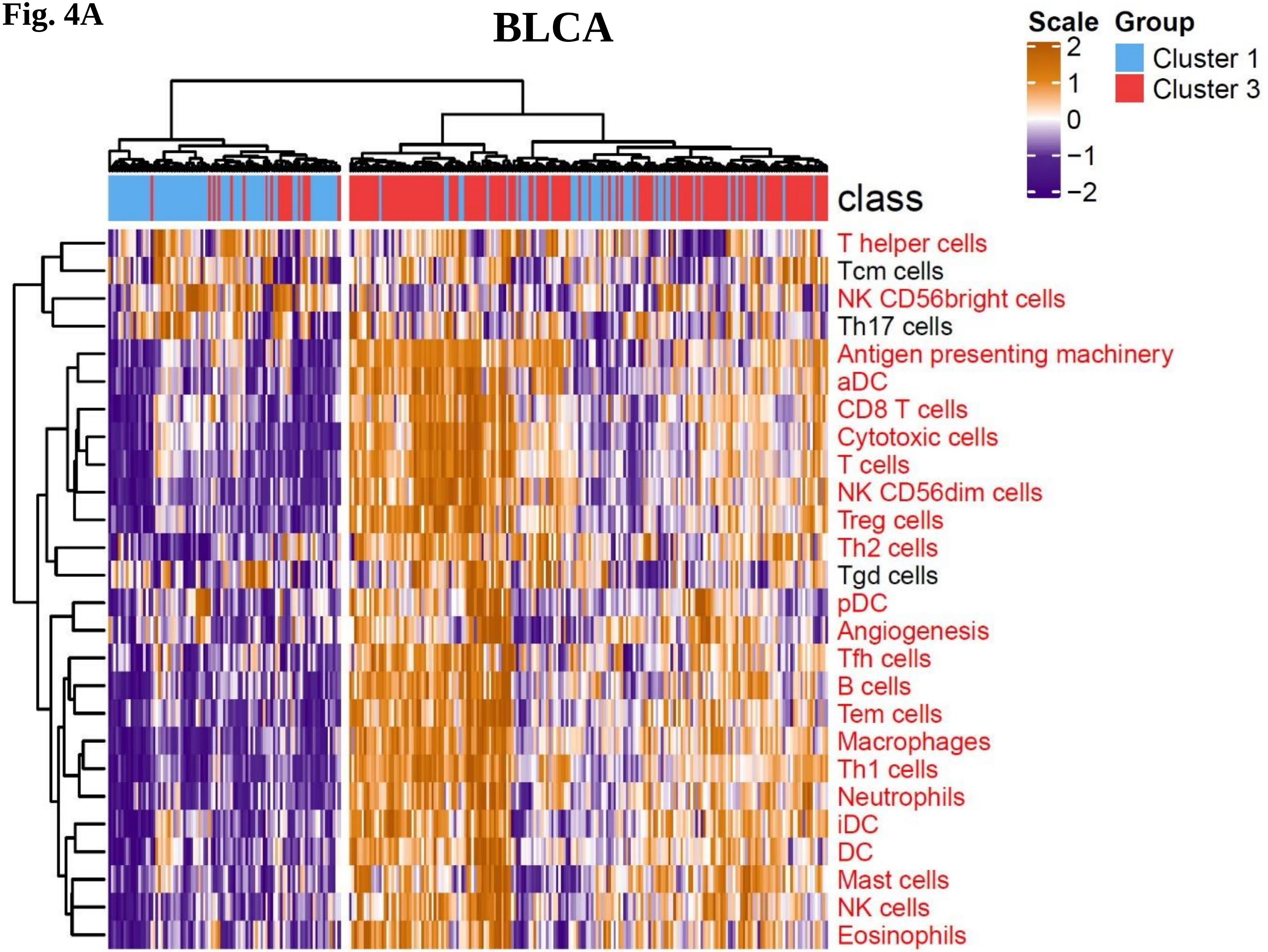
BLCA
Scale
2
1
0
−1
−2
Group
Cluster 1
Cluster 3
class
T helper cells
Tcm cells
NK CD56bright cells
Th17 cells
Antigen presenting machinery
aDC
CD8 T cells
Cytotoxic cells
T cells
NK CD56dim cells
Treg cells
Th2 cells
Tgd cells
pDC
Angiogenesis
Tfh cells
B cells
Tem cells
Macrophages
Th1 cells
Neutrophils
iDC
DC
Mast cells
NK cells
Eosinophils

Fig. 4A

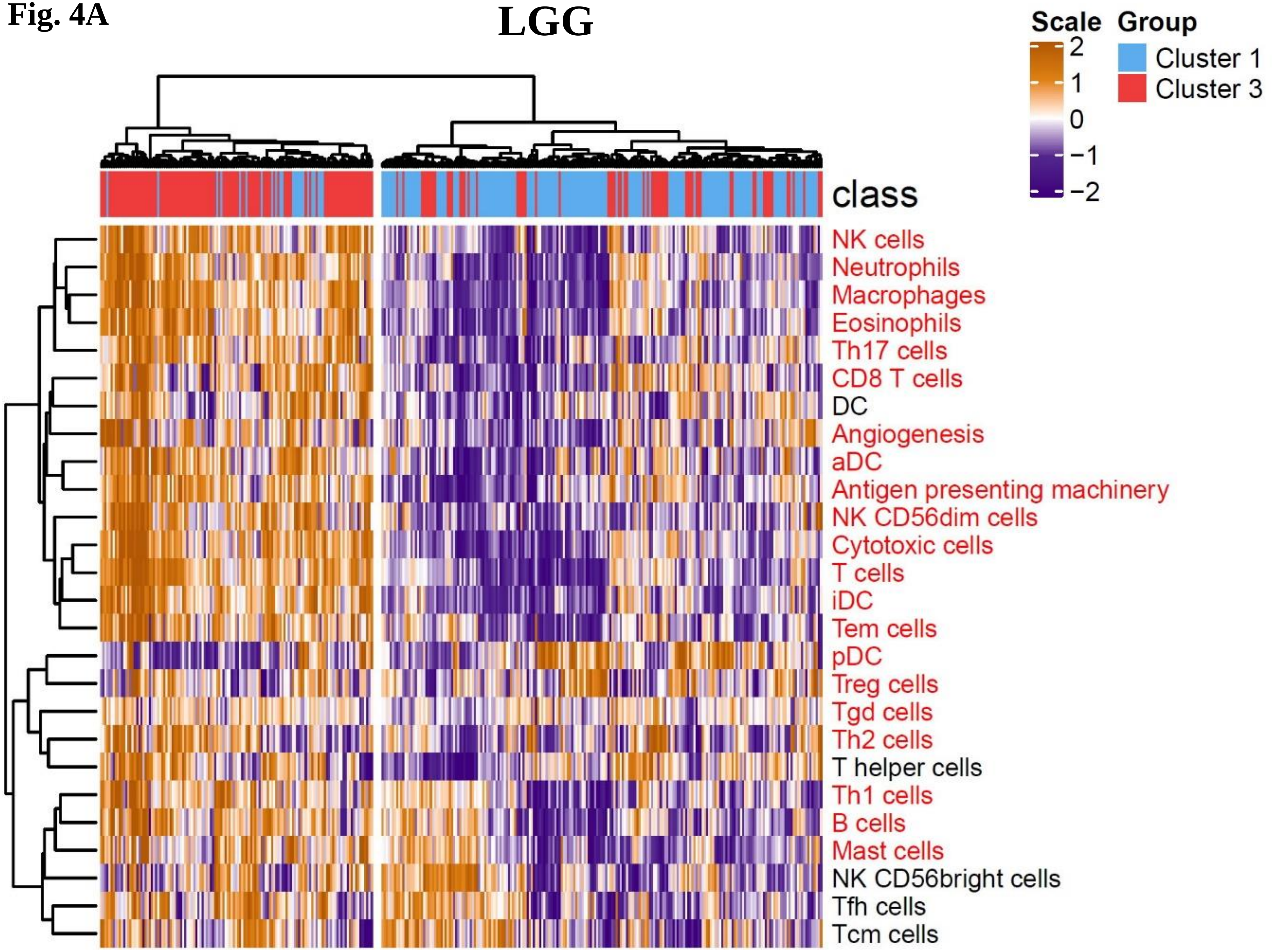
LGG
Scale
2
1
0
−1
−2
Group
Cluster 1
Cluster 3
class
NK cells
Neutrophils
Macrophages
Eosinophils
Th17 cells
CD8 T cells
DC
Angiogenesis
aDC
Antigen presenting machinery
NK CD56dim cells
Cytotoxic cells
T cells
iDC
Tem cells
pDC
Treg cells
Tgd cells
Th2 cells
T helper cells
Th1 cells
B cells
Mast cells
NK CD56bright cells
Tfh cells
Tcm cells

Fig. 4A

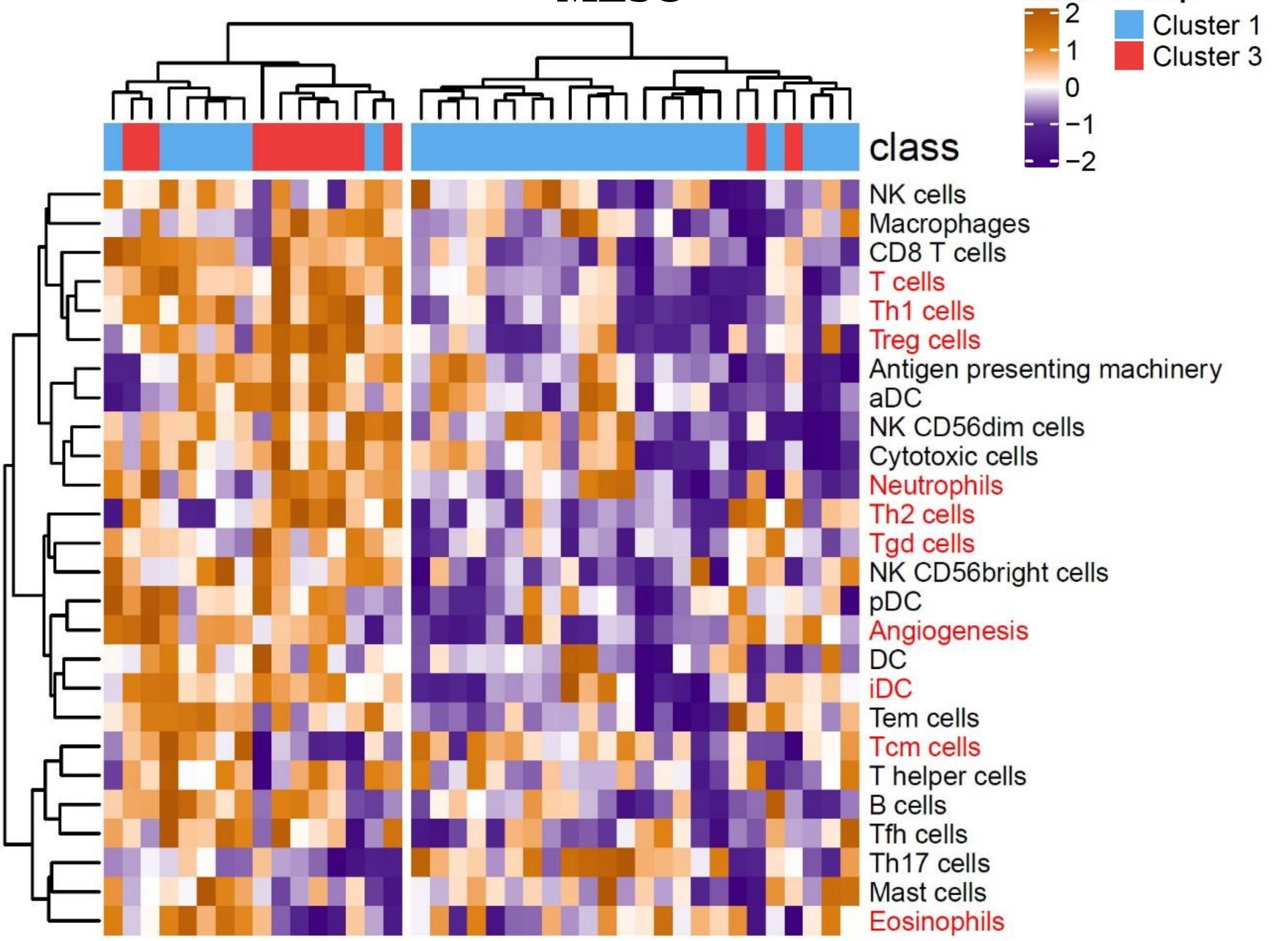
MESO
Scale
2
1
0
−1
−2
Group
Cluster 1
Cluster 3
class
NK cells
Macrophages
CD8 T cells
T cells
Th1 cells
Treg cells
Antigen presenting machinery
aDC
NK CD56dim cells
Cytotoxic cells
Neutrophils
Th2 cells
Tgd cells
NK CD56bright cells
pDC
Angiogenesis
DC
iDC
Tem cells
Tcm cells
T helper cells
B cells
Tfh cells
Th17 cells
Mast cells
Eosinophils

Fig. 4A

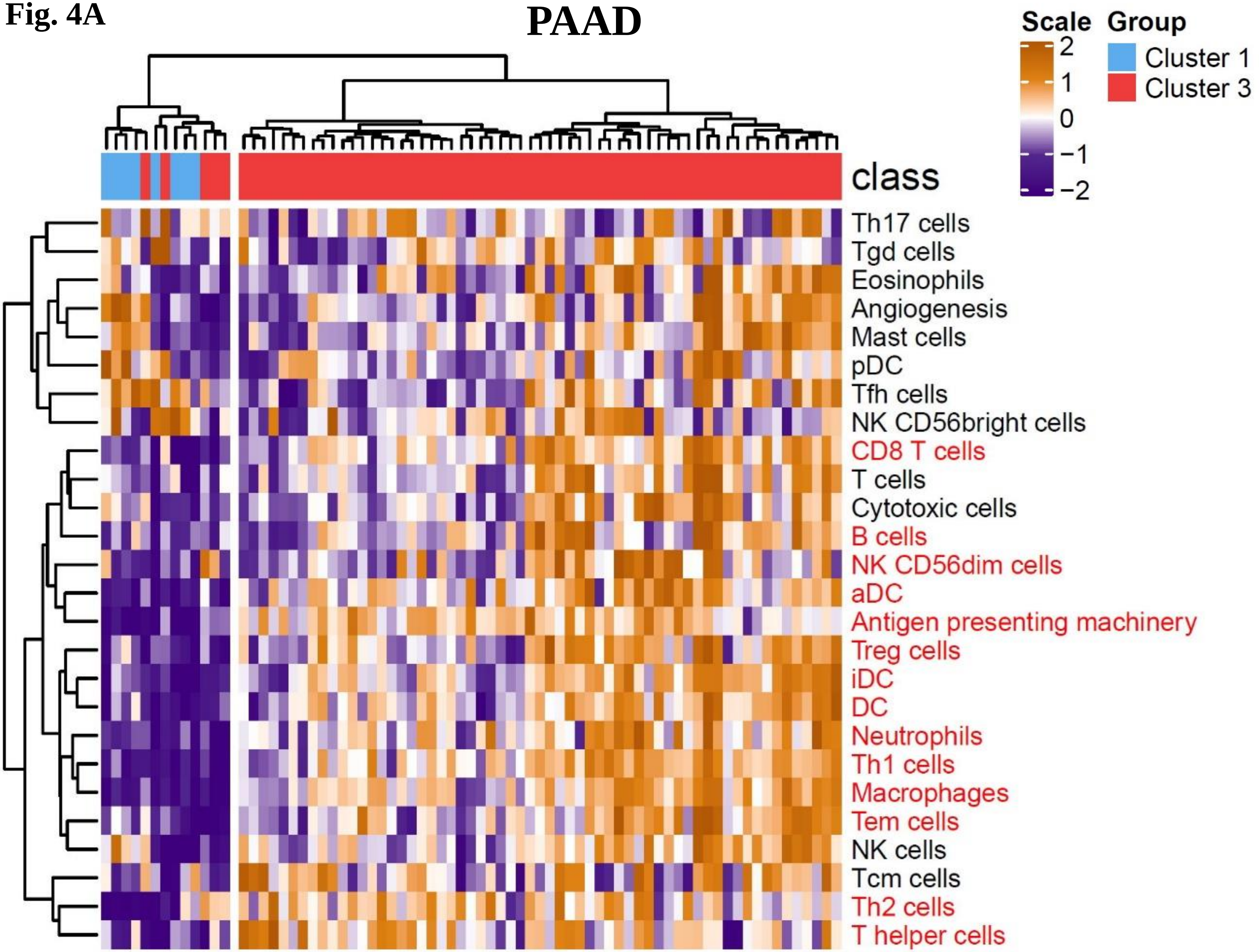
PAAD
Scale
2
1
0
−1
−2
Group
Cluster 1
Cluster 3
class
Th17 cells
Tgd cells
Eosinophils
Angiogenesis
Mast cells
pDC
Tfh cells
NK CD56bright cells
CD8 T cells
T cells
Cytotoxic cells
B cells
NK CD56dim cells
aDC
Antigen presenting machinery
Treg cells
iDC
DC
Neutrophils
Th1 cells
Macrophages
Tem cells
NK cells
Tcm cells
Th2 cells
T helper cells

Fig. 4A

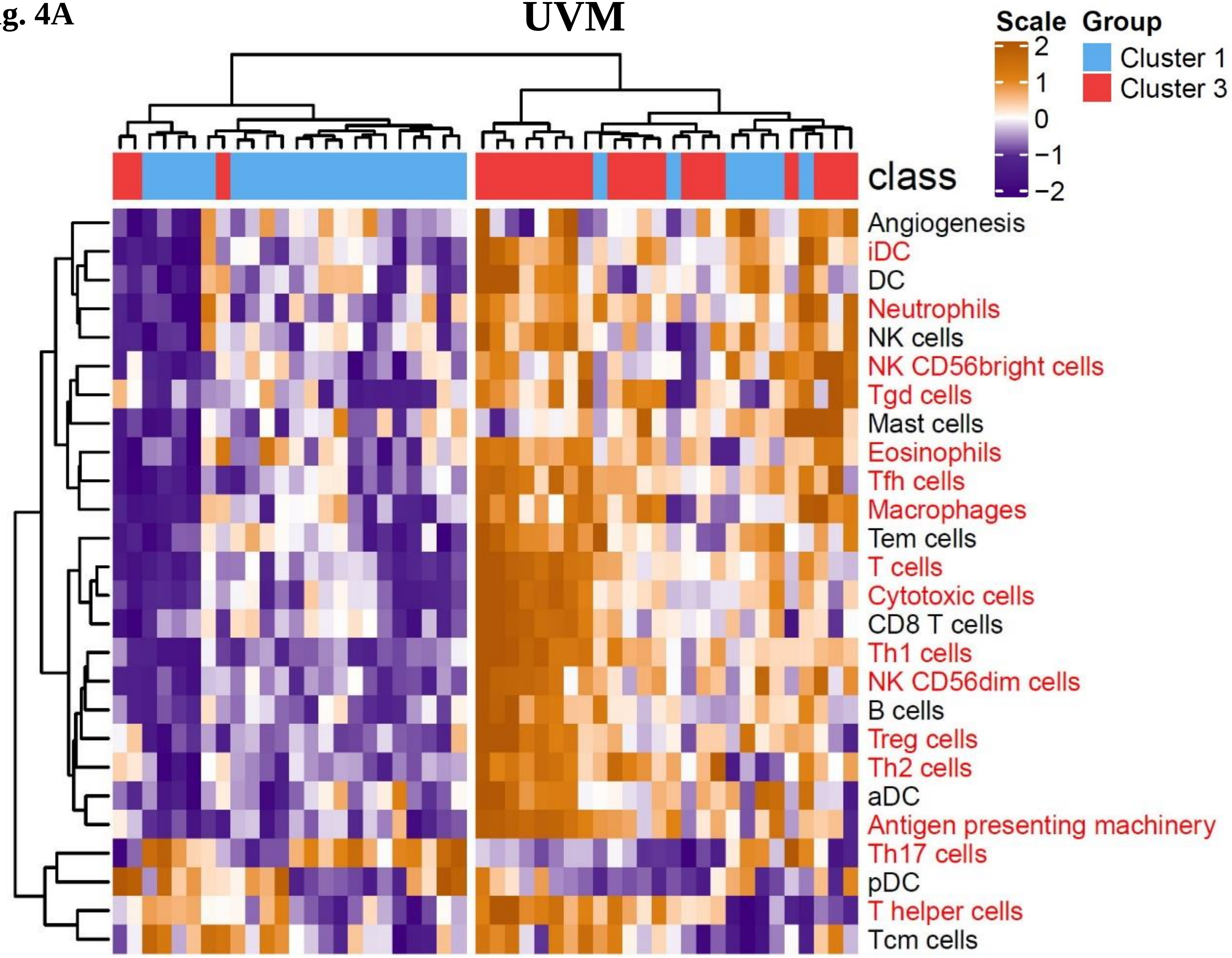
UVM
Scale
2
1
0
−1
−2
Group
Cluster 1
Cluster 3
class
Angiogenesis
iDC
DC
Neutrophils
NK cells
NK CD56bright cells
Tgd cells
Mast cells
Eosinophils
Tfh cells
Macrophages
Tem cells
T cells
Cytotoxic cells
CD8 T cells
Th1 cells
NK CD56dim cells
B cells
Treg cells
Th2 cells
aDC
Antigen presenting machinery
Th17 cells
pDC
T helper cells
Tcm cells

Fig. 4B

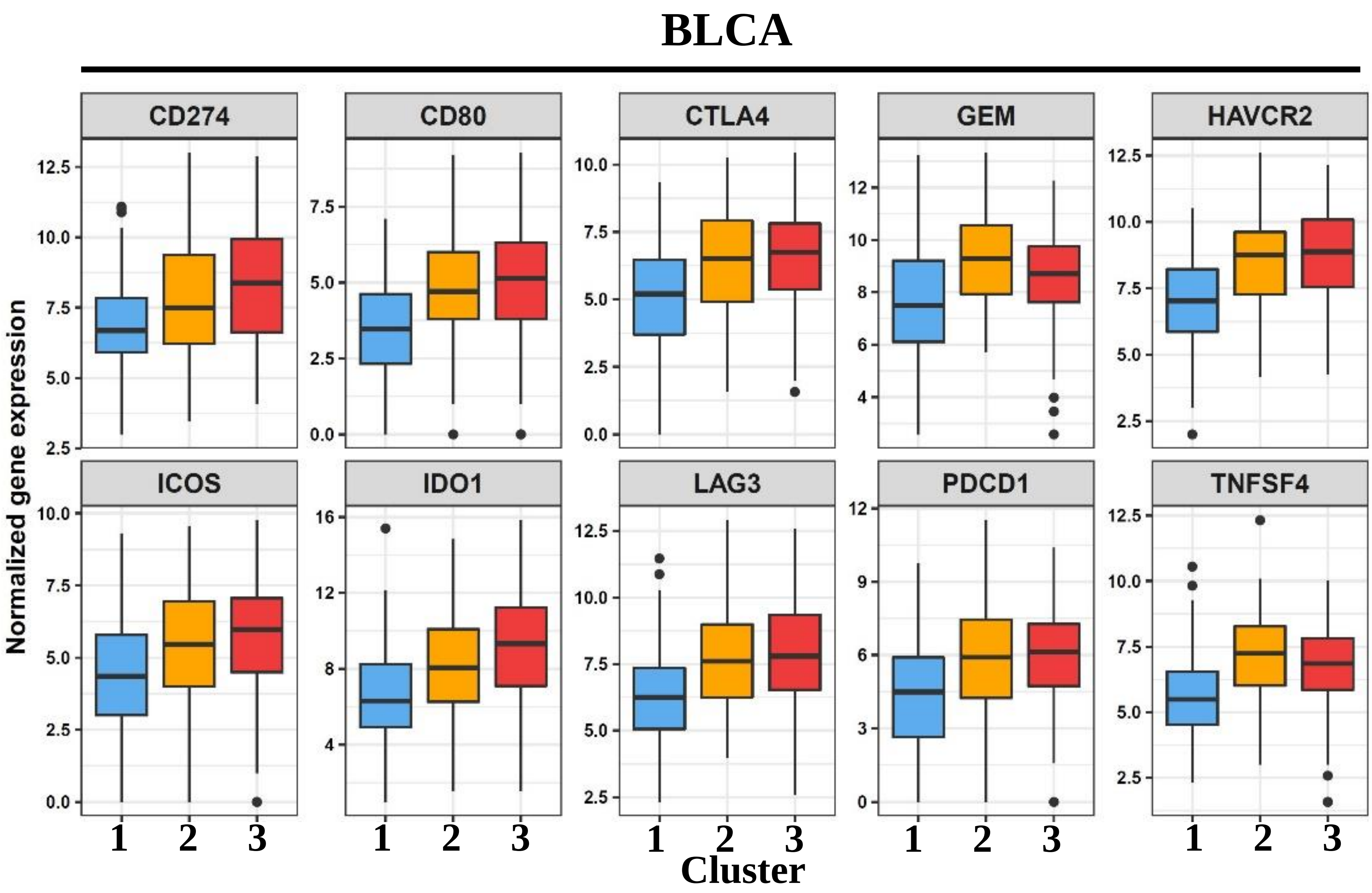

BLCA
CD274
CD80
CTLA4
GEM
HAVCR2
ICOS
IDO1
LAG3
PDCD1
TNFSF4
Normalized gene expression
Cluster
1
2
3

Fig. 4B

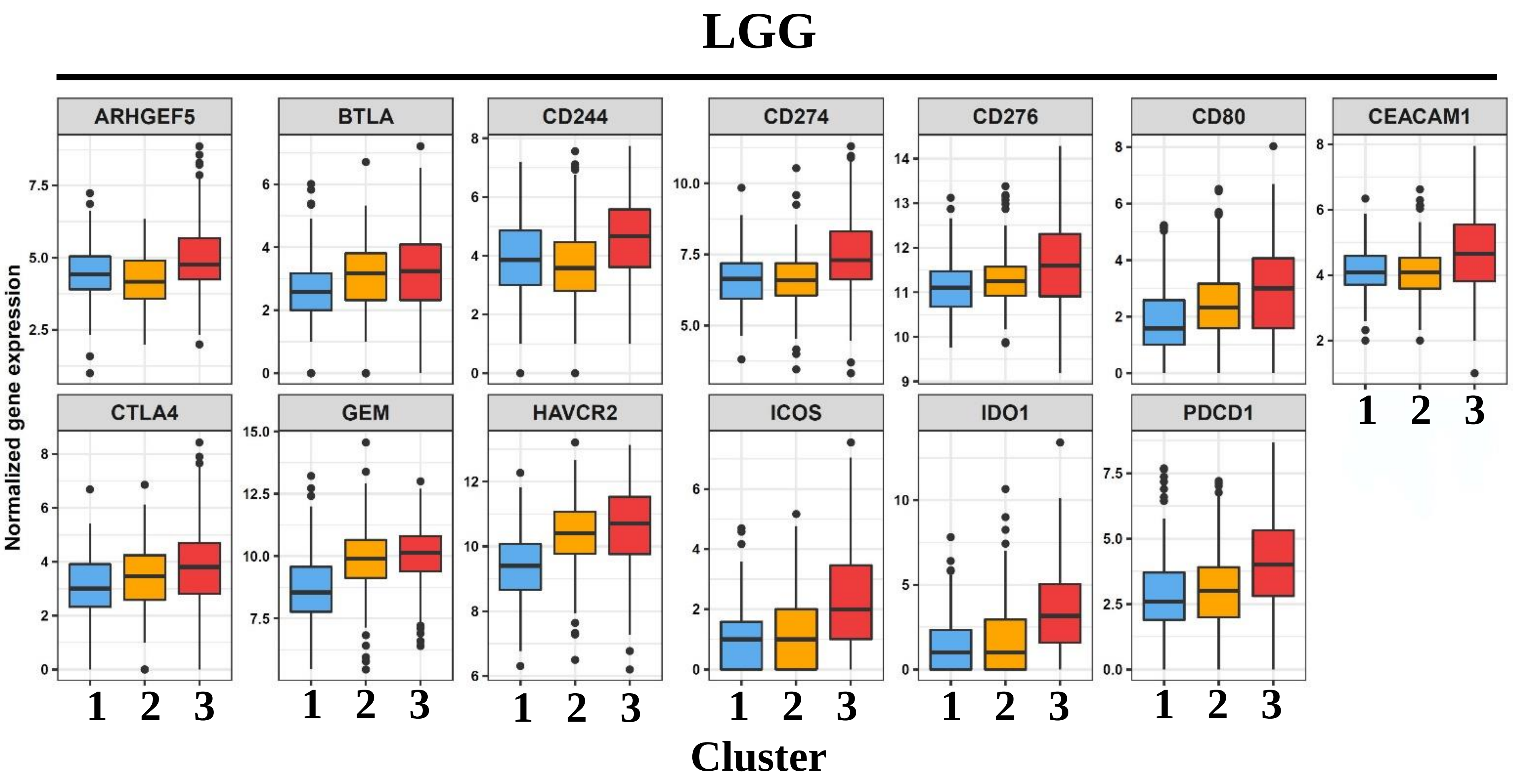
LGG
ARHGEF5
BTLA
CD244
CD274
CD276
CD80
CEACAM1
CTLA4
GEM
HAVCR2
ICOS
IDO1
PDCD1
Normalized gene expression
1 2 3
Cluster

Fig. 4B

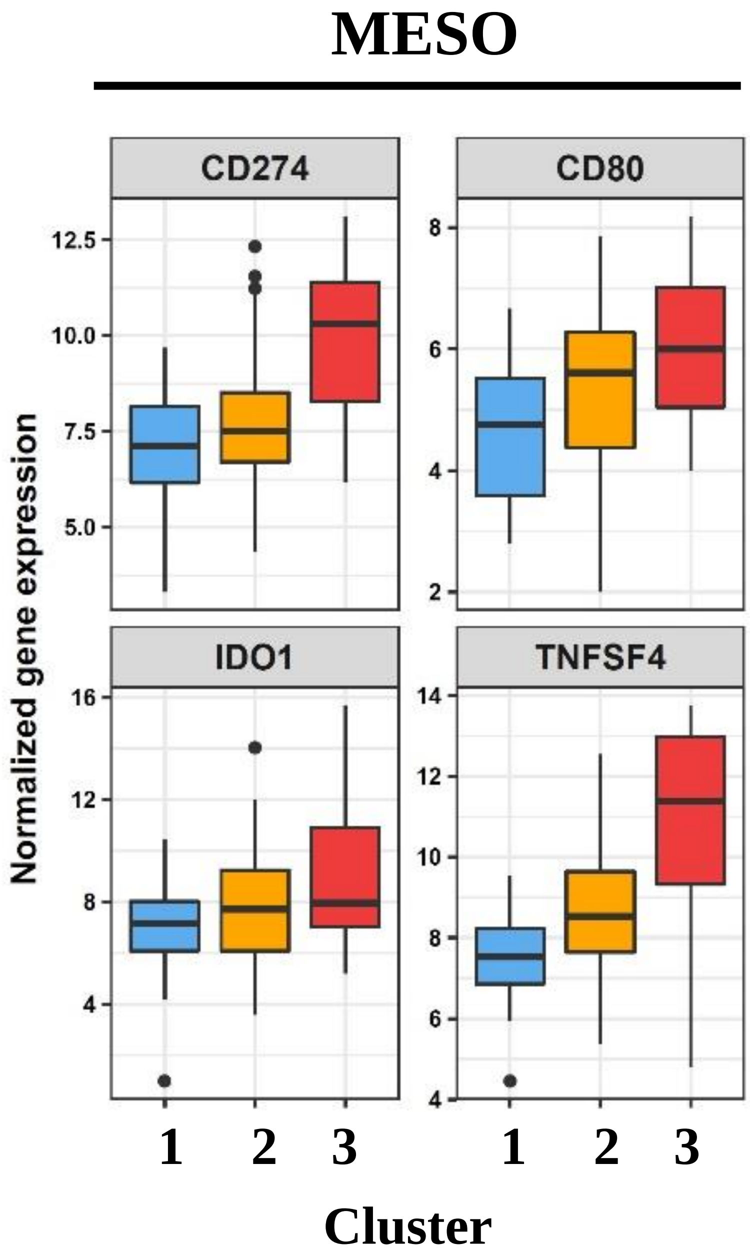
MESO
CD274
CD80
IDO1
TNFSF4
Normalized gene expression
12.5
10.0
7.5
5.0
8
6
4
2
16
12
8
4
14
12
10
8
6
4
1 2 3
1 2 3
Cluster

Fig. 4B

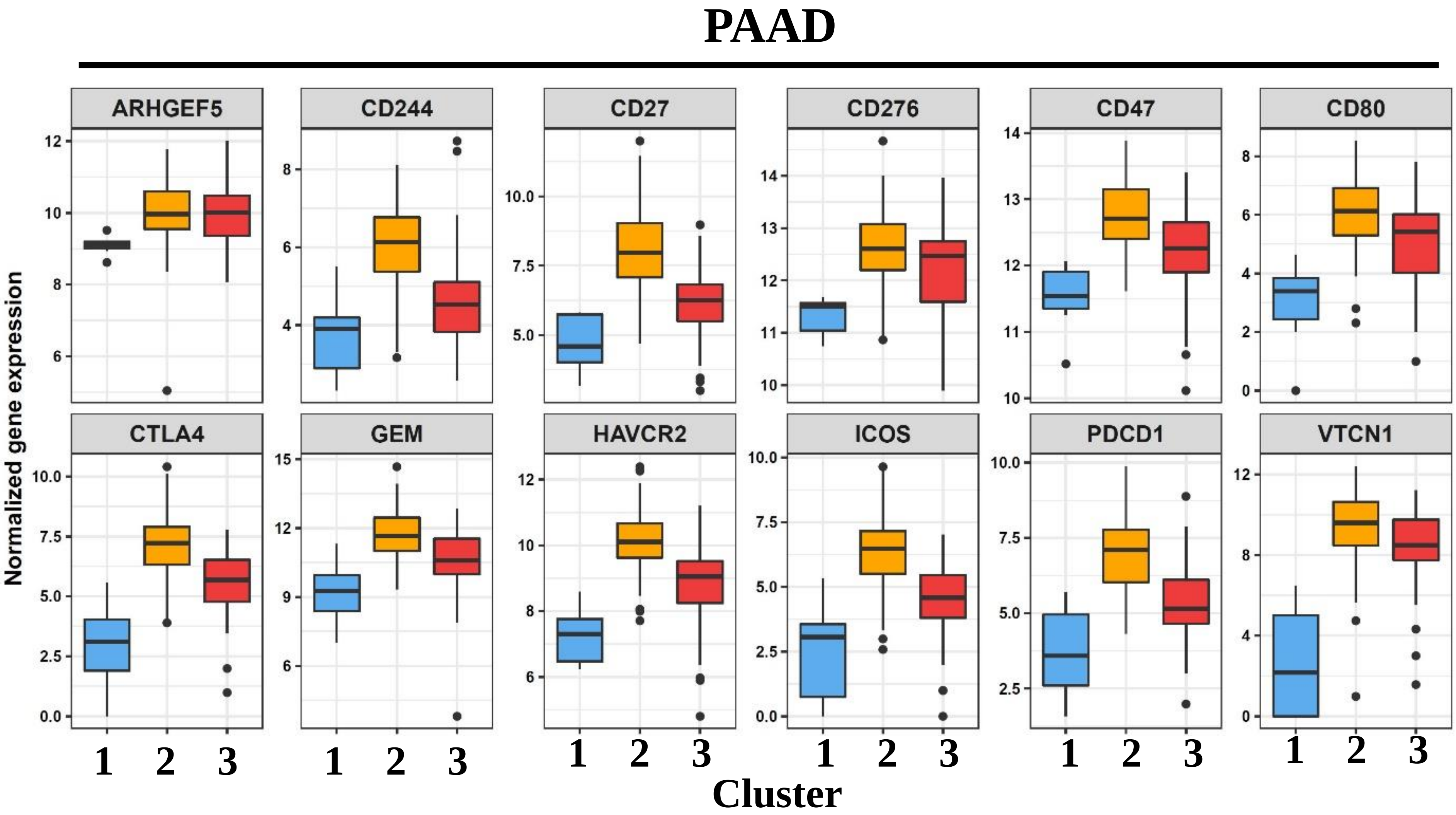
PAAD
ARHGEF5
CD244
CD27
CD276
CD47
CD80
CTLA4
GEM
HAVCR2
ICOS
PDCD1
VTCN1
Normalized gene expression
1 2 3
Cluster

Fig. 4B

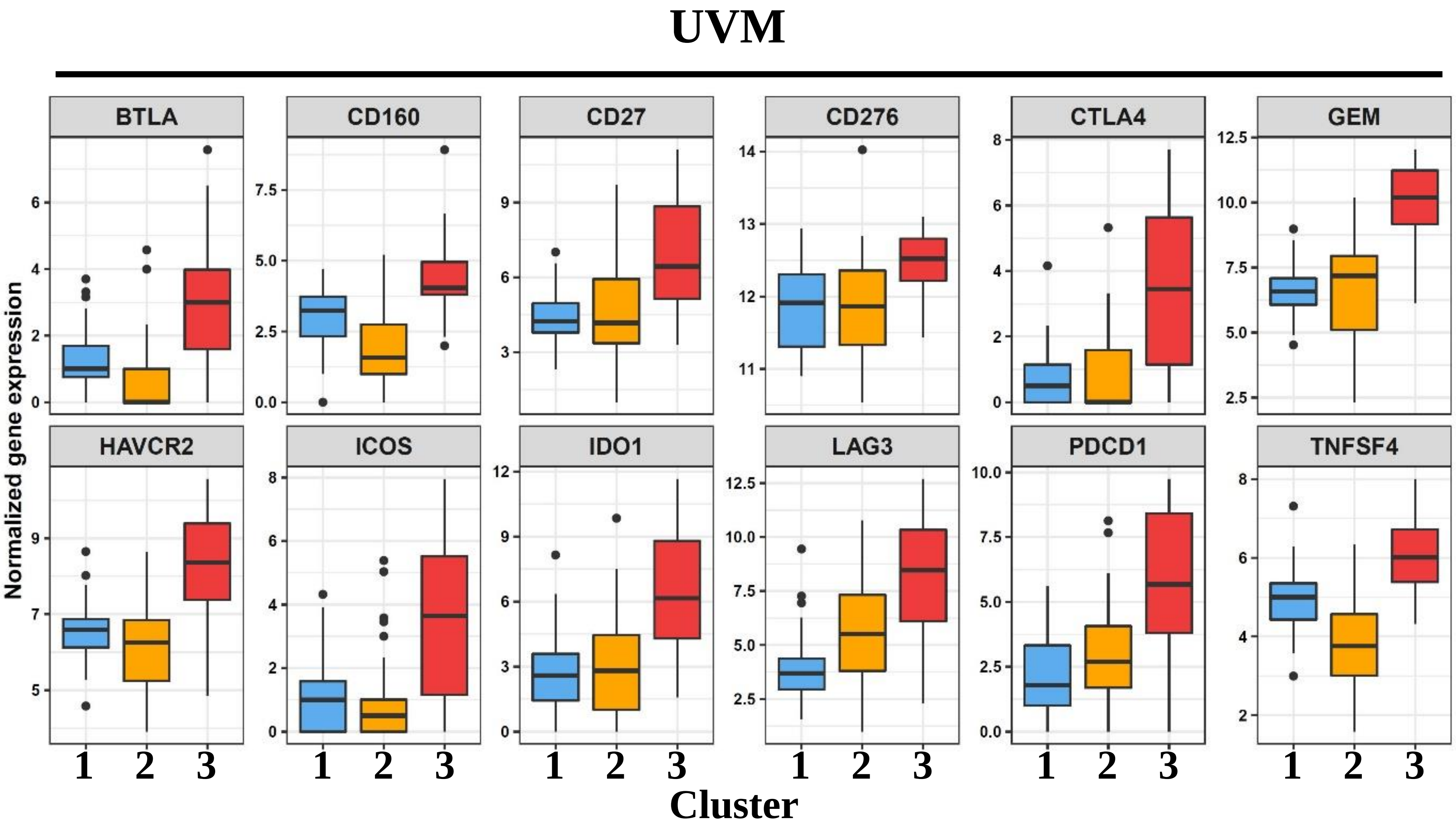
UVM
BTLA
CD160
CD27
CD276
CTLA4
GEM
HAVCR2
ICOS
IDO1
LAG3
PDCD1
TNFSF4
Normalized gene expression
1 2 3
Cluster

Fig. 4C

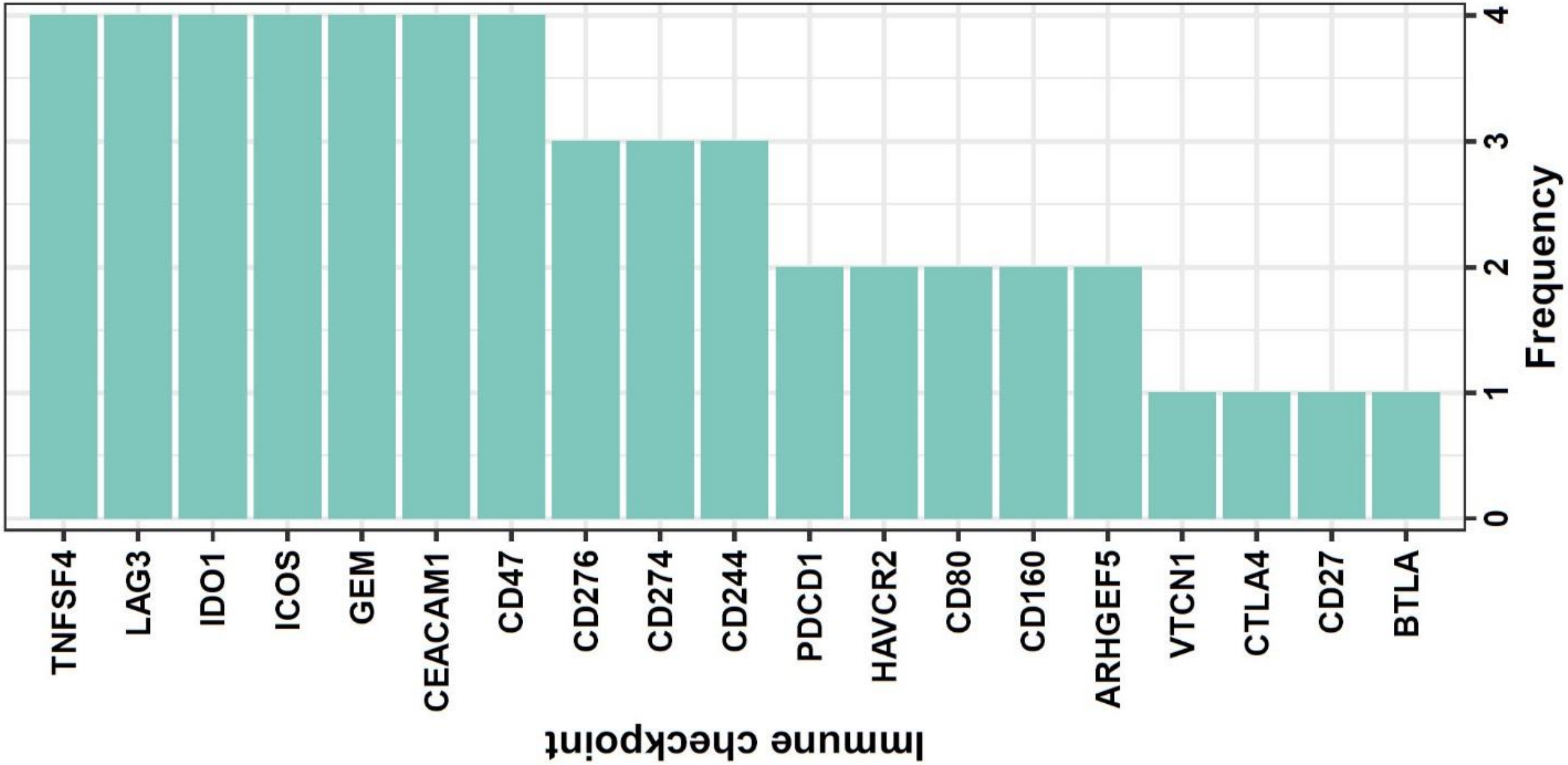
Frequency
0
1
2
3
4
TNFSF4
LAG3
IDO1
ICOS
GEM
CEACAM1
CD47
CD276
CD274
CD244
PDCD1
HAVCR2
CD80
CD160
ARHGEF5
VTCN1
CTLA4
CD27
BTLA
Immune checkpoint

Fig. 5A

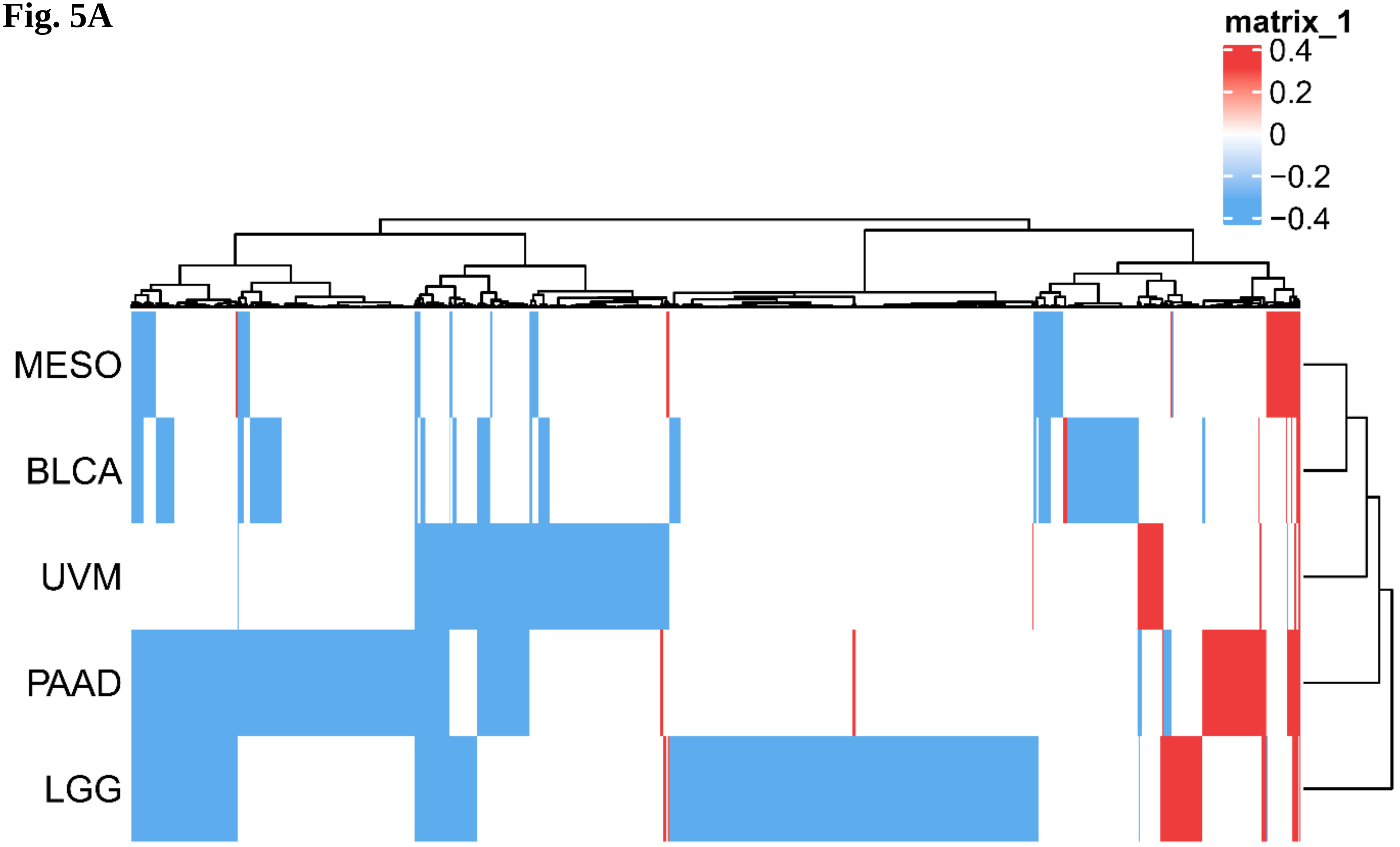

Fig. 5B

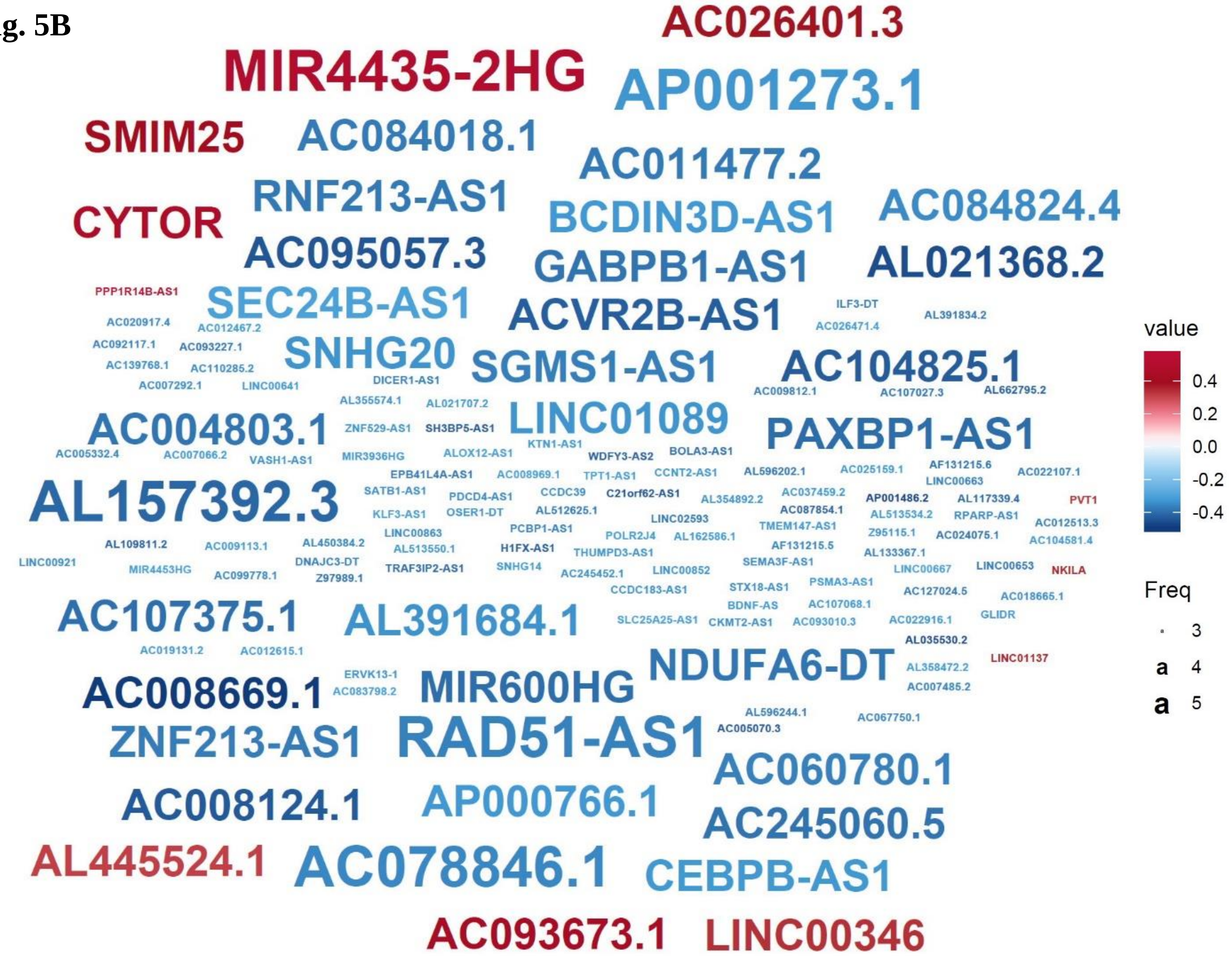
AC026401.3
MIR4435-2HG
AP001273.1
SMIM25
AC084018.1
AC011477.2
RNF213-AS1
CYTOR
BCDIN3D-AS1
AC084824.4
AC095057.3
GABPB1-AS1
AL021368.2
SEC24B-AS1
ACVR2B-AS1
SNHG20
SGMS1-AS1
AC104825.1
LINC01089
PAXBP1-AS1
AC004803.1
AL157392.3
AC107375.1
AL391684.1
NDUFA6-DT
AC008669.1
MIR600HG
ZNF213-AS1
RAD51-AS1
AC060780.1
AC008124.1
AP000766.1
AC245060.5
AL445524.1
AC078846.1
CEBPB-AS1
AC093673.1
LINC00346
value
0.4
0.2
0.0
-0.2
-0.4
Freq
3
4
5

Fig. 5C

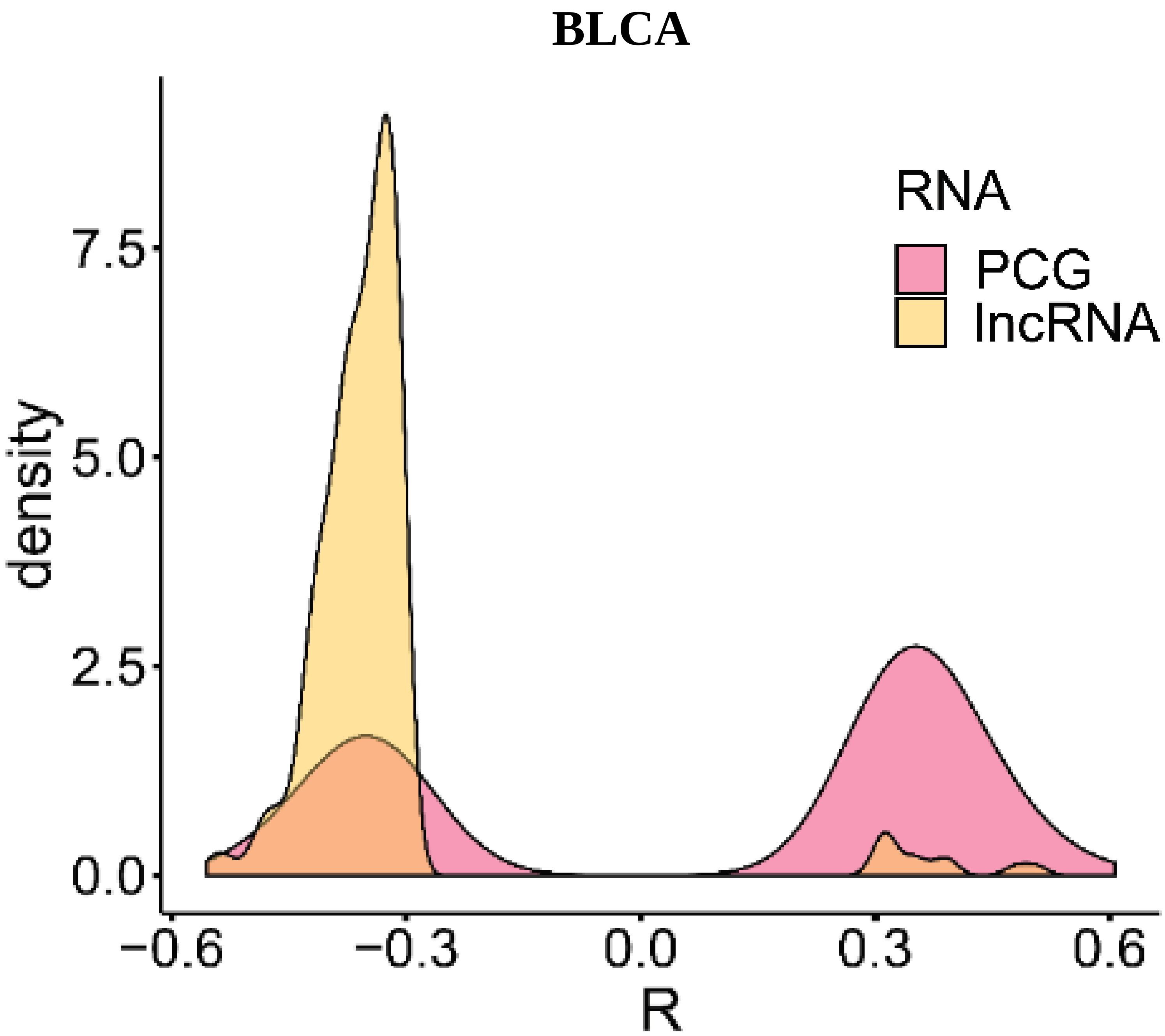

Fig. 5C

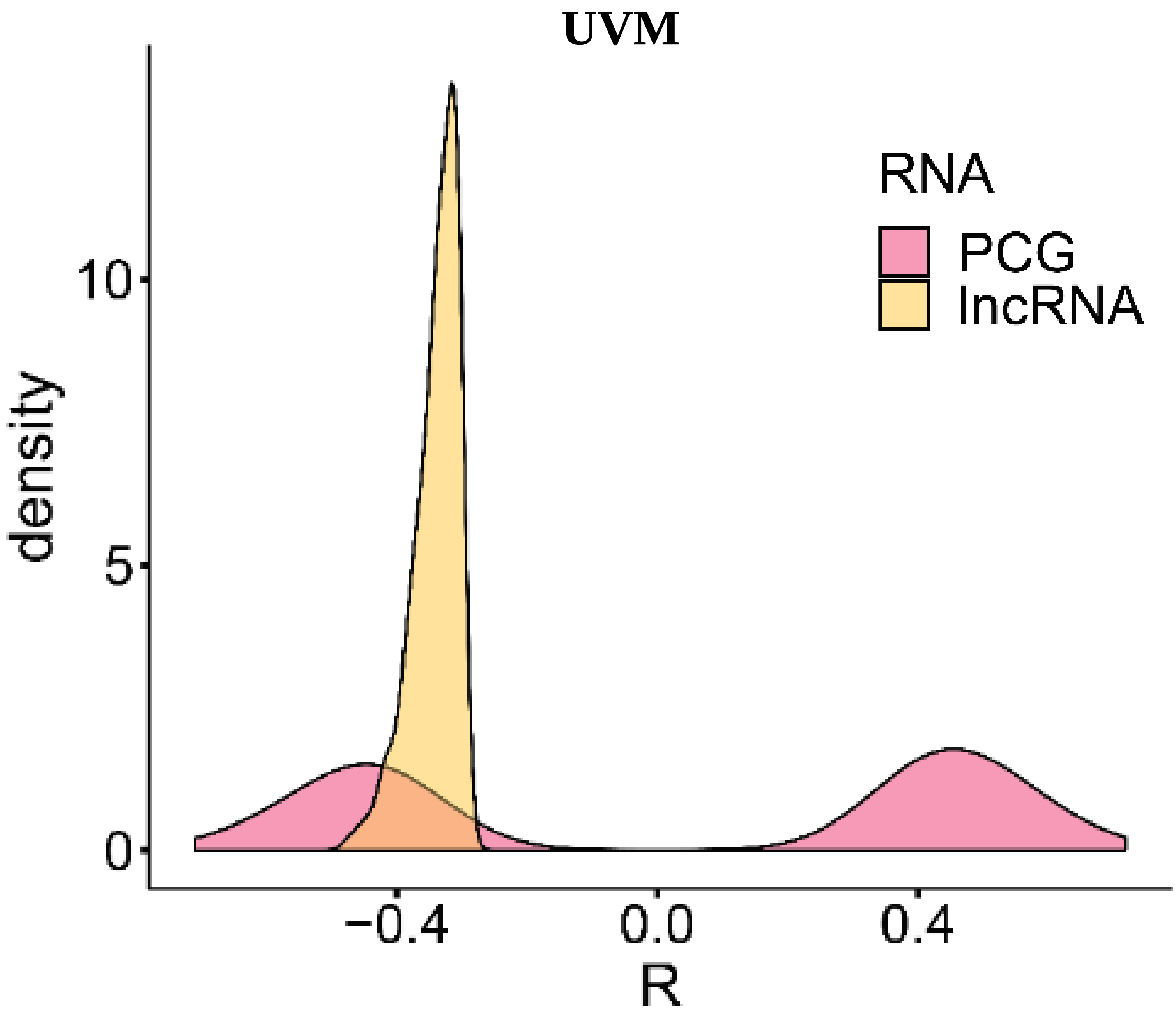

Fig. 5C

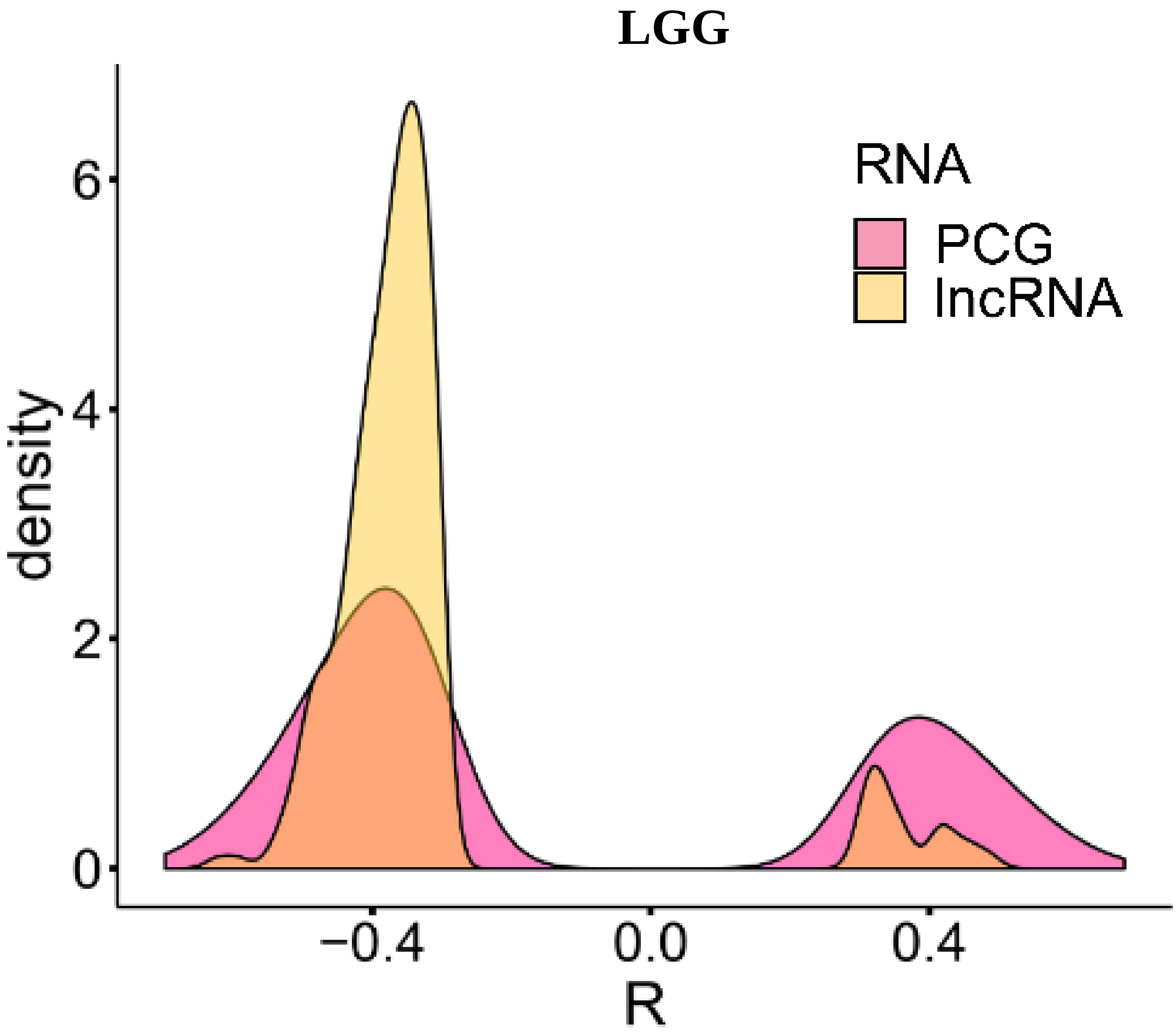

Fig. 5C

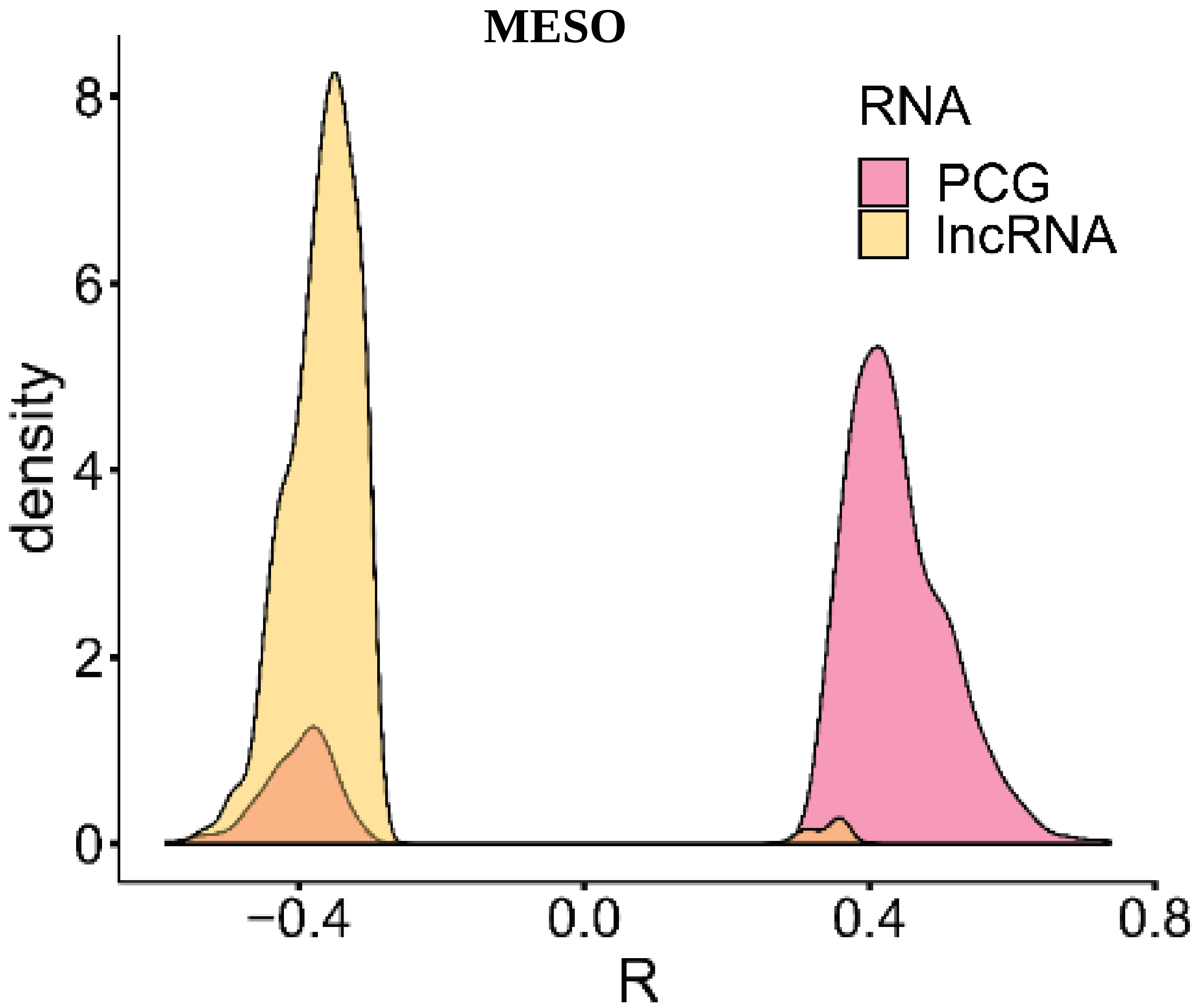

Fig. 5C

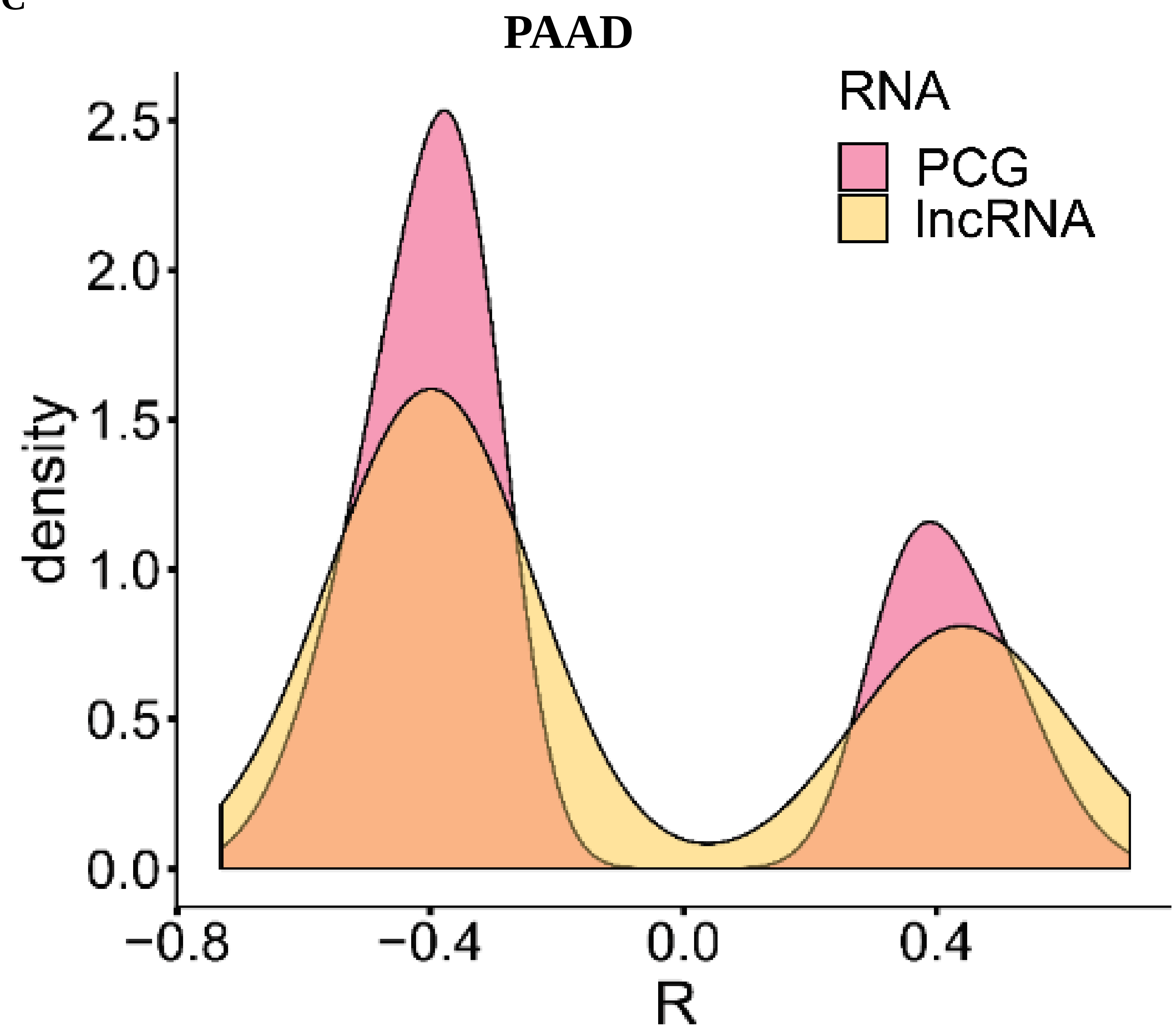

Fig. 5D

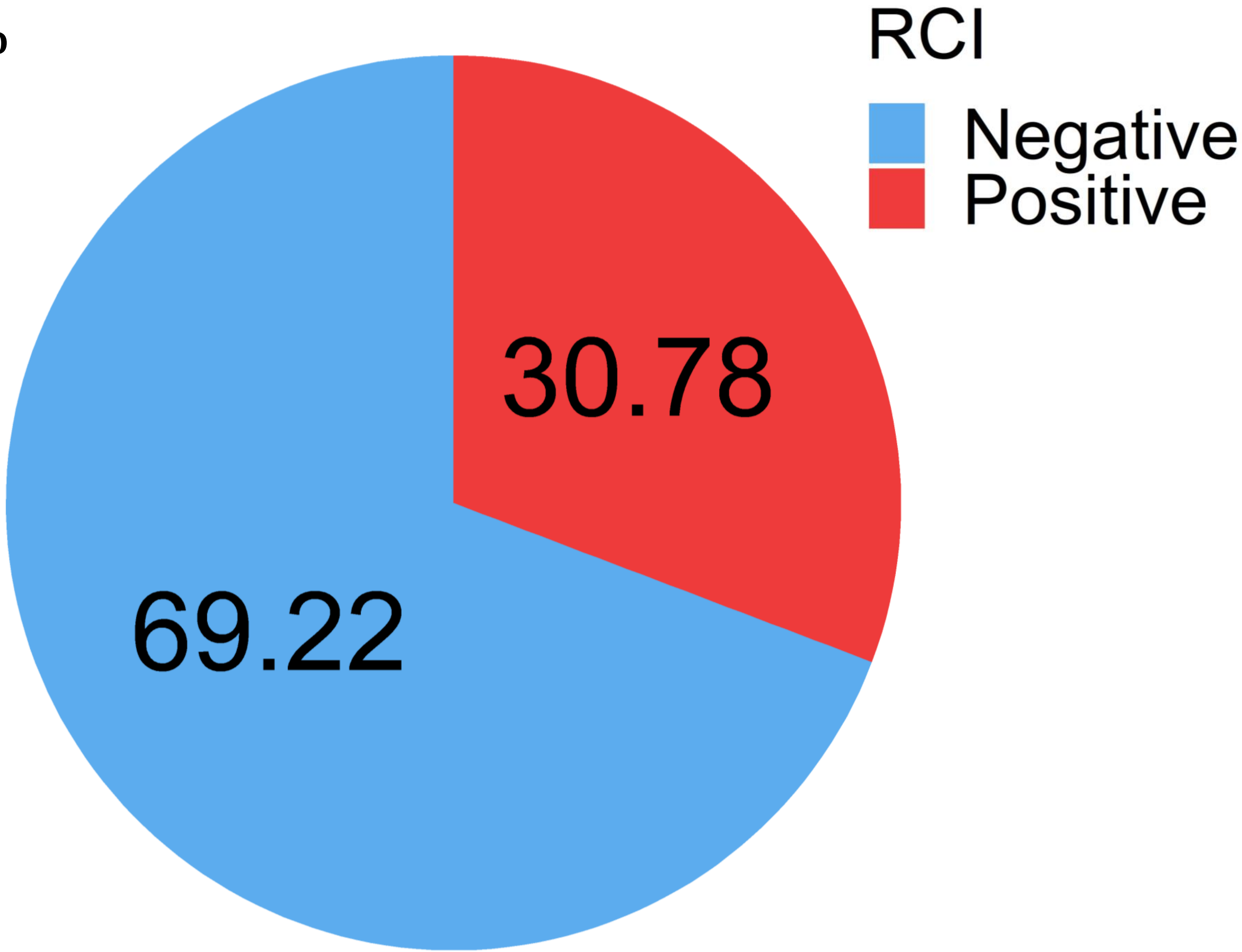

Fig. 5E

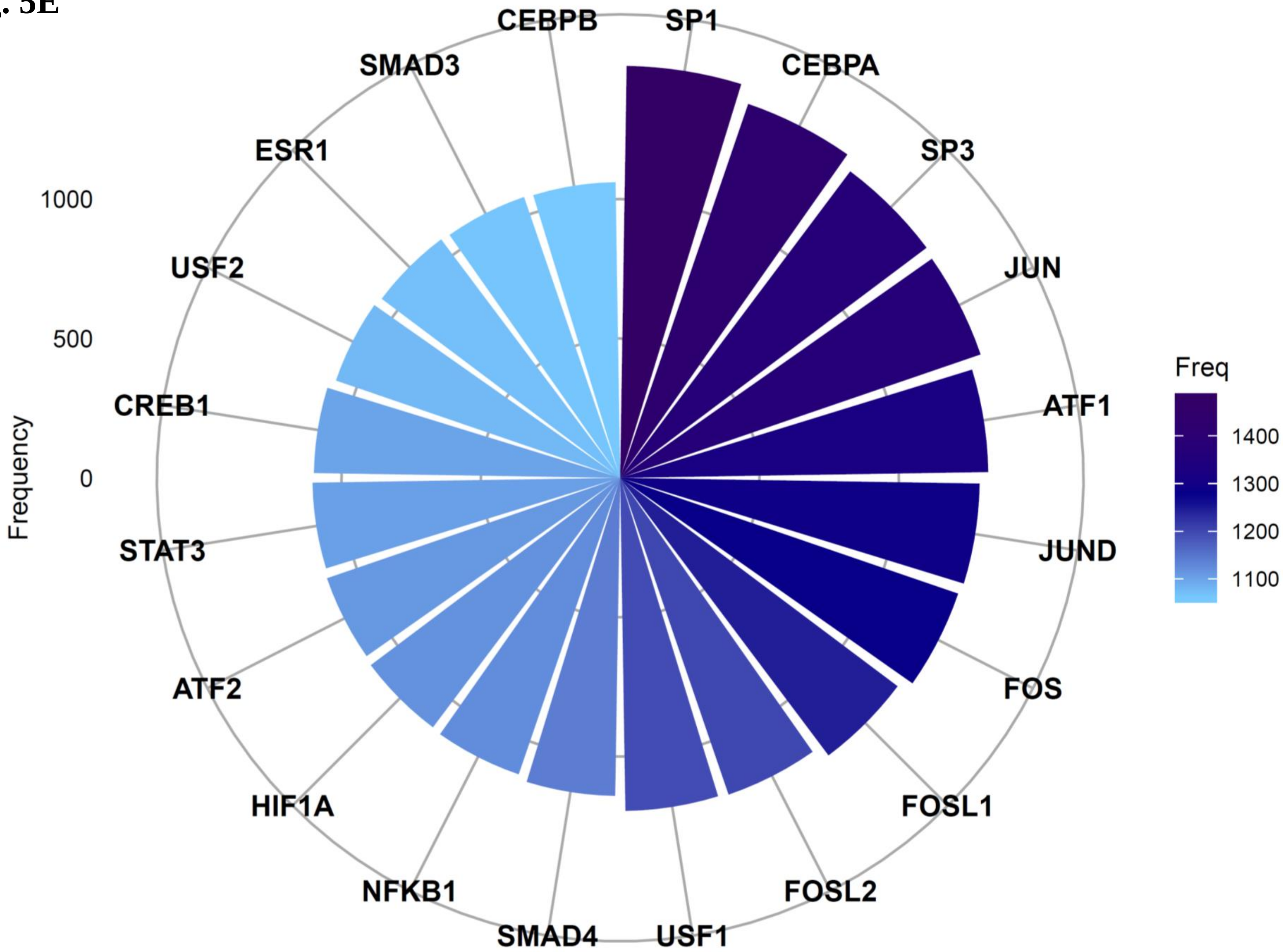

CEBPB
SP1
CEBPA
SMAD3
ESR1
SP3
1000
USF2
JUN
500
Freq
CREB1
ATF1
1400
Frequency
0
1300
STAT3
JUND
1200
1100
ATF2
FOS
HIF1A
FOSL1
NFKB1
FOSL2
SMAD4
USF1

Fig. 5F

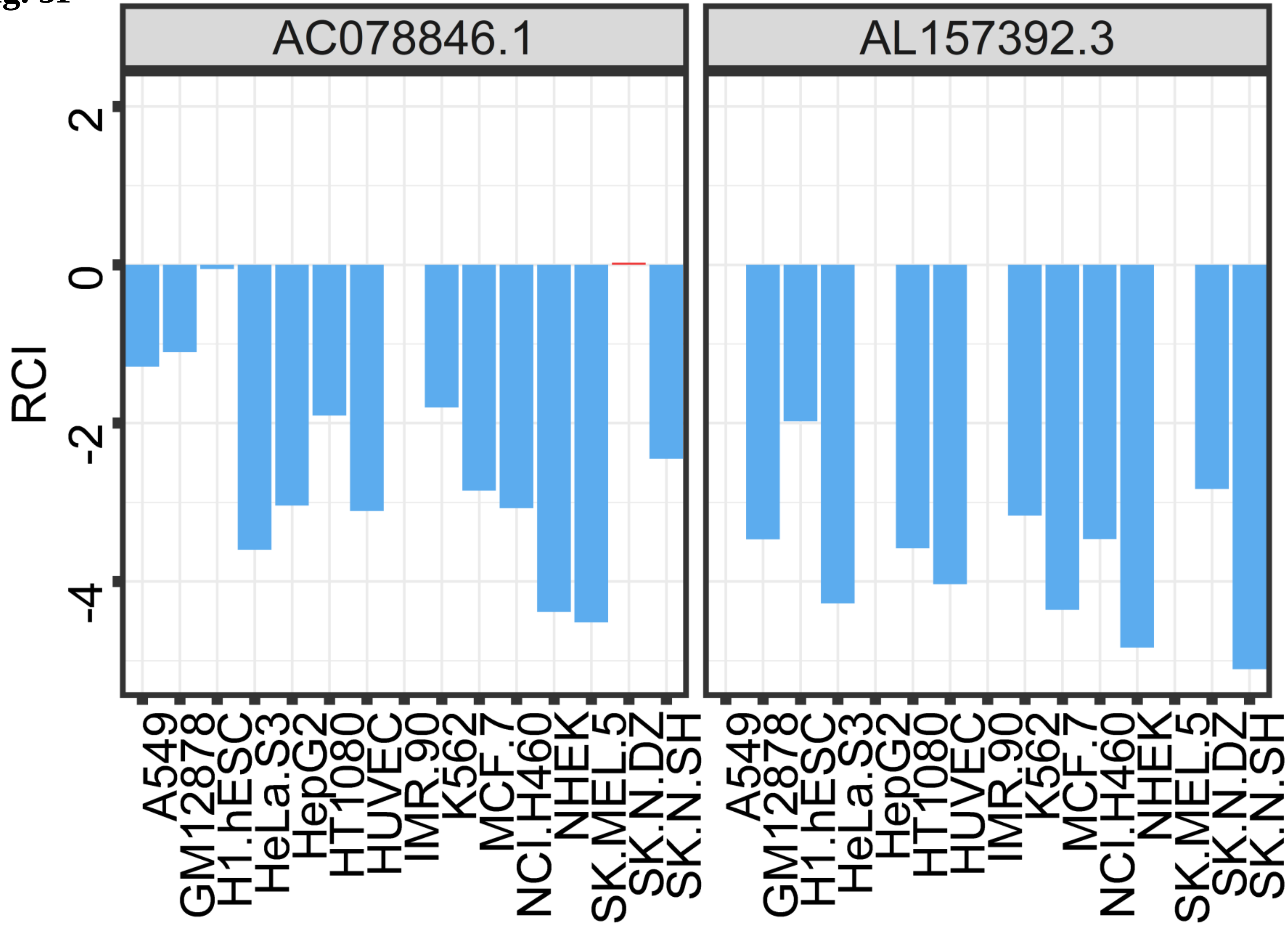

AC078846.1
AL157392.3
RCI
2
0
-2
-4
A549
GM12878
H1.hESC
HeLa.S3
HepG2
HT1080
HUVEC
IMR.90
K562
MCF.7
NCI.H460
NHEK
SK.MEL.5
SK.N.DZ
SK.N.SH

Fig. 5F

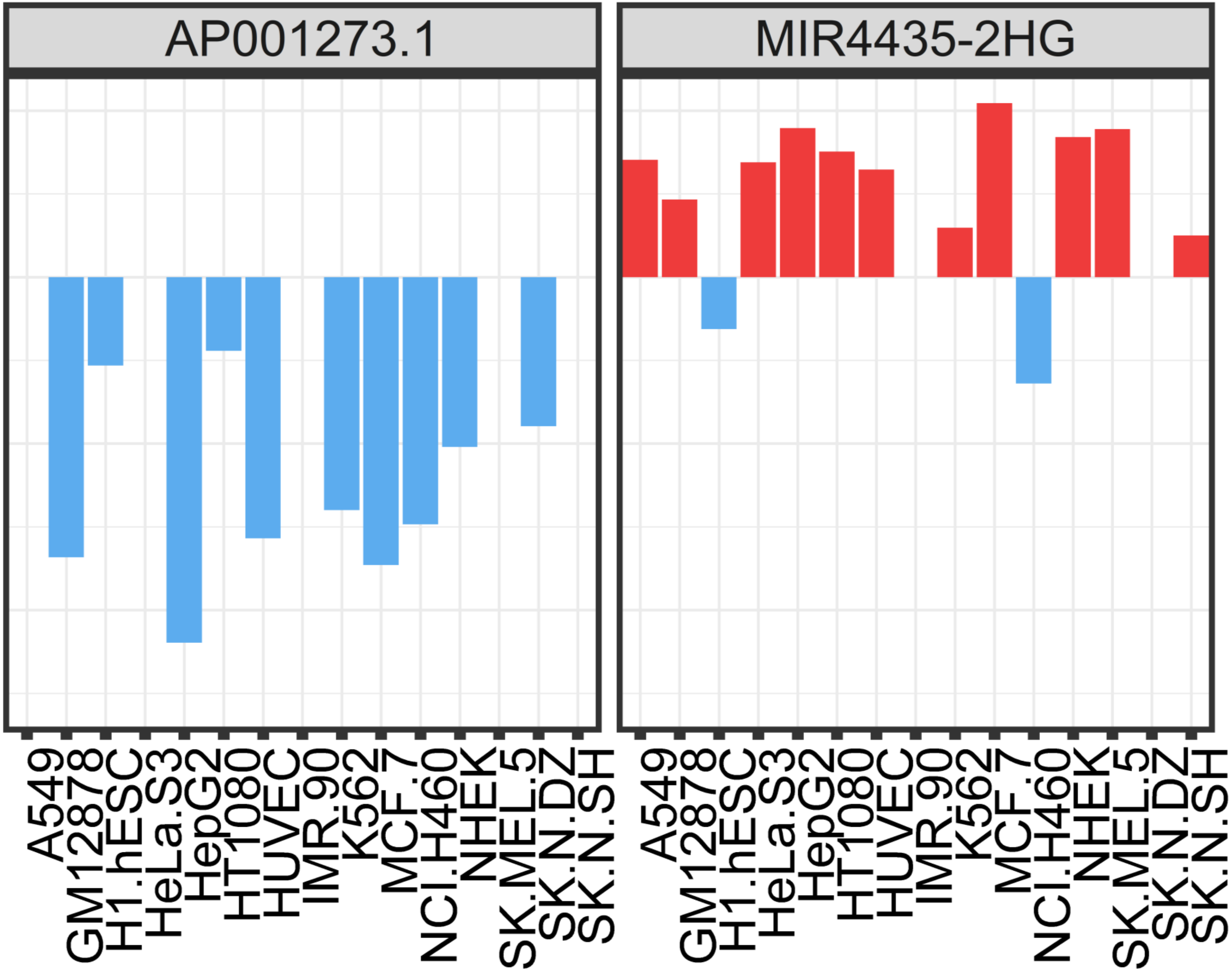

AP001273.1
MIR4435-2HG
A549
GM12878
H1.hESC
HeLa.S3
HepG2
HT1080
HUVEC
IMR.90
K562
MCF.7
NCI.H460
NHEK
SK.MEL.5
SK.N.DZ
SK.N.SH

Fig. 5F

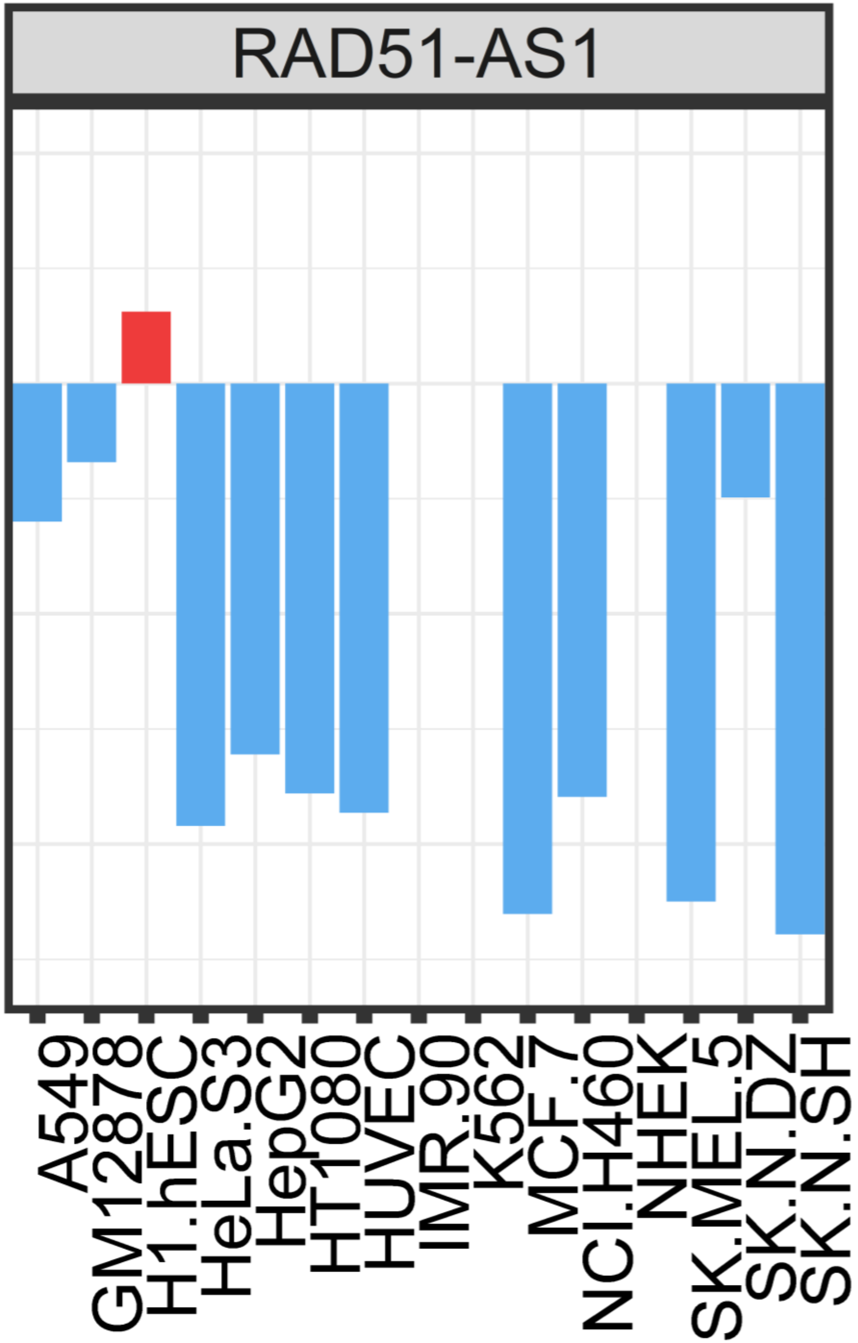

RAD51-AS1
A549
GM12878
H1.hESC
HeLa.S3
HepG2
HT1080
HUVEC
IMR.90
K562
MCF.7
NCI.H460
NHEK
SK.MEL.5
SK.N.DZ
SK.N.SH

Fig. 5G

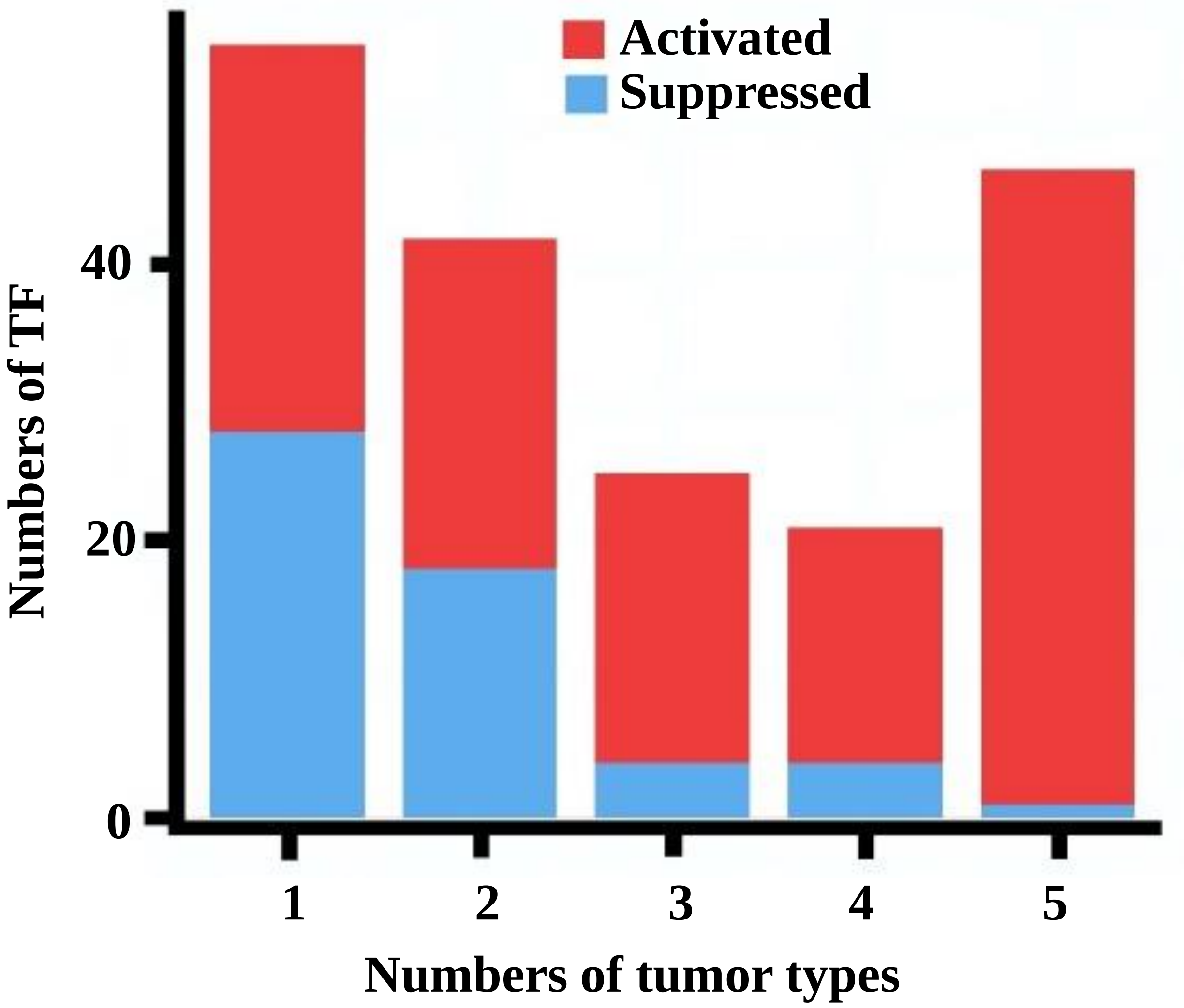

Activated
Suppressed
Numbers of TF
40
20
0
1
2
3
4
5
Numbers of tumor types

Fig. 5H

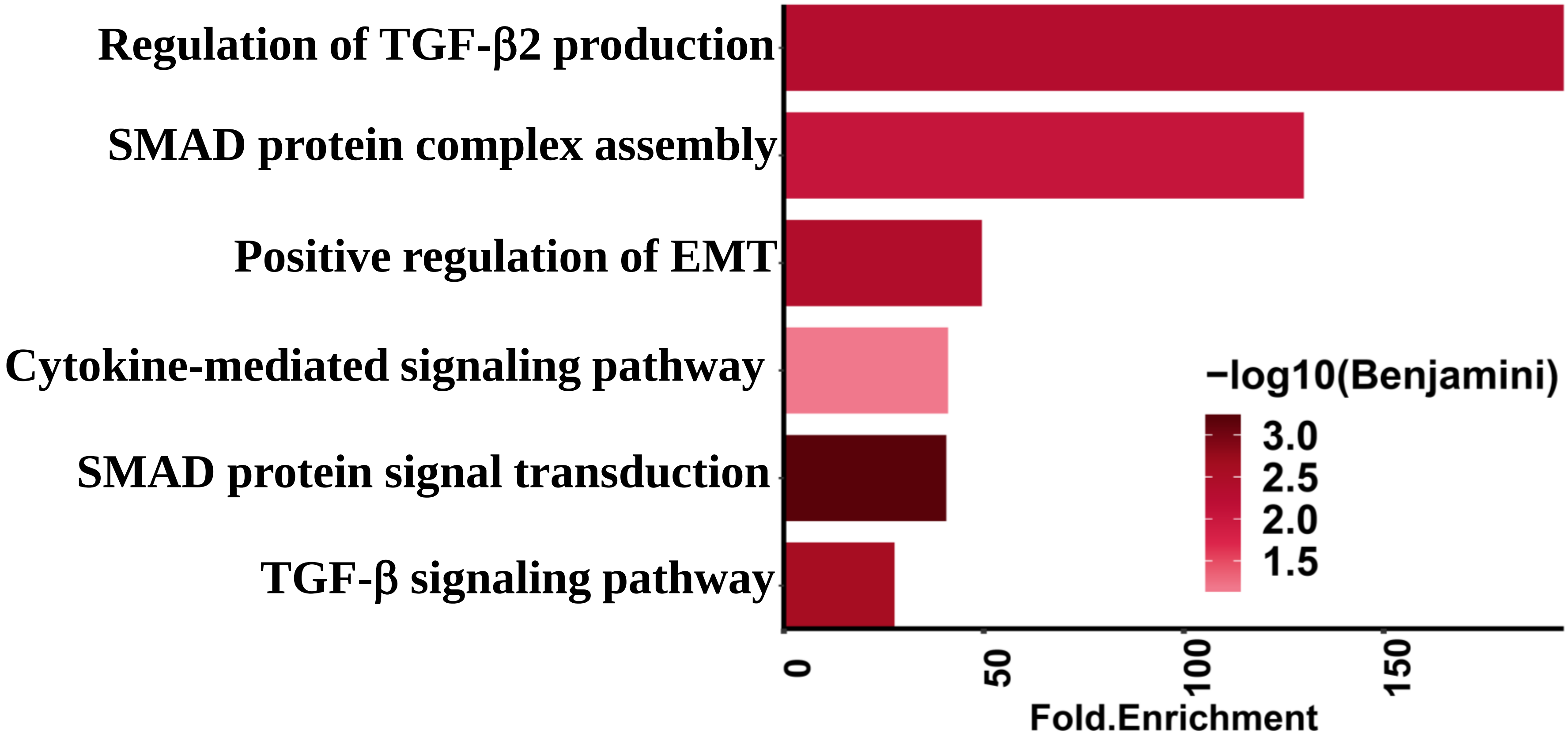

Regulation of TGF-β2 production
SMAD protein complex assembly
Positive regulation of EMT
Cytokine-mediated signaling pathway
SMAD protein signal transduction
TGF-β signaling pathway
−log10(Benjamini)
3.0
2.5
2.0
1.5
0
50
100
150
Fold.Enrichment

Fig. 5I

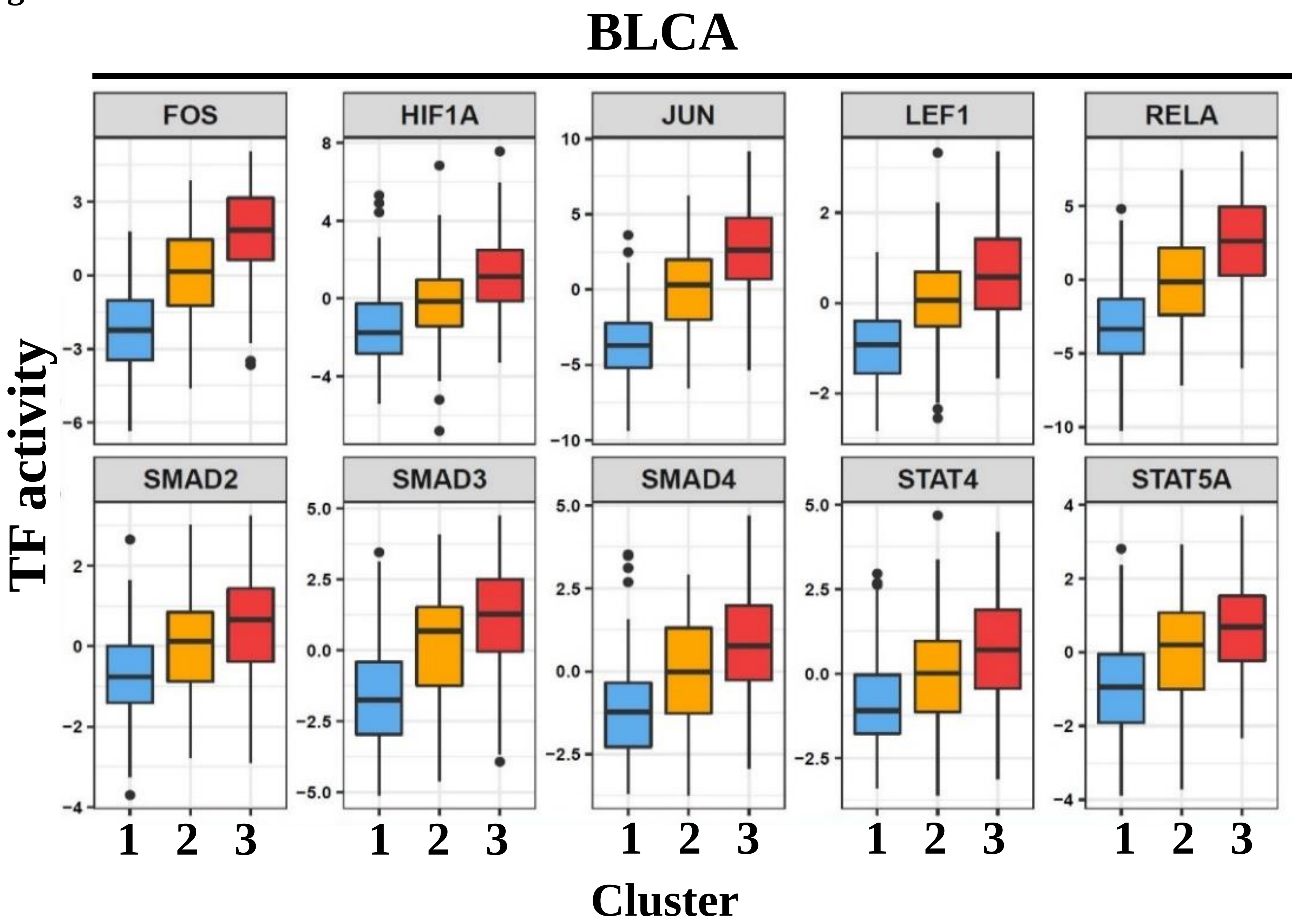

BLCA
FOS
HIF1A
JUN
LEF1
RELA
SMAD2
SMAD3
SMAD4
STAT4
STAT5A
TF activity
1 2 3
Cluster

Fig. 5I

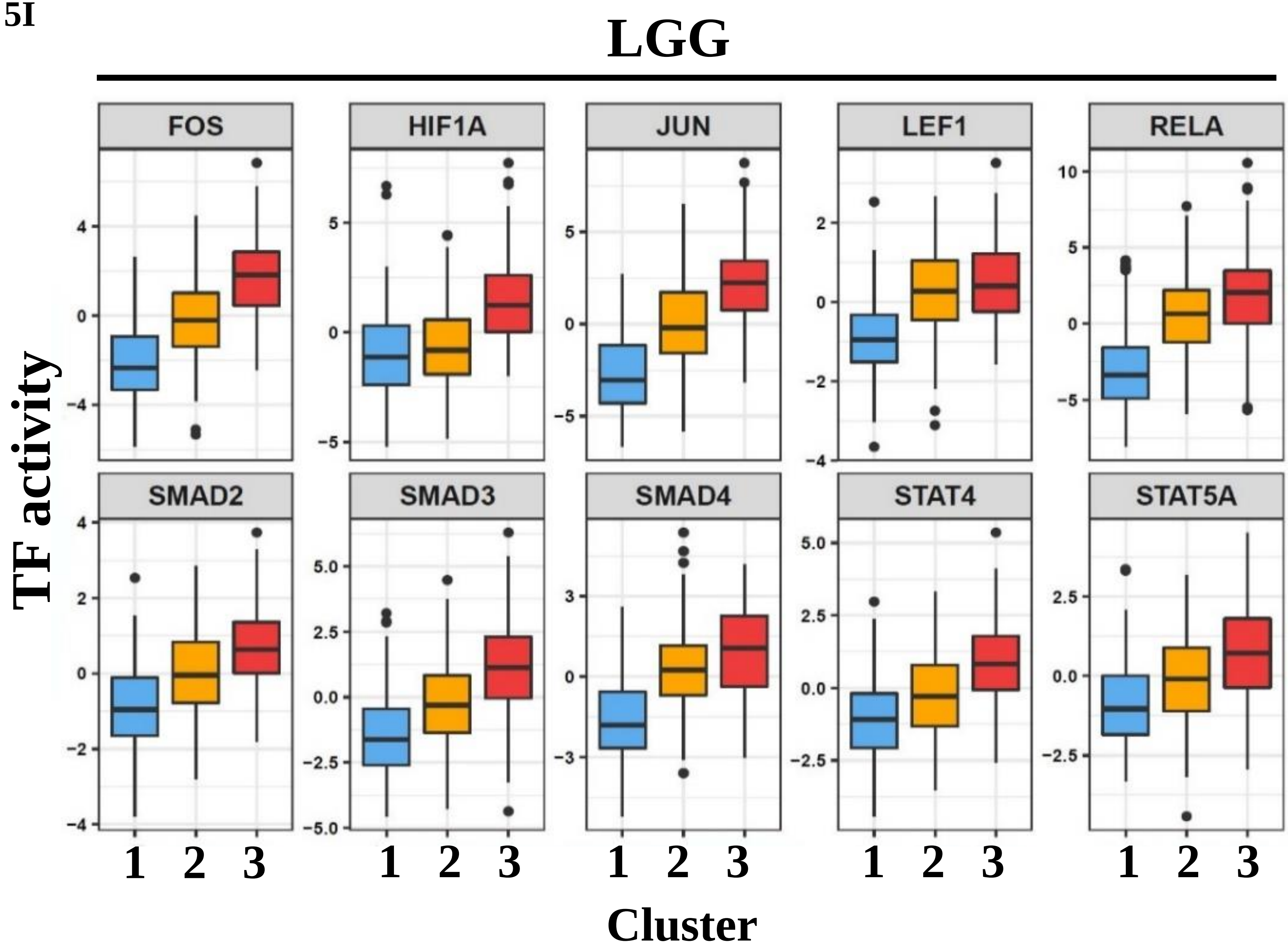
LGG
FOS
HIF1A
JUN
LEF1
RELA
SMAD2
SMAD3
SMAD4
STAT4
STAT5A
TF activity
1 2 3
Cluster

Fig. 5I

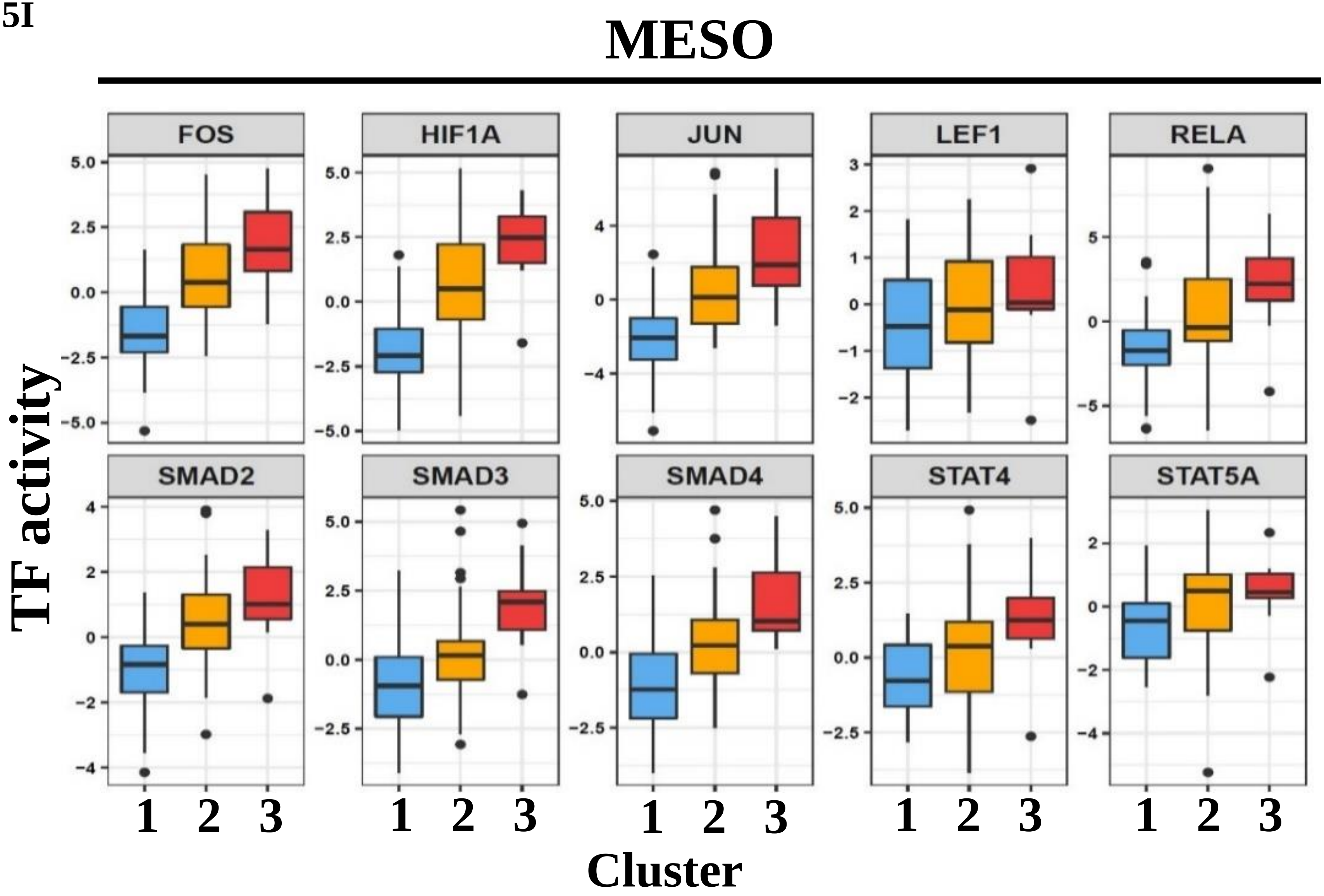

MESO
FOS
HIF1A
JUN
LEF1
RELA
SMAD2
SMAD3
SMAD4
STAT4
STAT5A
TF activity
1 2 3
Cluster

Fig. 5I

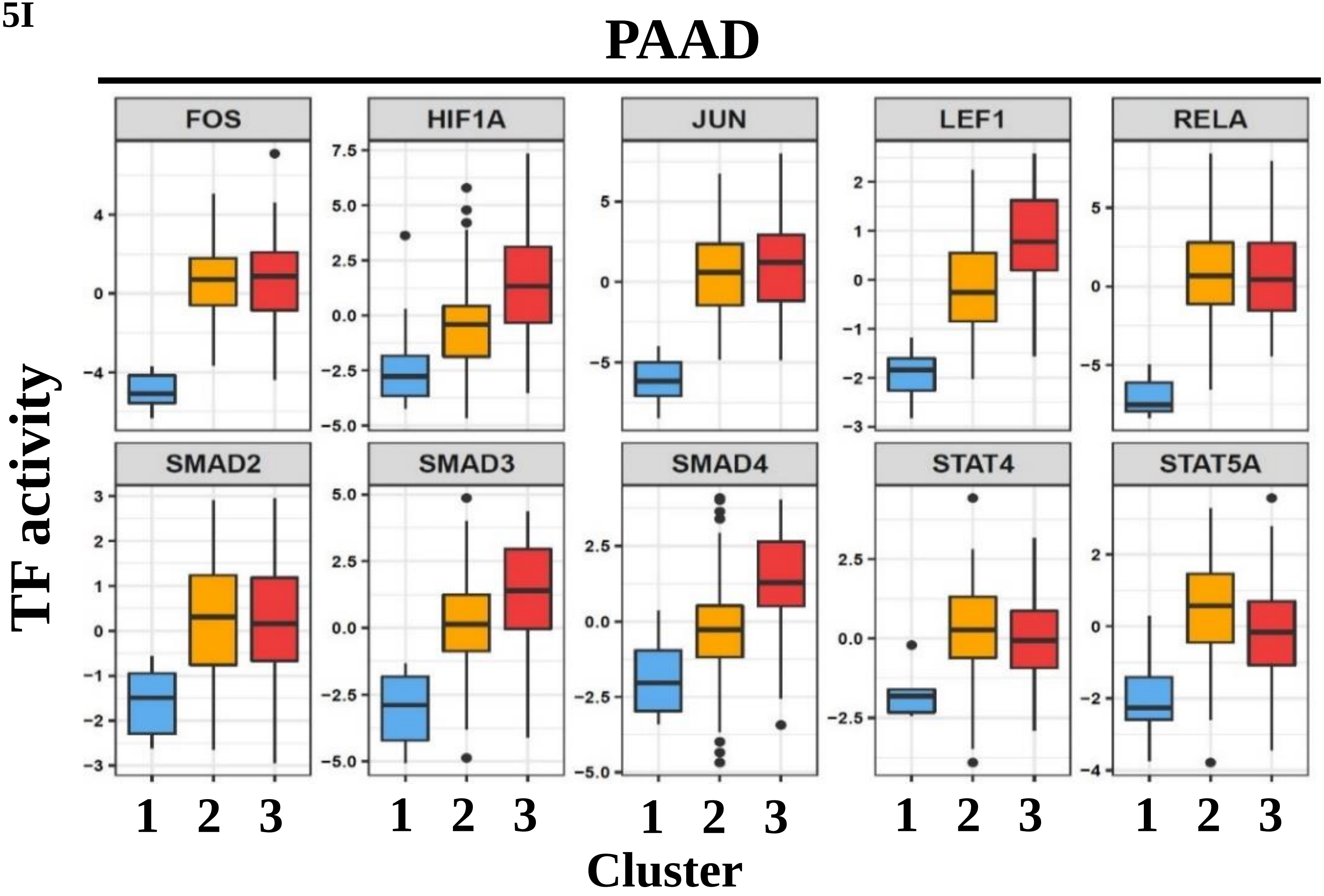
PAAD
FOS
HIF1A
JUN
LEF1
RELA
SMAD2
SMAD3
SMAD4
STAT4
STAT5A
TF activity
1 2 3
Cluster

Fig. 5I

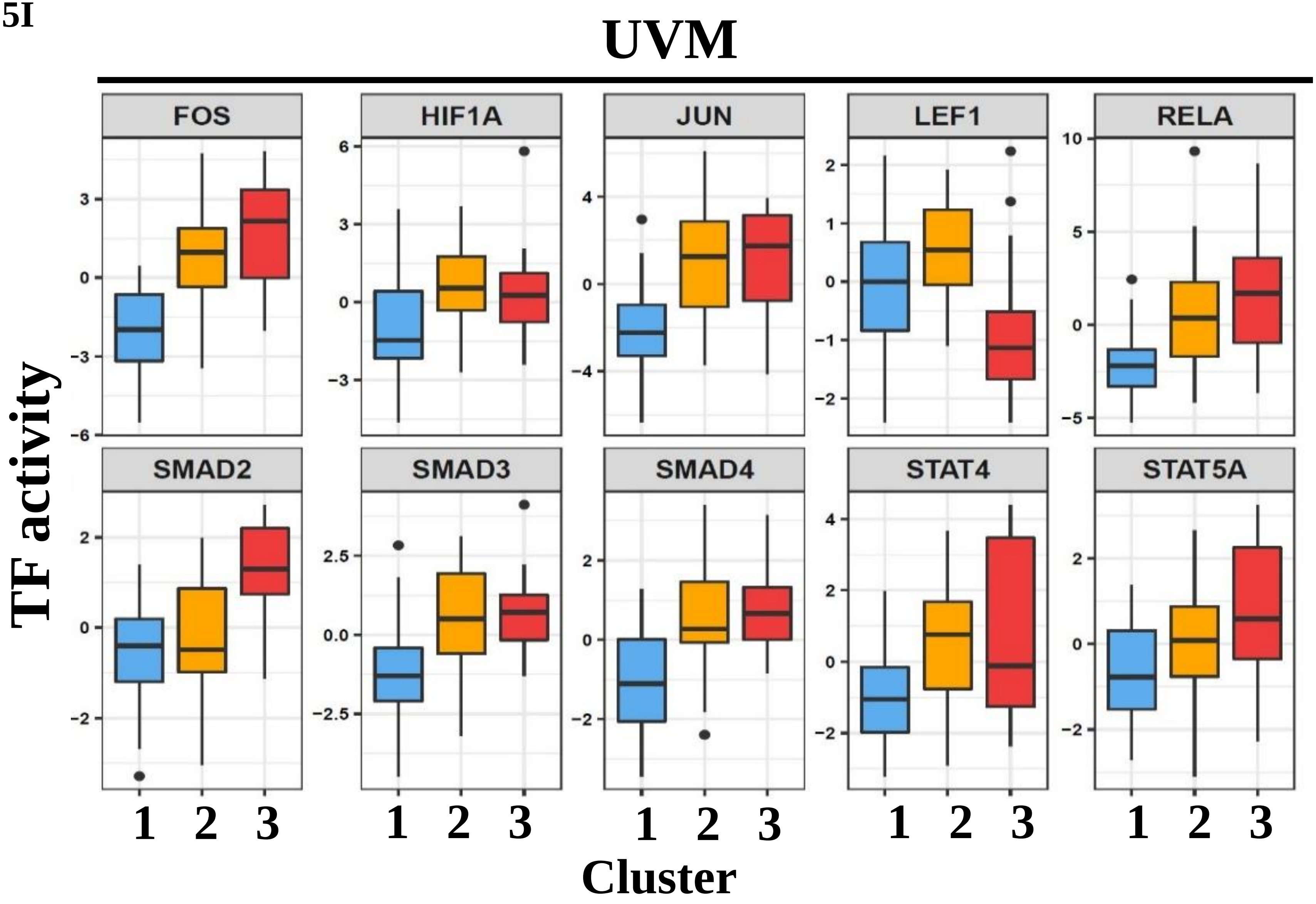

UVM
FOS
HIF1A
JUN
LEF1
RELA
SMAD2
SMAD3
SMAD4
STAT4
STAT5A
TF activity
1 2 3
Cluster

Fig. 5J

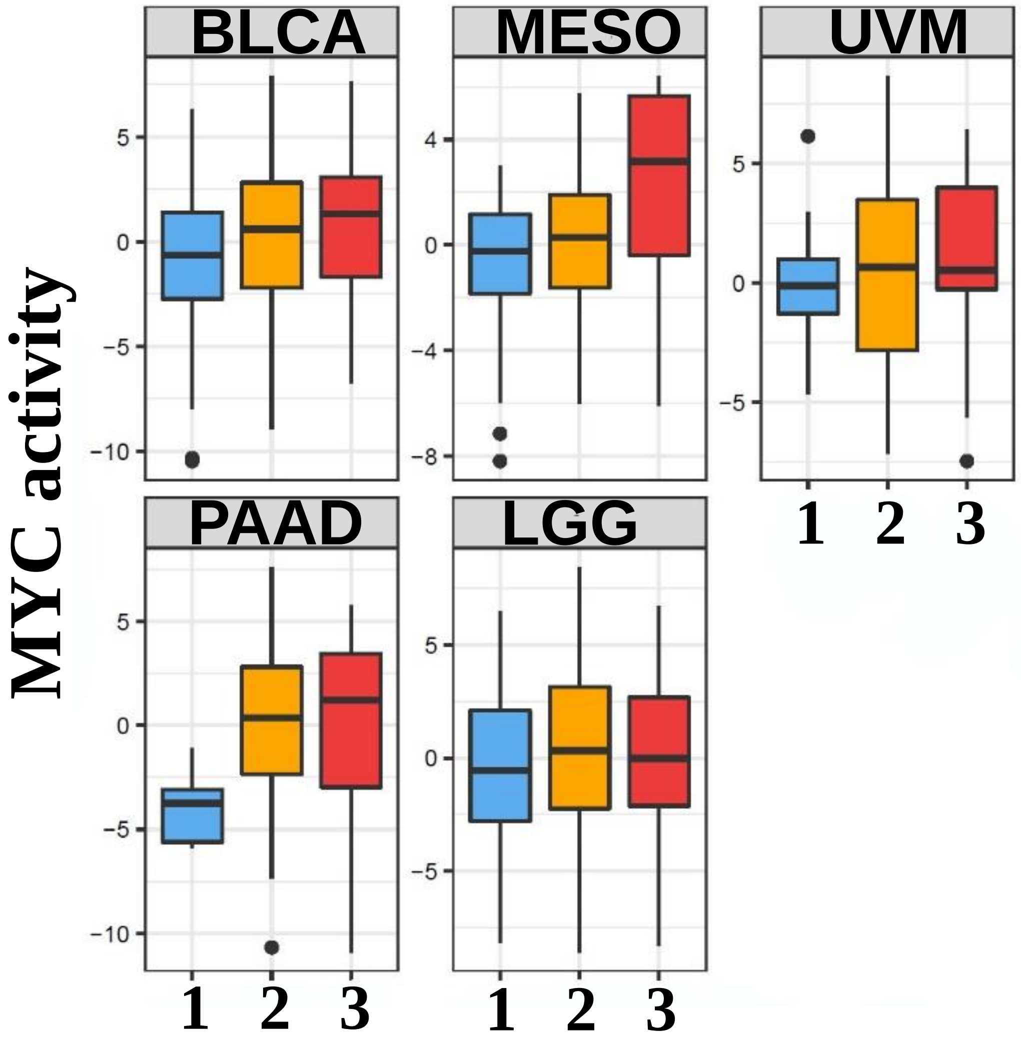

BLCA
MESO
UVM
PAAD
LGG
MYC activity
5
0
−5
−10
4
0
−4
−8
5
0
−5
5
0
−5
−10
5
0
−5
1
2
3
1
2
3
1
2
3

Fig. 5K

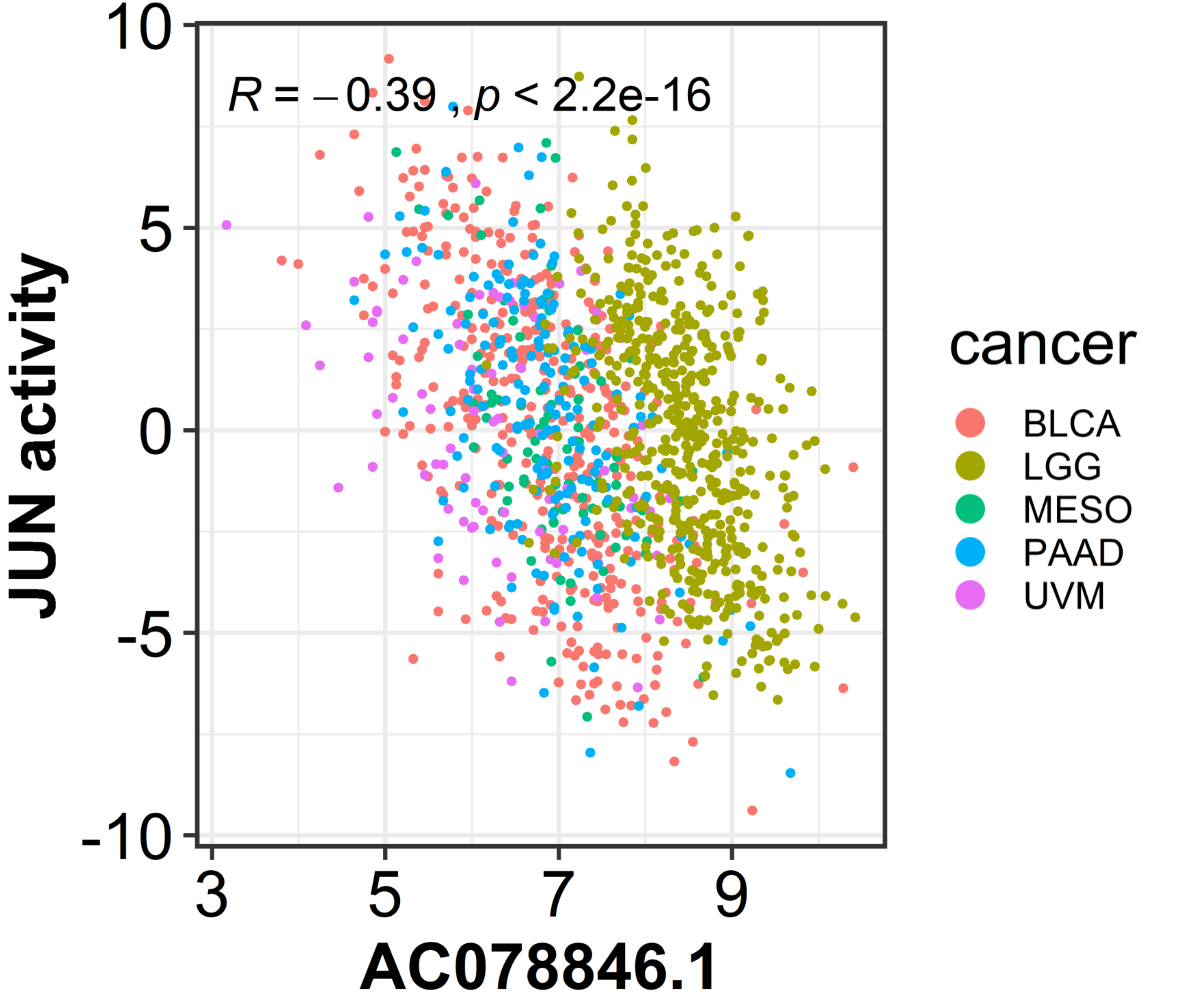
R = – 0.39 , p < 2.2e-16
JUN activity
AC078846.1
10
5
0
-5
-10
3
5
7
9
cancer
BLCA
LGG
MESO
PAAD
UVM

Fig. 5K

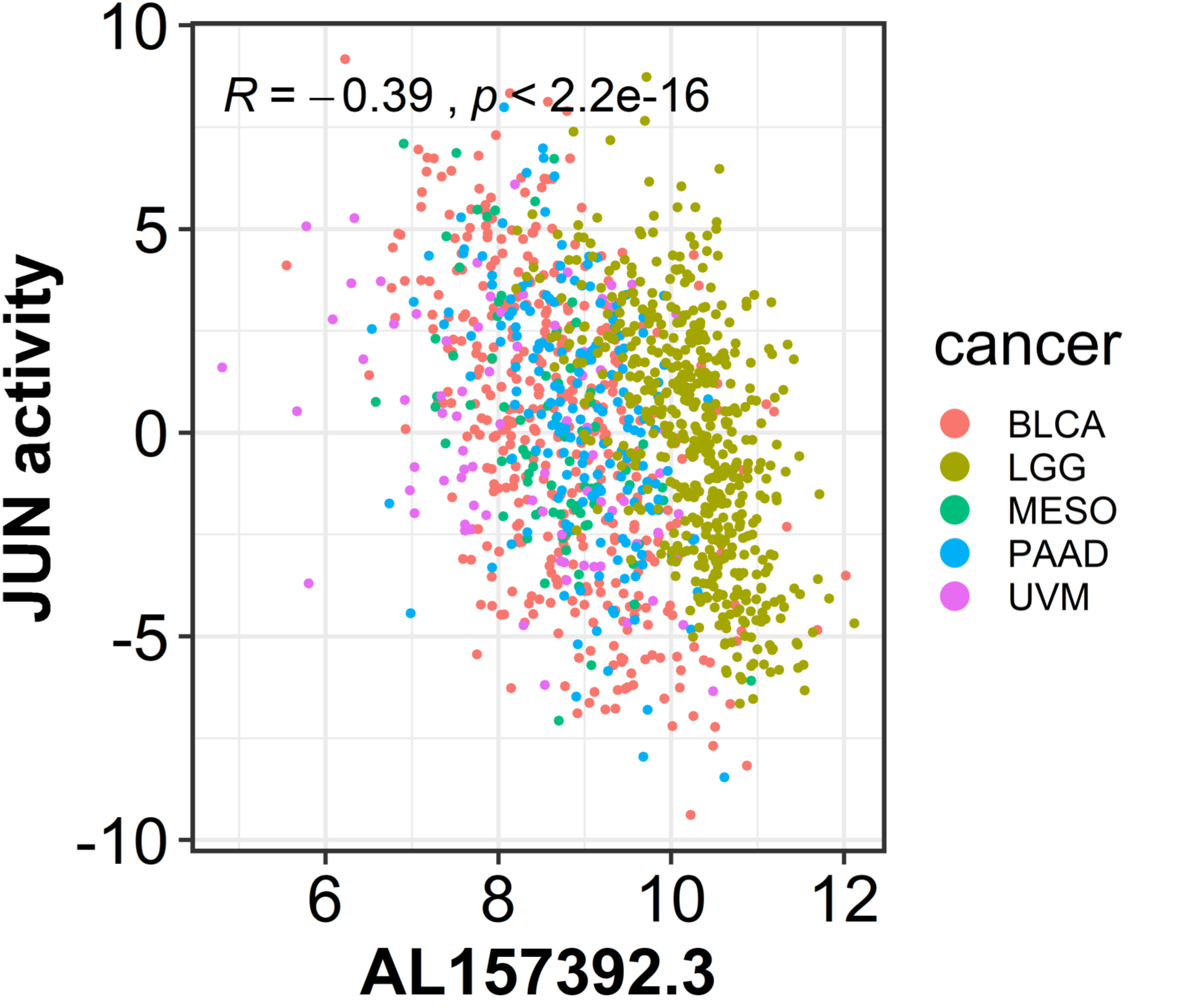
R = – 0.39 , p < 2.2e-16
JUN activity
AL157392.3
10
5
0
-5
-10
6
8
10
12
cancer
BLCA
LGG
MESO
PAAD
UVM

Fig. 5K

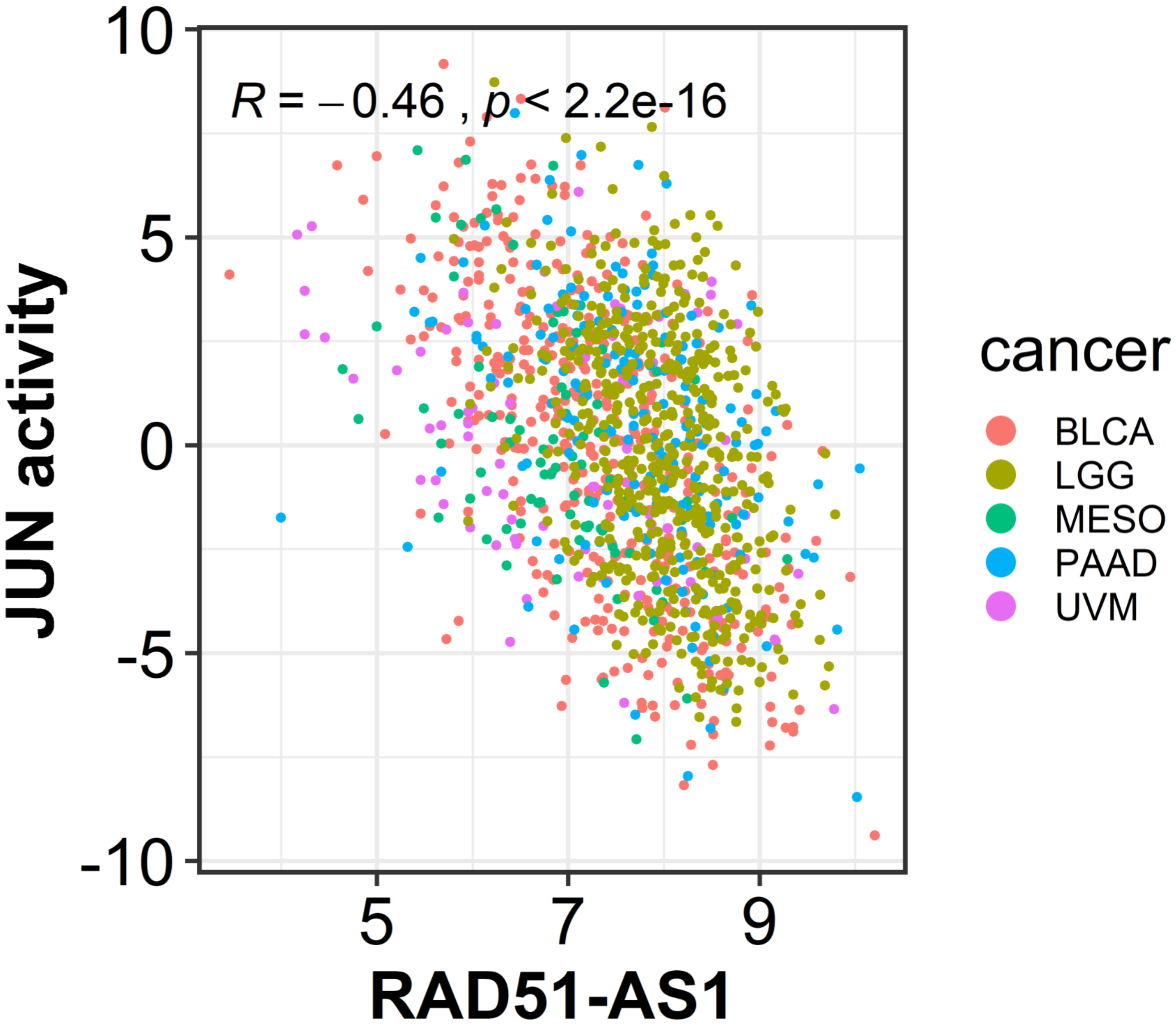
R = −0.46 , p < 2.2e-16
JUN activity
RAD51-AS1
10
5
0
-5
-10
5
7
9
cancer
BLCA
LGG
MESO
PAAD
UVM

Fig. 5K

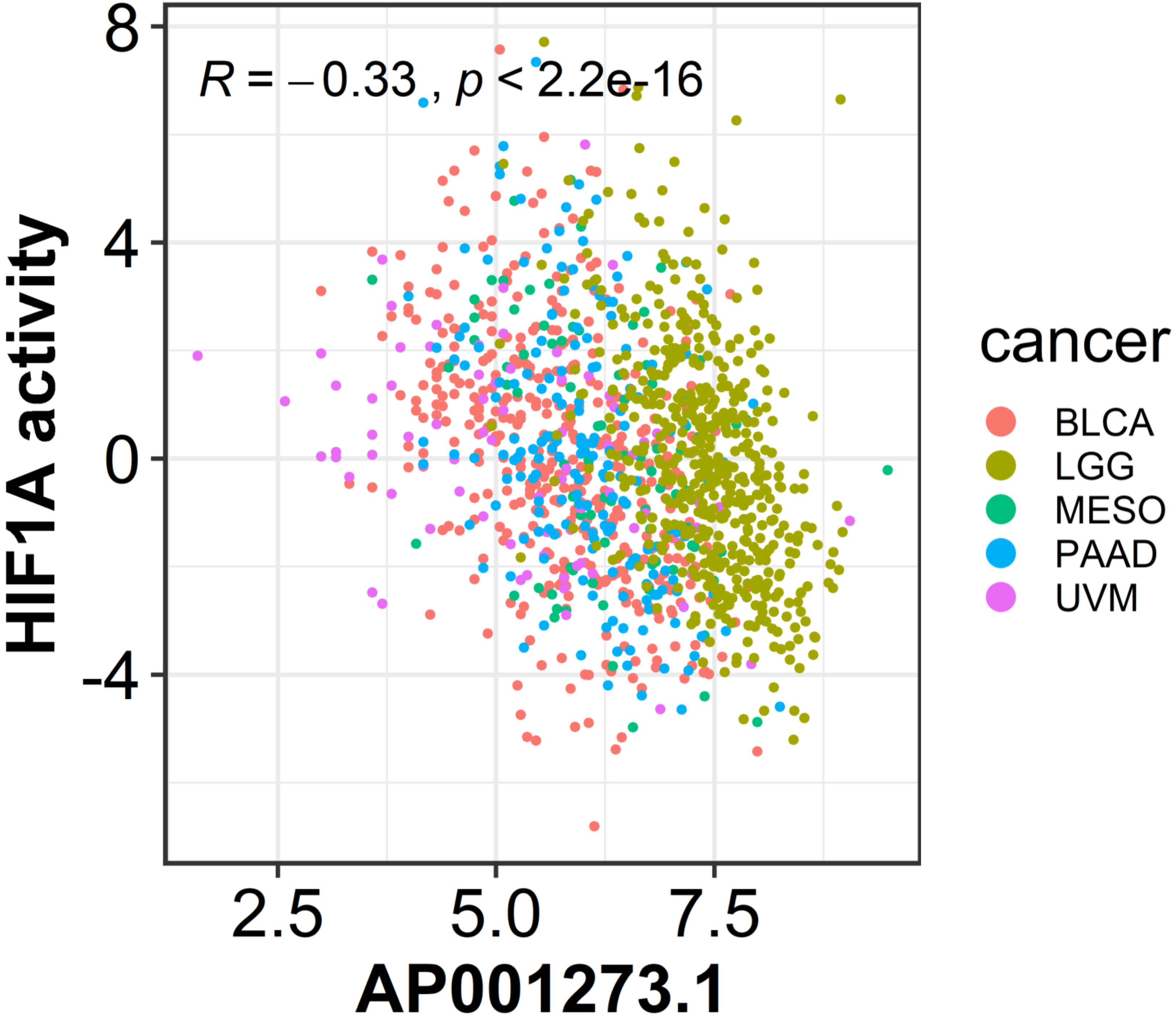

R = −0.33 , p < 2.2e-16
HIF1A activity
8
4
0
-4
2.5
5.0
7.5
AP001273.1
cancer
BLCA
LGG
MESO
PAAD
UVM

Fig. 5K

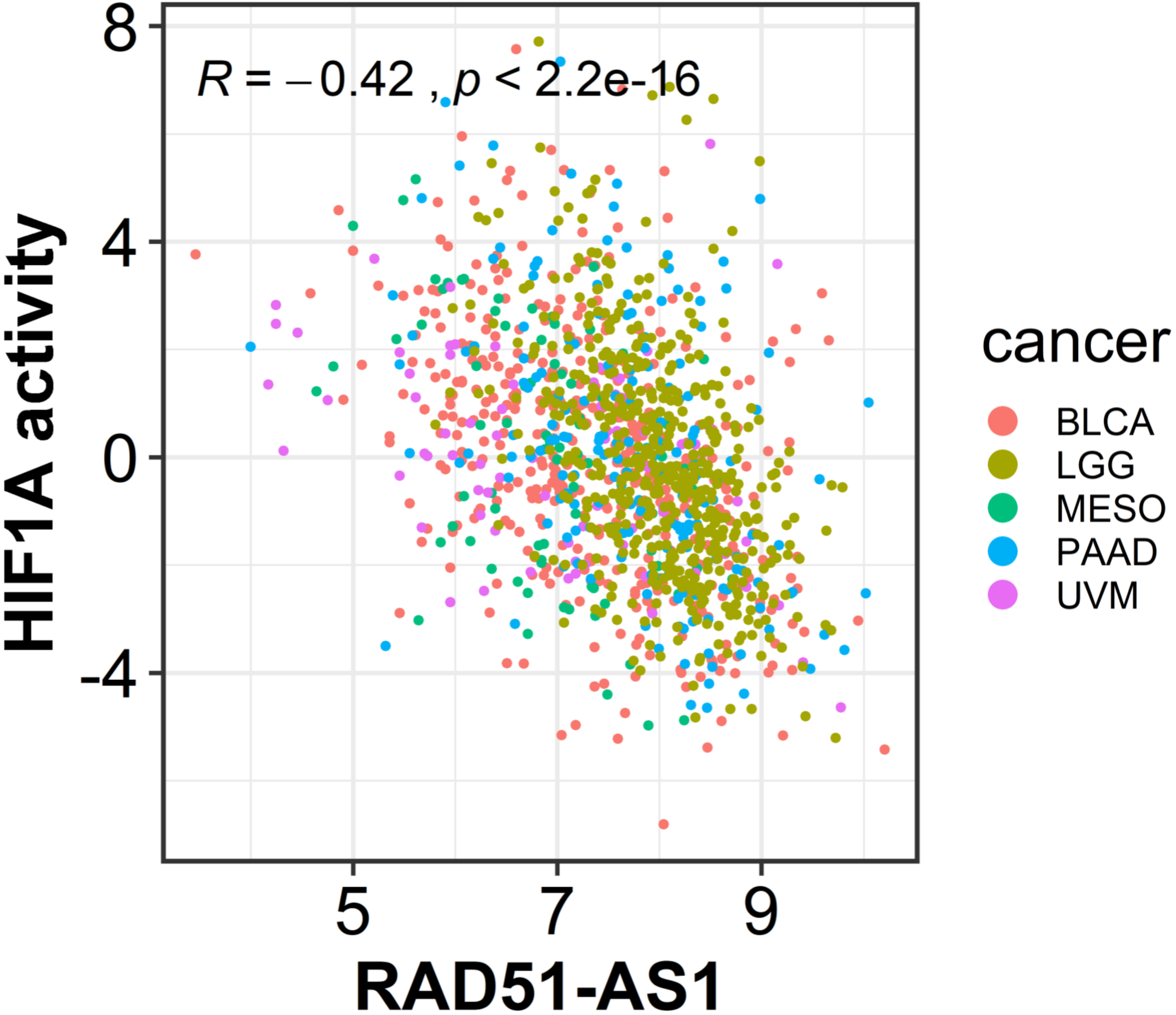
R = –0.42 , p < 2.2e-16
HIF1A activity
8
4
0
-4
5
7
9
RAD51-AS1
cancer
BLCA
LGG
MESO
PAAD
UVM

Fig. 5K

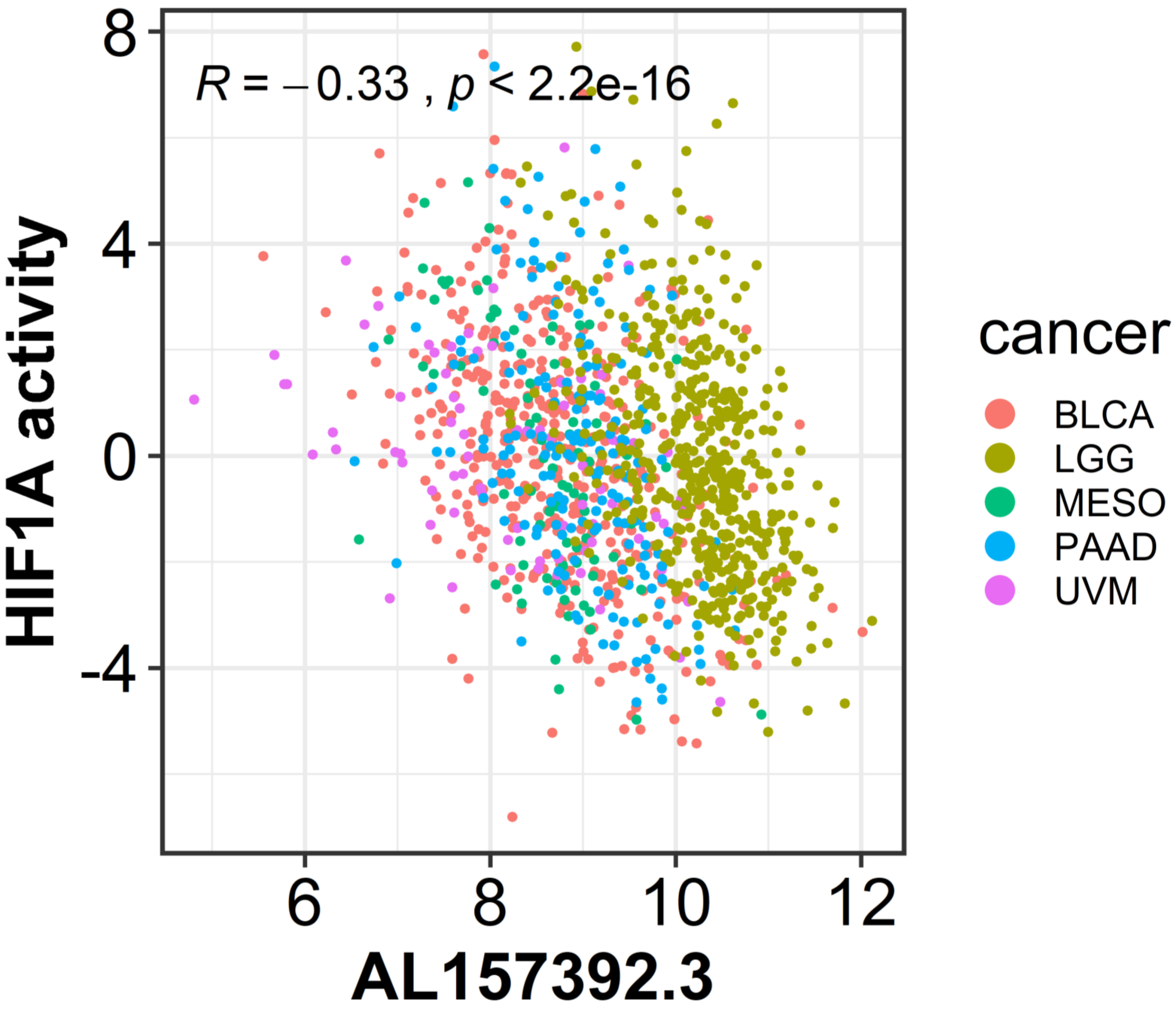

R = –0.33 , p < 2.2e-16
HIF1A activity
AL157392.3
8
4
0
-4
6
8
10
12
cancer
BLCA
LGG
MESO
PAAD
UVM

Fig. 5K

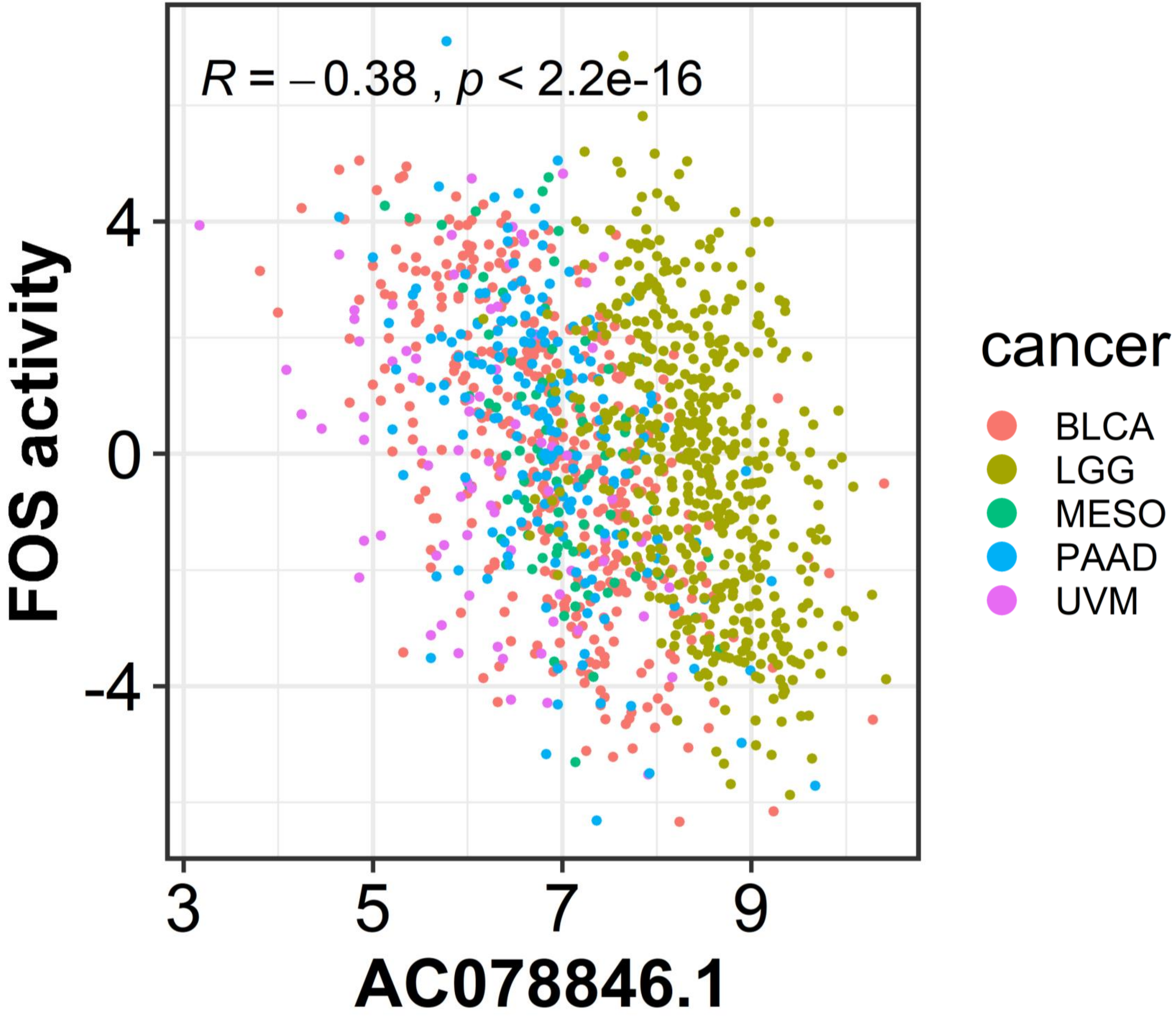
R = −0.38 , p < 2.2e-16
FOS activity
4
0
-4
3
5
7
9
AC078846.1
cancer
BLCA
LGG
MESO
PAAD
UVM

Fig. 5K

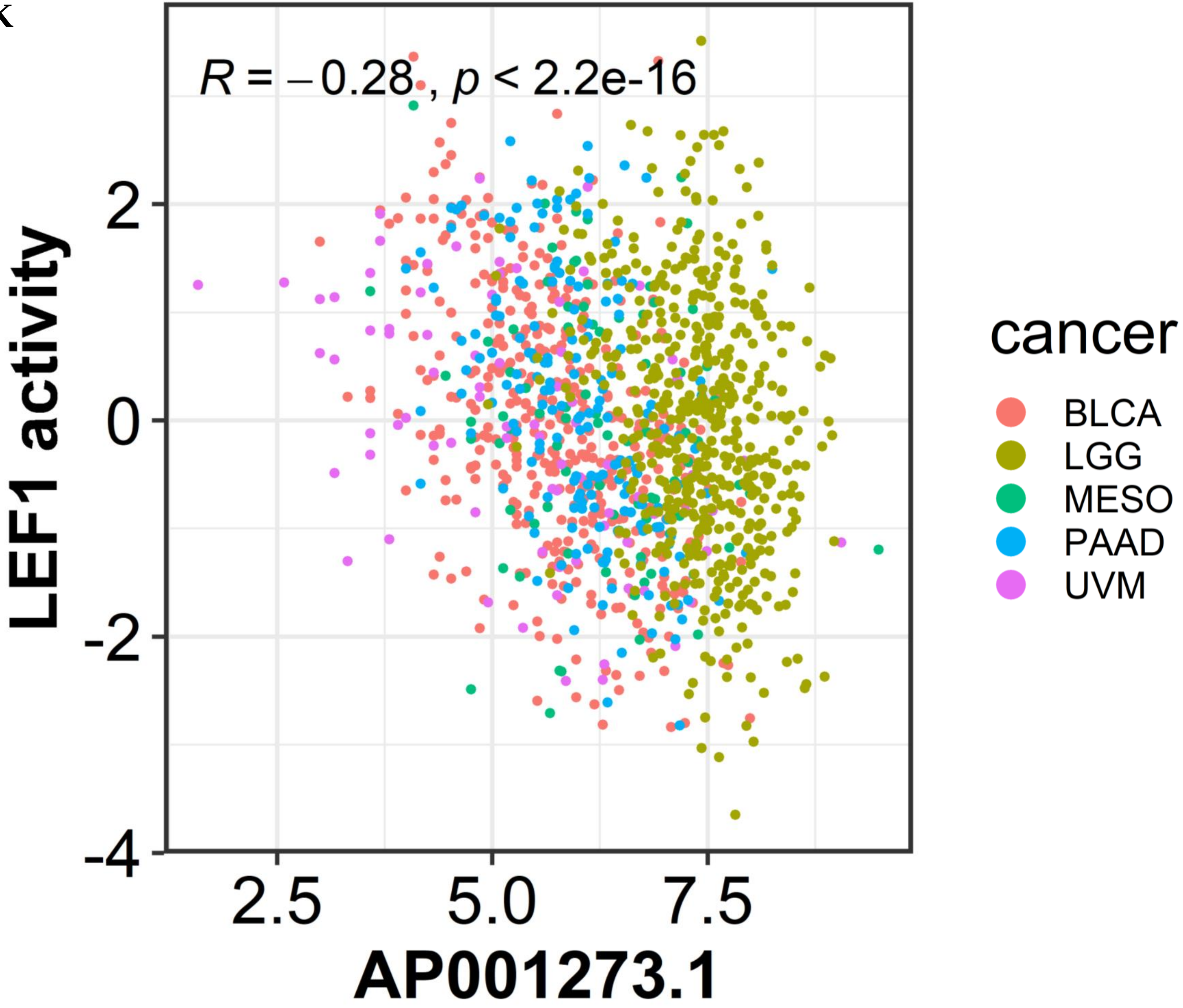
R = –0.28 , p < 2.2e-16
LEF1 activity
2
0
-2
-4
2.5
5.0
7.5
AP001273.1
cancer
BLCA
LGG
MESO
PAAD
UVM

Fig. 6A

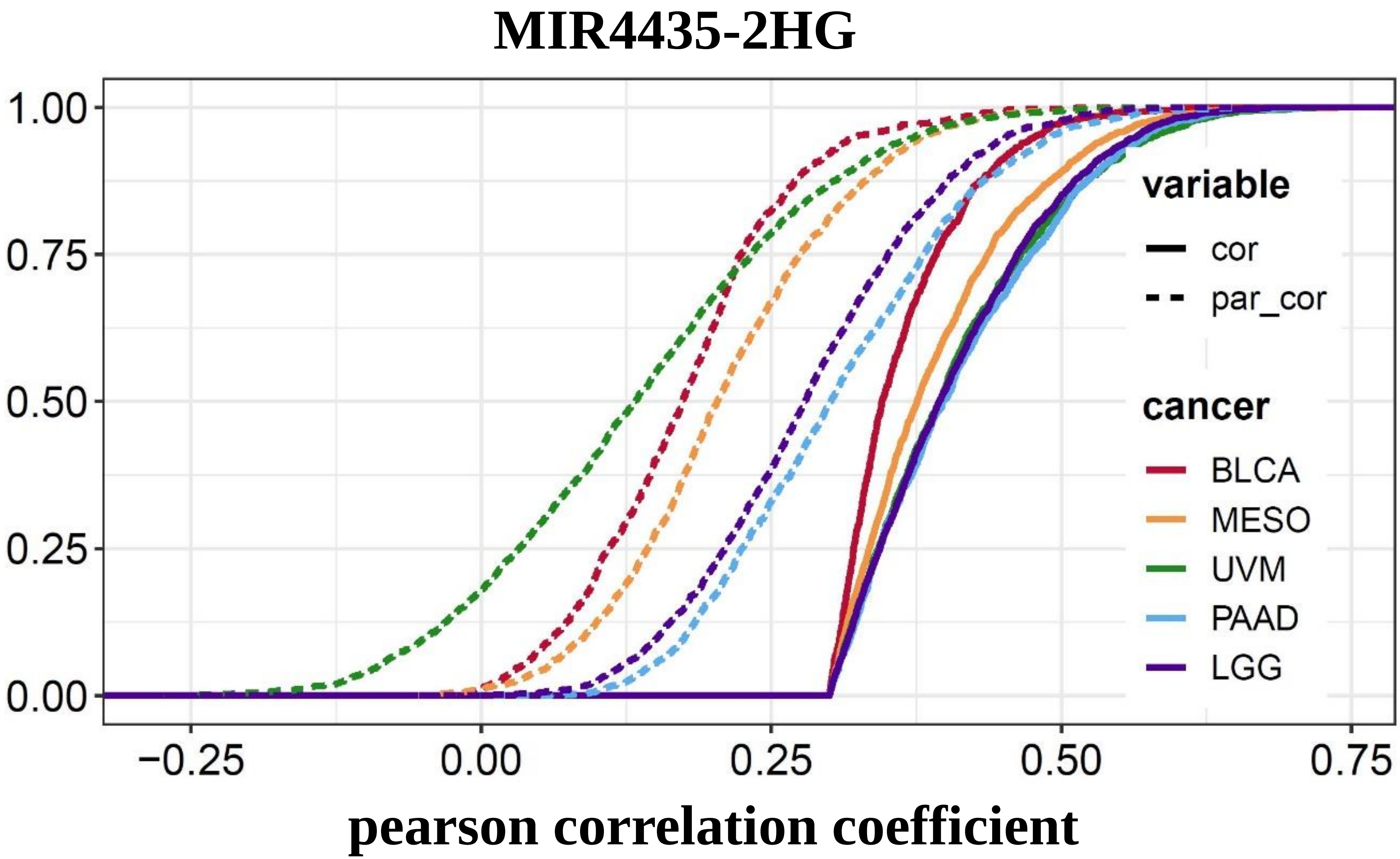
MIR4435-2HG
1.00
0.75
0.50
0.25
0.00
−0.25
0.00
0.25
0.50
0.75
pearson correlation coefficient
variable
cor
par_cor
cancer
BLCA
MESO
UVM
PAAD
LGG

Fig. 6A

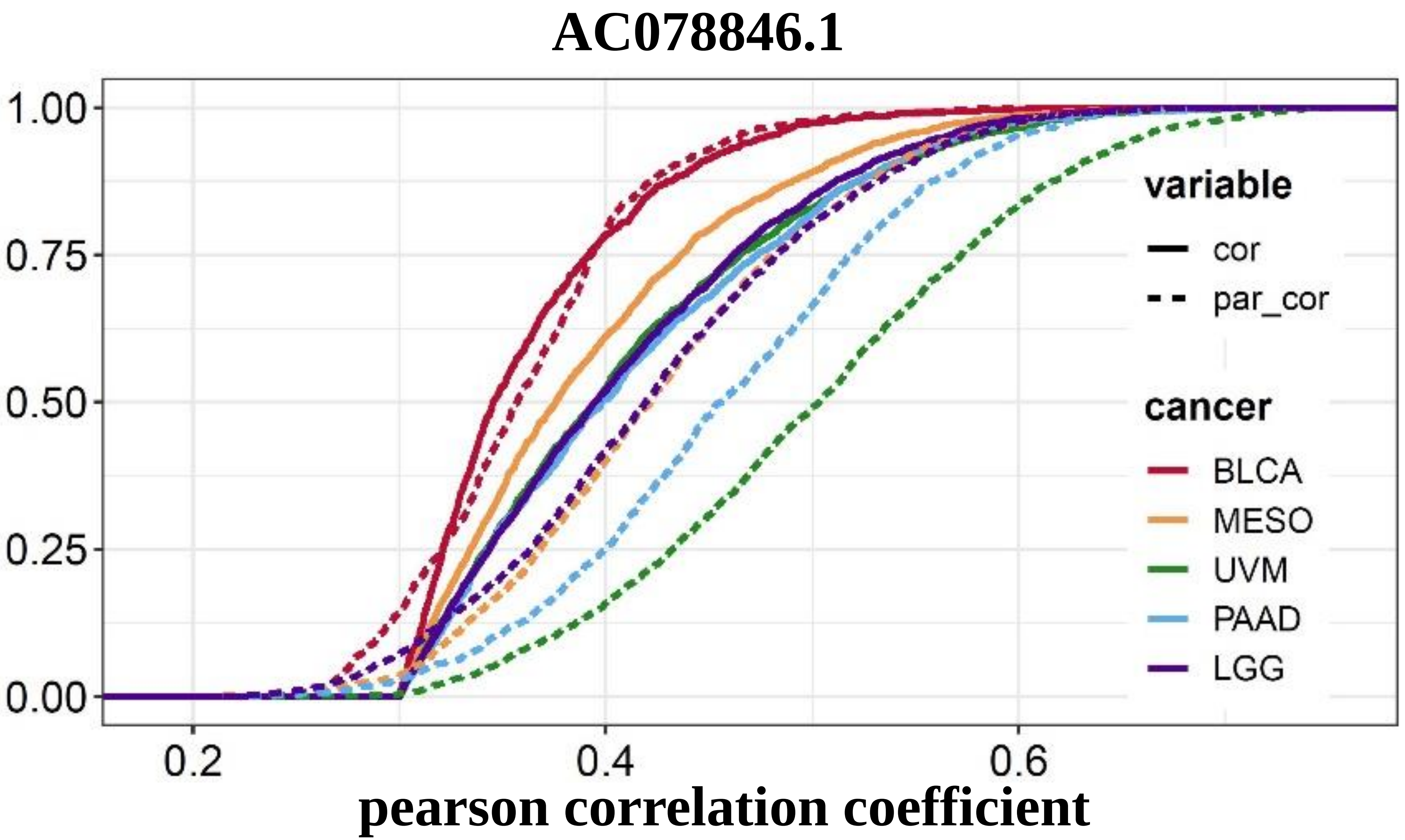
AC078846.1
1.00
0.75
0.50
0.25
0.00
0.2
0.4
0.6
variable
cor
par_cor
cancer
BLCA
MESO
UVM
PAAD
LGG
pearson correlation coefficient

Fig. 6A

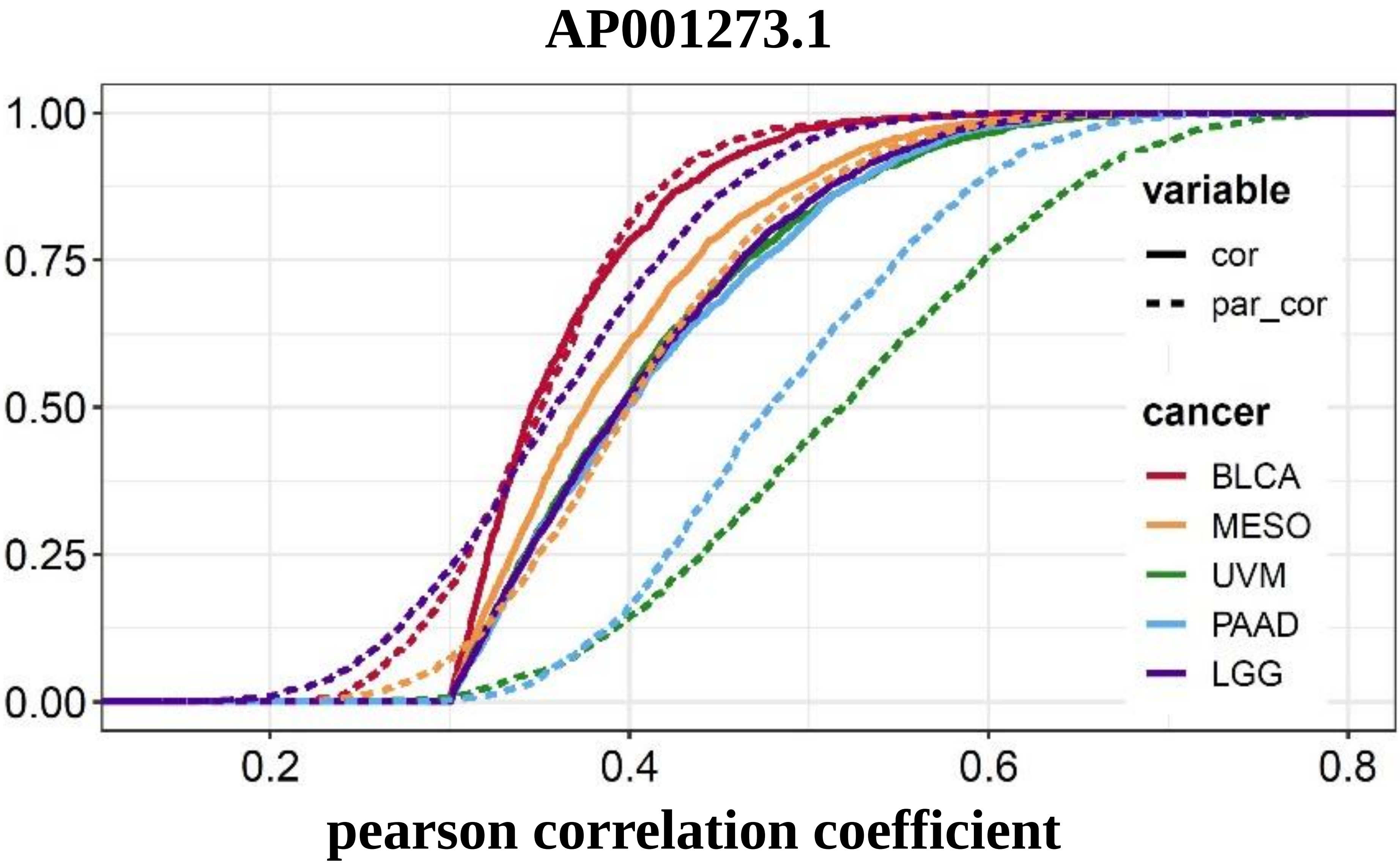

AP001273.1
1.00
0.75
0.50
0.25
0.00
0.2
0.4
0.6
0.8
pearson correlation coefficient
variable
cor
par_cor
cancer
BLCA
MESO
UVM
PAAD
LGG

Fig. 6A

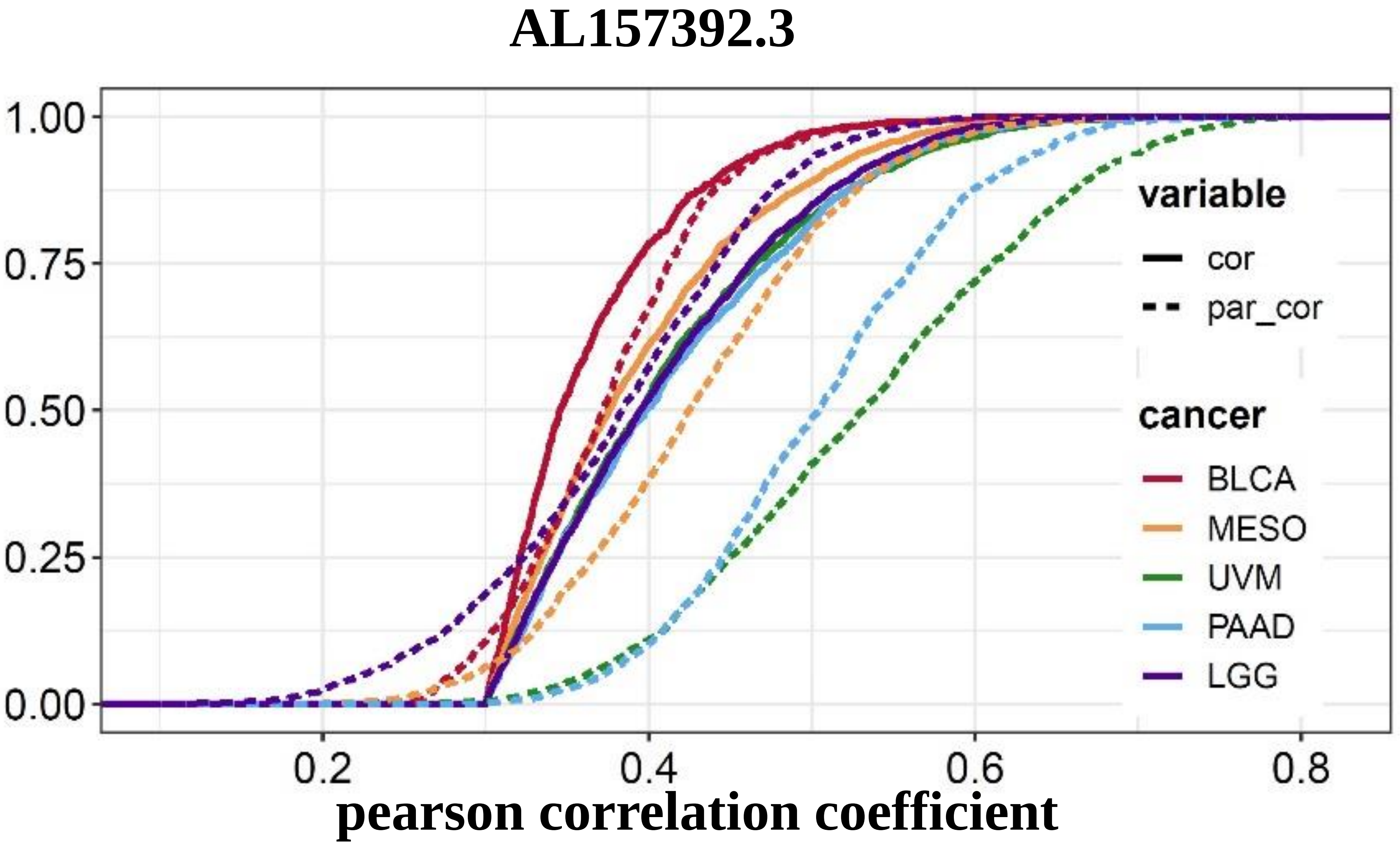
AL157392.3
1.00
0.75
0.50
0.25
0.00
0.2
0.4
0.6
0.8
pearson correlation coefficient
variable
cor
par_cor
cancer
BLCA
MESO
UVM
PAAD
LGG

Fig. 6A

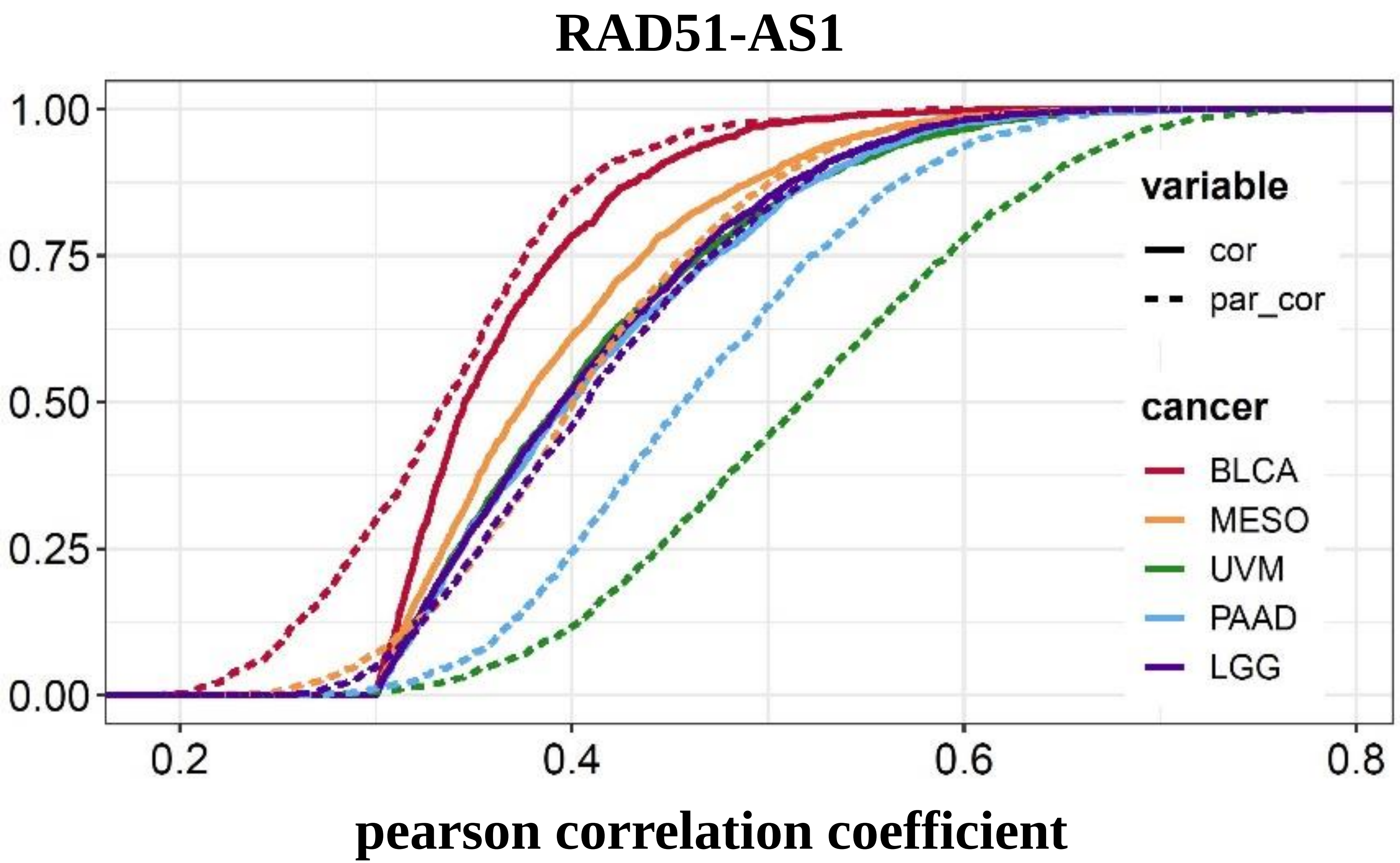

RAD51-AS1
1.00
0.75
0.50
0.25
0.00
0.2
0.4
0.6
0.8
variable
cor
par_cor
cancer
BLCA
MESO
UVM
PAAD
LGG
pearson correlation coefficient

Fig. 6B

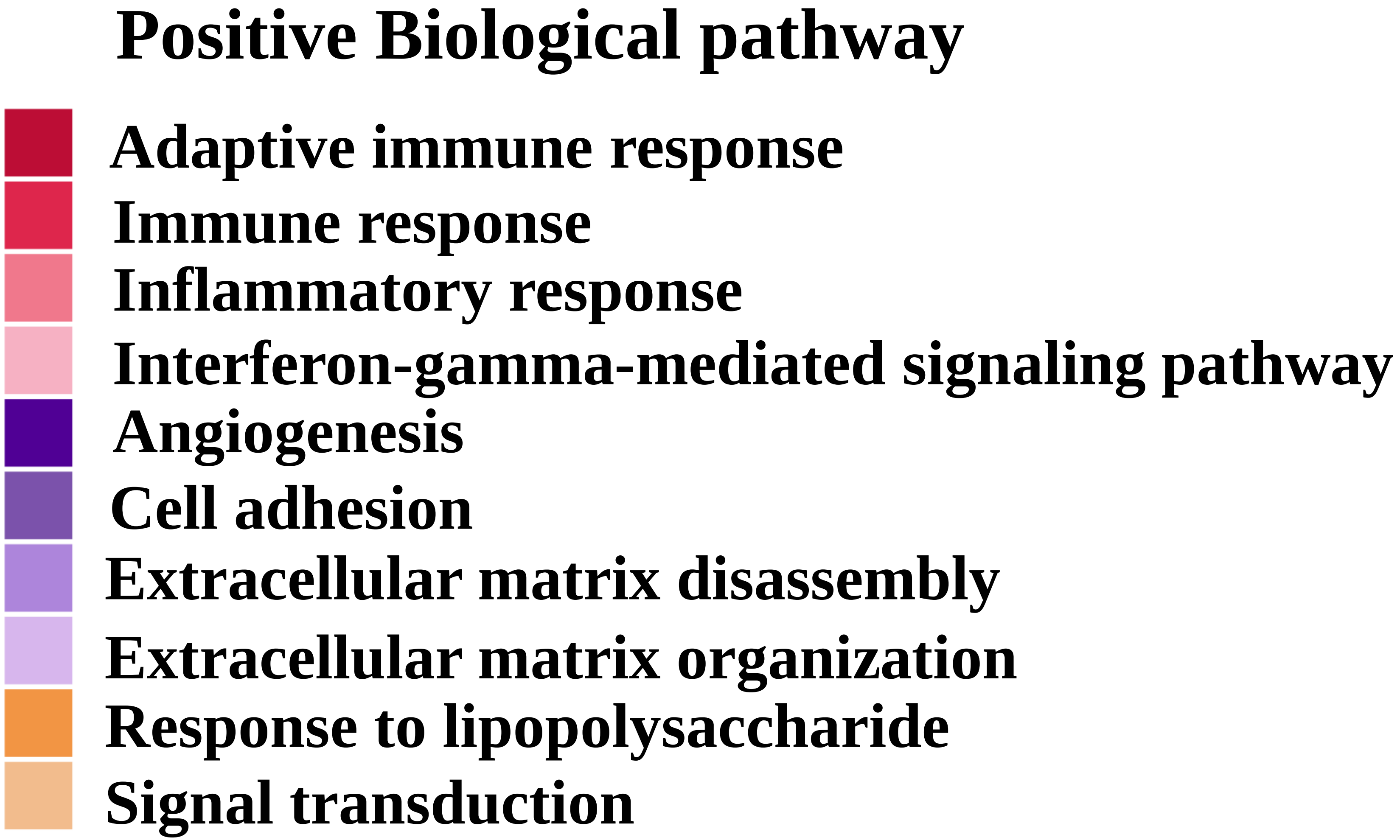
Positive Biological pathway
Adaptive immune response
Immune response
Inflammatory response
Interferon-gamma-mediated signaling pathway
Angiogenesis
Cell adhesion
Extracellular matrix disassembly
Extracellular matrix organization
Response to lipopolysaccharide
Signal transduction

Fig. 6B

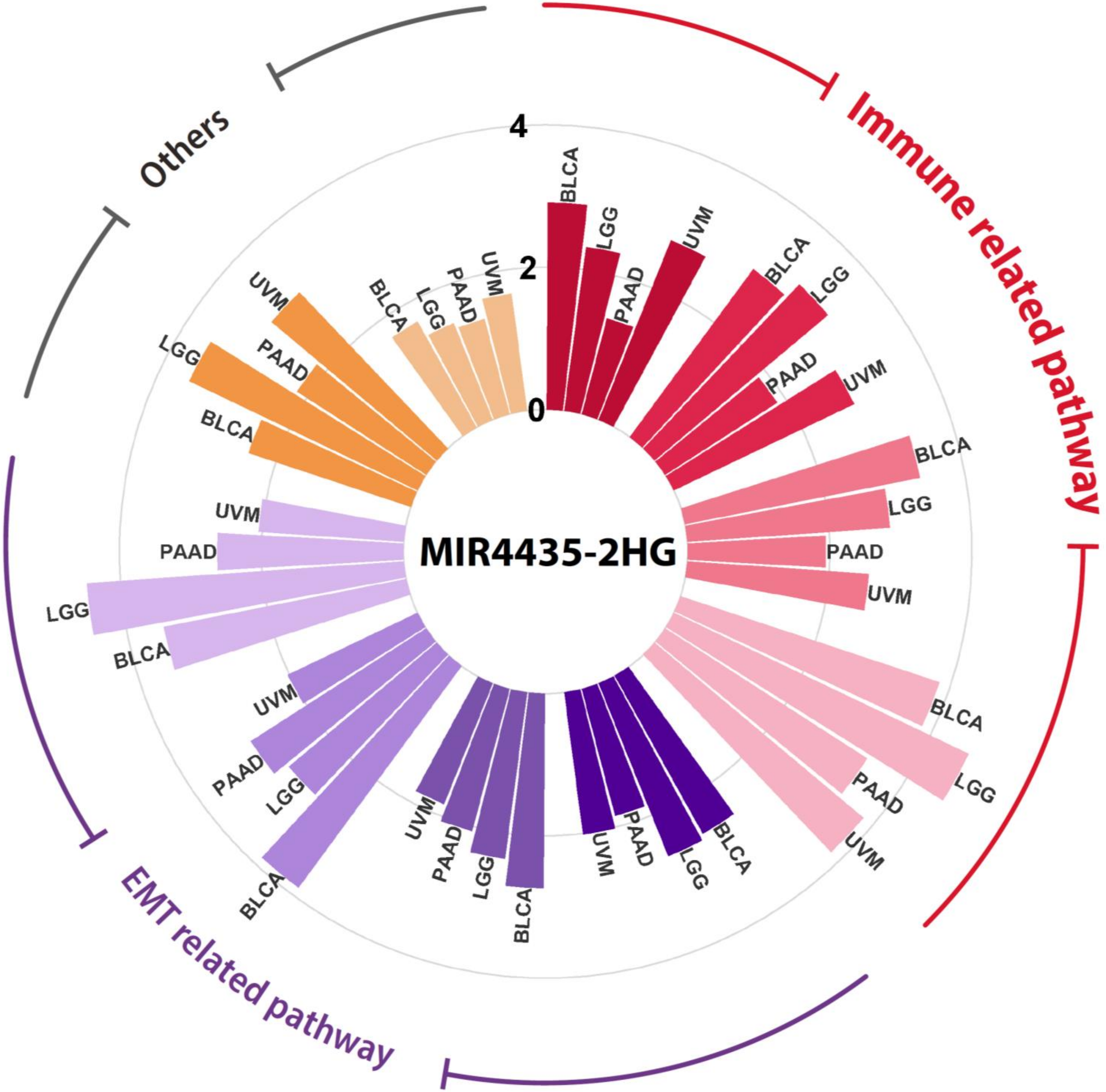

Others
Immune related pathway
EMT related pathway
MIR4435-2HG
0
2
4
BLCA
LGG
PAAD
UVM

Fig. 6C

BLCA

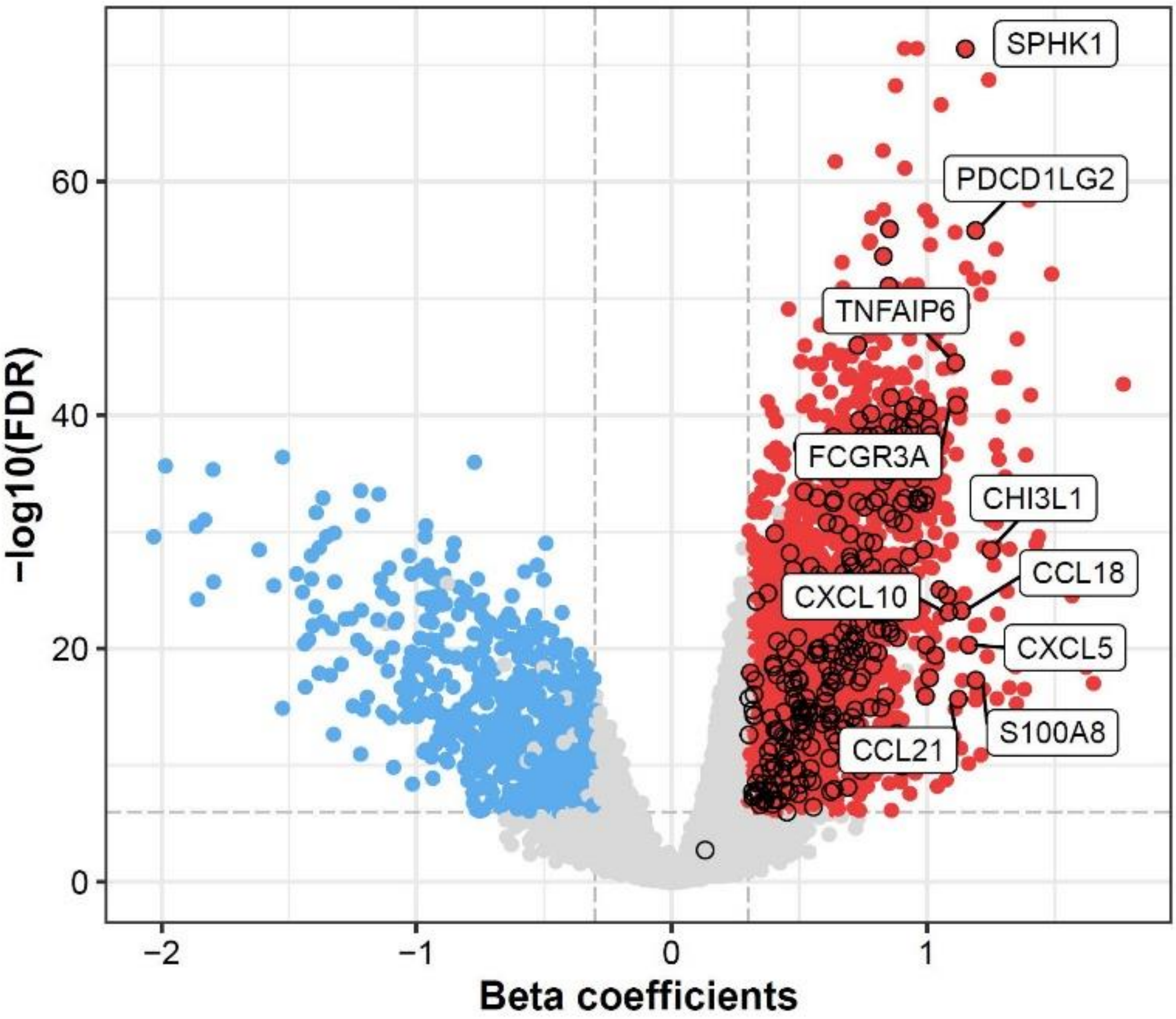

SPHK1
PDCD1LG2
TNFAIP6
FCGR3A
CHI3L1
CCL18
CXCL10
CXCL5
S100A8
CCL21
60
40
20
0
-log10(FDR)
−2
−1
0
1
Beta coefficients

Fig. 6C

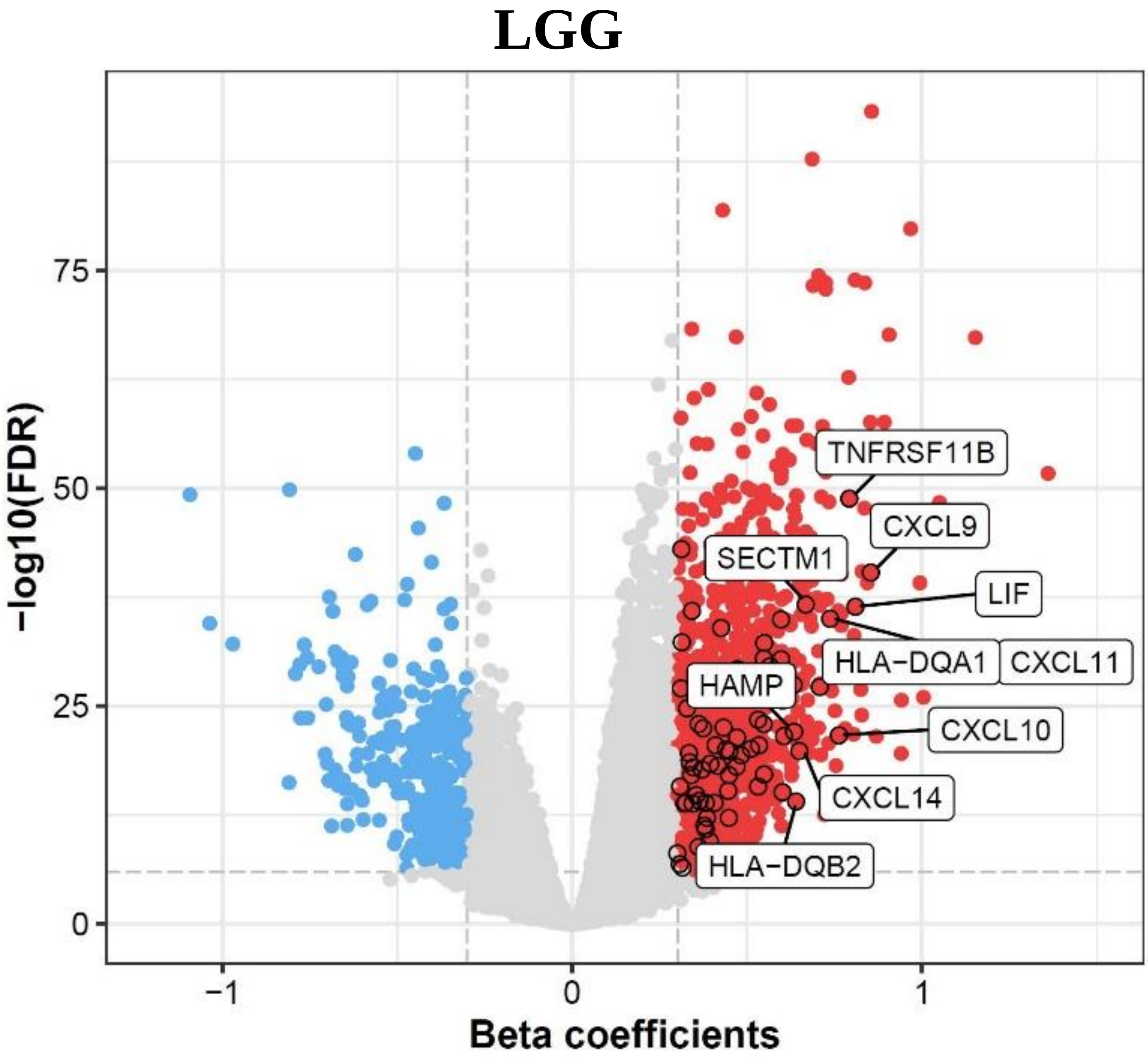

LGG
-log10(FDR)
75
50
25
0
−1
0
1
Beta coefficients
TNFRSF11B
CXCL9
SECTM1
LIF
HLA−DQA1
CXCL11
HAMP
CXCL10
CXCL14
HLA−DQB2

Fig. 6C

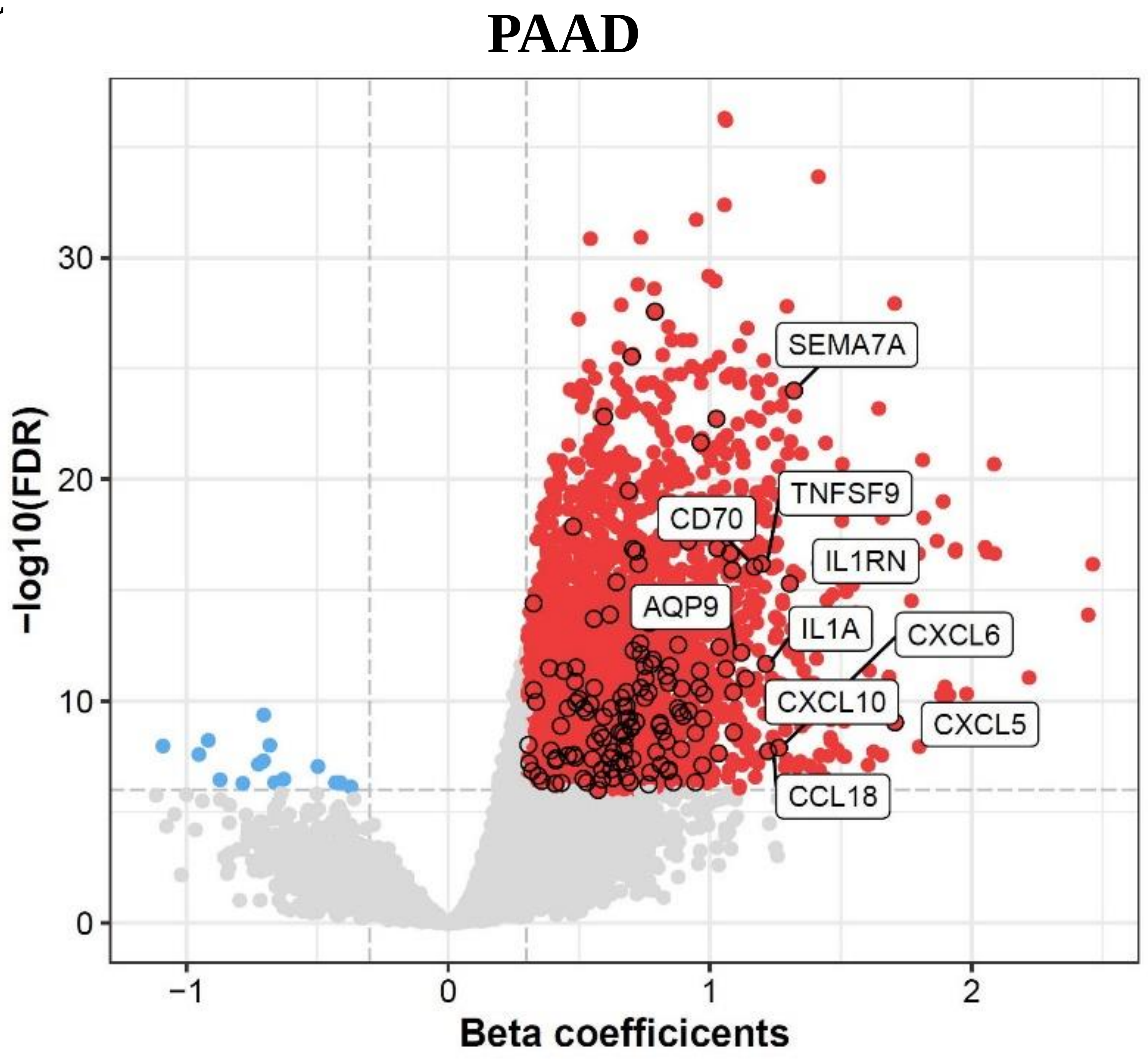
PAAD
SEMA7A
TNFSF9
CD70
IL1RN
AQP9
IL1A
CXCL6
CXCL10
CXCL5
CCL18
-log10(FDR)
30
20
10
0
−1
0
1
2
Beta coefficicents

Fig. 6C

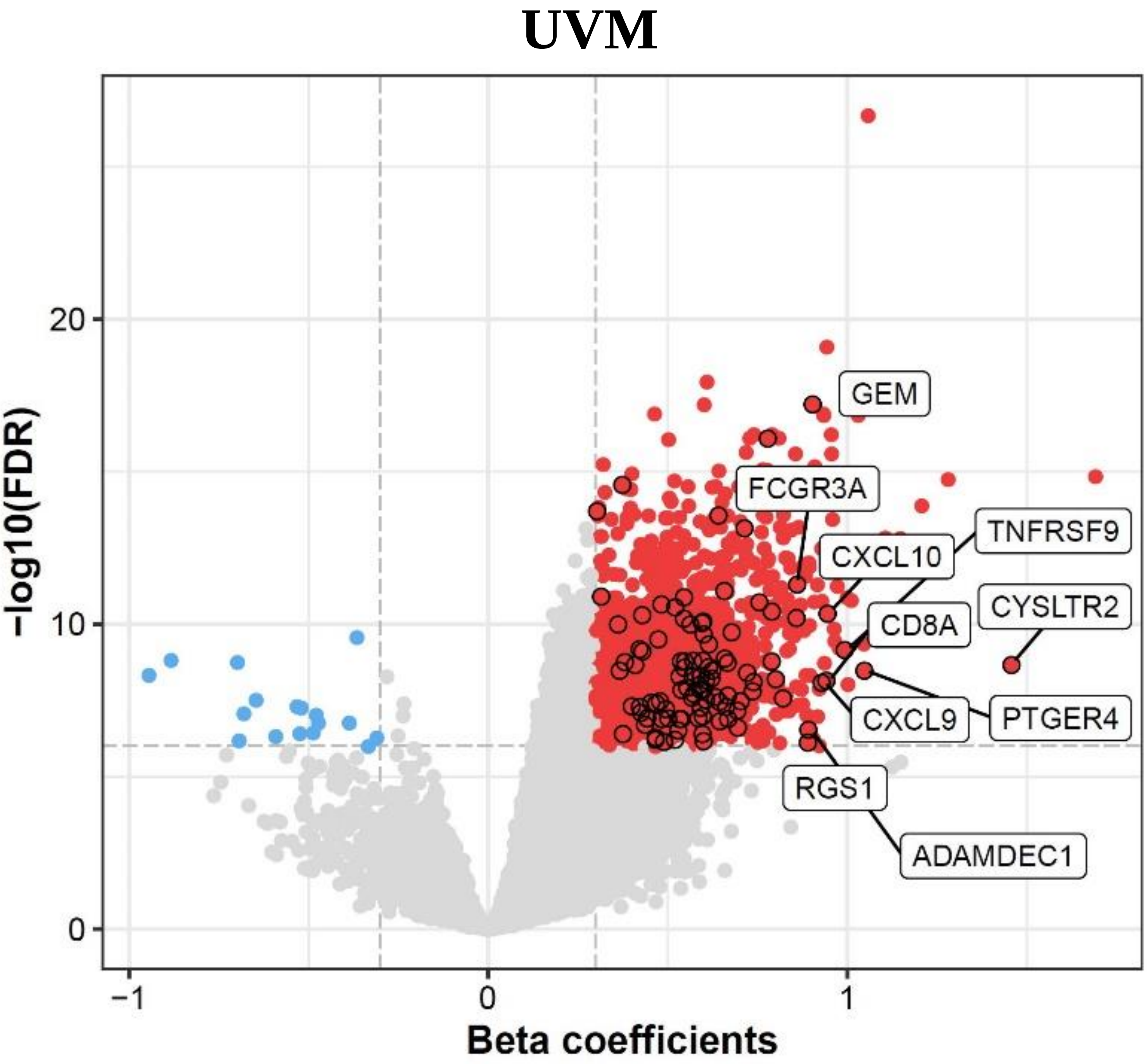
UVM
GEM
FCGR3A
TNFRSF9
CXCL10
CD8A
CYSLTR2
CXCL9
PTGER4
RGS1
ADAMDEC1
-log10(FDR)
20
10
0
−1
0
1
Beta coefficients
